# Effect of AZD0530 on Cerebral Metabolic Decline in Alzheimer Disease: A Randomized Clinical Trial

**DOI:** 10.1001/jamaneurol.2019.2050

**Published:** 2019-07-22

**Authors:** Christopher H. van Dyck, Haakon B. Nygaard, Kewei Chen, Michael C. Donohue, Rema Raman, Robert A. Rissman, James B. Brewer, Robert A. Koeppe, Tiffany W. Chow, Michael S. Rafii, Devon Gessert, Jiyoon Choi, R. Scott Turner, Jeffrey A. Kaye, Seth A. Gale, Eric M. Reiman, Paul S. Aisen, Stephen M. Strittmatter

**Affiliations:** 1 Alzheimer’s Disease Research Unit, Yale School of Medicine, New Haven, Connecticut; 2 Division of Neurology, University of British Columbia, Vancouver, British Columbia, Canada; 3 Banner Alzheimer’s Institute, Phoenix, Arizona; 4 Alzheimer’s Therapeutic Research Institute, University of Southern California, San Diego; 5 Department of Neurosciences, University of California, San Diego, La Jolla, California; 6 Department of Radiology, University of Michigan, Ann Arbor; 7 Department of Neurology, Georgetown University, Washington, DC; 8 Department of Neurology, Oregon Health & Science University, Portland; 9 Department of Neurology, Brigham and Women's Hospital, Harvard Medical School, Boston, Massachusetts; 10 Department of Neurology, Yale School of Medicine, New Haven, Connecticut

## Abstract

**Importance:**

Oligomeric amyloid-β peptide binds to cellular prion protein on the neuronal cell surface, activating intracellular fyn kinase to mediate synaptotoxicity and tauopathy. AZD0530 is an investigational kinase inhibitor specific for the Src family, including fyn, that has been repurposed for the treatment of Alzheimer disease.

**Objective:**

To determine whether AZD0530 treatment slows the decline in cerebral metabolic rate for glucose (CMRgl) and is safe and well tolerated.

**Design, Setting, and Participants:**

This multicenter phase 2a randomized clinical trial enrolled participants between December 23, 2014, and November 30, 2016. Participants (n = 159) had mild Alzheimer dementia and positron emission tomography (PET) evidence of elevated levels of amyloid-β peptide. Efficacy analyses of all primary and secondary outcomes were conducted in a modified intention-to-treat population. Final analyses were conducted from February 9, 2018, to July 25, 2018.

**Interventions:**

AZD0530 (100 mg or 125 mg daily) vs placebo for 52 weeks.

**Main Outcomes and Measures:**

Primary outcome was the reduction in relative CMRgl, as measured by ^18^F-fluorodeoxyglucose (^18^F-FDG) PET, at 52 weeks in an Alzheimer disease–associated prespecified statistical region of interest. Secondary end points included change in cognition, function, and other biomarkers.

**Results:**

Among the 159 participants, 79 were randomized to receive AZD0530 and 80 to receive placebo. Of the 159 participants, 87 (54.7%) were male, with a mean (SD) age of 71.0 (7.7) years. Based on a week-2 plasma drug level (target = 180 ng/mL; 30nM free), 15 participants (19.2%) had their AZD0530 dose escalated from 100 mg to 125 mg. Mean plasma levels from weeks 13 to 52 were 220 ng/mL and 36nM free. More participants discontinued treatment with AZD0530 than with placebo (21 vs 11), most commonly because of adverse events. The most frequent adverse events were gastrointestinal disorders (primarily diarrhea), which occurred in 38 participants (48.1%) who received AZD0530 and in 23 (28.8%) who received placebo. In the primary outcome, the treatment groups did not differ in 52-week decline in relative CMRgl (mean difference: −0.006 units/y; 95% CI, −0.017 to 0.006; *P* = .34). The treatment groups also did not differ in the rate of change in Alzheimer’s Disease Assessment Scale–Cognitive Subscale, Alzheimer’s Disease Cooperative Study–Activities of Daily Living, Clinical Dementia Rating, Neuropsychiatric Inventory, or Mini-Mental State Examination scores. Secondary volumetric magnetic resonance imaging analyses revealed no treatment effect on total brain or ventricular volume but did show trends for slowing the reduction in hippocampal volume and entorhinal thickness.

**Conclusions and Relevance:**

Statistically significant effects of AZD0530 treatment were not found on relative CMRgl reduction in an Alzheimer disease–associated region of interest or on secondary clinical or biomarker measures.

**Trial Registration:**

ClinicalTrials.gov identifier: NCT02167256

## Key Points

**Question:**

Can fyn inhibition by AZD0530 slow the decline in relative cerebral metabolic rate for glucose and the change in secondary end points in cognition, function, and other biomarkers in participants with mild Alzheimer dementia?

**Findings:**

In this multicenter randomized clinical trial of 159 participants with mild Alzheimer dementia, AZD0530 treatment did not differ from placebo in slowing cerebral metabolic decline in an Alzheimer disease–associated prespecified statistical region of interest. Secondary end points revealed no treatment effects on the rate of change in cognition, function, and other biomarkers but revealed trends for slowing the decrease in hippocampal volume and entorhinal thickness.

**Meaning:**

Although this trial found no statistically significant effects of AZD0530 treatment on the relative cerebral metabolic rate for glucose or on secondary clinical or biomarker measures, it provides support for cerebral metabolic rate for glucose, as measured by ^18^F-fluorodeoxyglucose positron emission tomography, as a statistically powerful outcome measure that is well correlated with clinical outcomes.

## Teaser

This randomized clinical trial examines the effects, safety, and tolerability of AZD0530 medication in patients with mild Alzheimer disease.

## Introduction

In Alzheimer disease, the amyloid-β peptide (Aβ) accumulates in the brain as insoluble plaque and soluble oligomers (Aβo). The early accumulation of Aβ, in turn, triggers synaptic damage, inflammatory reaction, and pathological tau with cognitive impairment. Therapeutic development efforts have concentrated on limiting Aβ cleavage from amyloid precursor protein by secretase inhibition^1^ or on promoting its clearance by active or passive immunization.^2^ One alternative approach is to limit the toxic effects of accumulated Aβ rather than its level. Although Aβo can assume a range of different species, evidence has shown that multiple forms of Aβo are damaging, either directly or in concert with microglia.^3,4,5,6,7,8,9,10,11,12,13^

To interrupt Aβo-induced synaptic dysfunction, dendritic spine loss, inflammatory mediator recruitment, and memory dysfunction, an understanding of Aβo’s biochemical action is central. The only reported genome-wide expression screen for receptors has identified cellular prion protein as an oligomer-specific high-affinity binding site.^8,14^ Pathological signals from Aβo or cellular prion protein are transmitted through its coreceptor metabotropic glutamate receptor 5 to intracellular signaling.^15,16^ Critical for downstream signaling is the tyrosine kinase, fyn,^17,18^ which regulates the Alzheimer disease risk gene product PTK2B, the glutamate receptor subunit NR2B, and the neurofibrillary tangle–forming tau protein.^15,16,17,18,19,20,21,22,23,24,25,26,27,28,29,30,31^ Thus, fyn inhibition provides a potential target for disease-modifying therapy. AZD0530 (saracatinib) is a potent small-molecule inhibitor of Src family kinases.^32,33^ In transgenic mouse Alzheimer disease models, AZD0530 rescues deficits in synaptic density, learning and memory, and tau accumulation at a dose of 5 mg/kg/d but not 2 mg/kg/d.^27^

A previous phase 1b multiple ascending-dose study of AZD0530 in Alzheimer disease^34^ demonstrated the safety, tolerability, and central nervous system availability of oral AZD0530 for 4 weeks. Both the 100-mg and 125-mg doses achieved cerebrospinal fluid (CSF) drug levels similar to those that rescued memory deficits in transgenic mice.^27^

A major challenge in the development of treatments for Alzheimer disease is rapid and cost-effective evaluation.^34^ Owing to the high test-retest variability of clinical outcomes, researchers have sought biomarkers that reflect Alzheimer disease progression to assess disease-modifying treatments with greater statistical power.^35,36^ One biomarker of Alzheimer disease progression is the decline of regional cerebral metabolic rate for glucose (CMRgl) as measured by ^18^F-fluorodeoxyglucose (^18^F-FDG) positron emission tomography (PET).^34,37,38^ Chen et al^39^ have introduced an empirically defined statistical region of interest (consisting of voxels associated with preferential 12-month CMRgl declines relative to a spared region in an independent Alzheimer disease sample) to achieve optimal power.

Thus, the primary aims of this randomized clinical trial were to assess (1) the effect of AZD0530 treatment on 52-week reductions in relative CMRgl using ^18^F-FDG PET measurements in the predefined statistical region of interest and (2) the safety and tolerability of AZD0530 treatment over 52 weeks in participants with mild Alzheimer disease. This trial also acquired data for secondary clinical and biomarker end points.

## Methods

### Study Design and Participants

Recruitment for this phase 2a trial took place from December 23, 2014, to November 30, 2016. The last participant visit occurred on January 3, 2018, and final analyses were conducted from February 9, 2018, to July 25, 2018. Written informed consent was obtained from all participants in compliance with federal, state, and institutional review board requirements. This trial was registered at ClinicalTrials.gov (NCT02167256) and was approved by the institutional review boards of Yale University, the University of Southern California, and the 22 participating sites; the trial protocol is included in Supplement 1.

**Figure 1.**
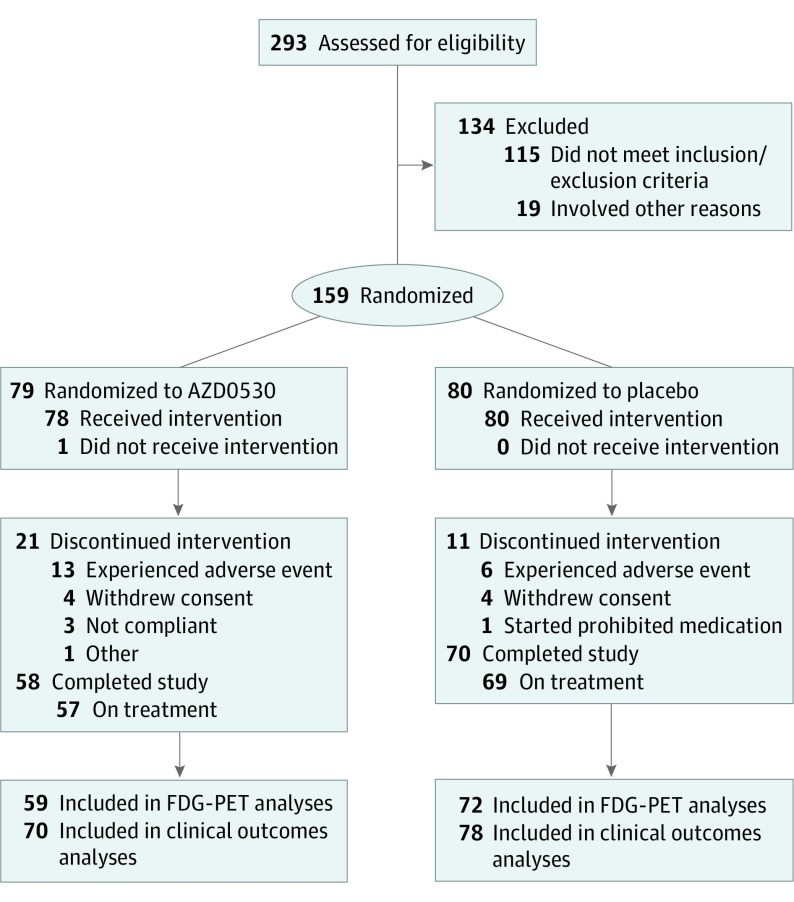
CONSORT Diagram

The primary enrollment criteria were a diagnosis of mild Alzheimer disease dementia as determined by the National Institute on Aging and Alzheimer’s Association core clinical criteria^40^ and evidence of Aβ pathogenesis based on central review of a ^18^F-florbetapir PET scan (eMethods in Supplement 2). Additional criteria included age 55 to 85 years and scores of 4 or lower on a modified Hachinski Ischemia Scale^41^ (score range: 0-12, with the highest score indicating highest probability of vascular dementia), 6 or lower on the Geriatric Depression Scale (score range: 0-15, with the highest score indicating most depressive symptoms),^42^ and 18 to 26 on the Mini-Mental State Examination (MMSE).^43^ In addition, cholinesterase inhibitors and memantine hydrochloride were permitted if stable for 12 weeks prior to screening. More complete exclusion criteria are provided in the eMethods in Supplement 2. Participants who met the eligibility requirements were randomized to receive either AZD0530 or placebo using a permuted block method stratified by site (Figure 1).

### Dosing Procedures

Study medication was taken in the morning with or without food. The AZD0530 treatment group initially received 100 mg daily. At the week 2 visit, total plasma AZD0530 levels were measured (Alzheimer Disease Cooperative Study [ADCS] Biomarker Core), and those participants in the active treatment group with sufficient compliance but with levels less than 100 ng/mL were given an increase at the week 4 visit to 125 mg daily for the remainder of the study. The control group received the placebo comparator for the entire study. The rationale for dose selection and the method of matching both doses of AZD0530 and placebo are provided in the eMethods in Supplement 2.

### Safety Assessments

After randomization, participants were evaluated at weeks 2, 4, 6, 8, 13, 19, 26, 32, 39, 45, and 52. Safety was assessed by reported adverse events, vital signs, and laboratory tests at all visits. Physical and neurological examinations, pharmacokinetics analysis of AZD0530, electrocardiography, and magnetic resonance imaging (MRI) scans were performed at selected visits. At higher doses in cancer studies, AZD0530 has been associated with neutropenia and thrombocytopenia.^44^ Therefore, laboratory criteria for considering drug discontinuation included an absolute neutrophil count of less than 1500/μL (to convert to ×10^9^/L, multiply by 0.001) or a platelet count of under 100 × 10^3^/μL (to convert to ×10^9^/L, multiply by 1.0). Previous experience with AZD0530 in patients with advanced solid tumors has also indicated a possible rare relationship with interstitial lung disease.^44^ For this reason, thoracic high-resolution computed tomography (CT) was obtained if unexplained pulmonary symptoms arose. All safety data were reviewed quarterly by the independent Data and Safety Monitoring Board.

### ^18^F-FDG PET Methods

The primary outcome was ^18^F-FDG PET measurement of the reduction in relative CMRgl using statistical parametric mapping of an Alzheimer disease–associated statistical region of interest, as described in previous studies.^39,45^ The ^18^F-FDG PET scans were acquired at baseline and week 52 by a standardized protocol. Participants were instructed to fast for 4 or more hours prior to scans. A 30-minute dynamic emission scan consisting of six 5-minute frames, either preceded by a CT scan (for PET/CT scanners) or followed by a transmission scan (for PET-only scanners), was acquired starting 30 minutes after intravenous injection of ^18^F-FDG (5 mCi) as the patient lay quietly in a dimly lit room. Data were corrected for radiation attenuation and scatter using transmission scans or x-ray CT and reconstructed using standardized algorithms.

### Clinical Assessments

The clinical effects of AZD0530 treatment were assessed by the Alzheimer’s Disease Assessment Scale-Cognitive Subscale (ADAS-Cog11; score range: 0-70, with the highest score indicating worst),^46,47^ MMSE,^43^ ADCS-Activities of Daily Living Scale (ADCS-ADL; score range: 0-78, with the highest score indicating best),^48^ Clinical Dementia Rating-Sum of Boxes (CDR-SB; score range: 0-18, with the highest score indicating worst),^49^ and Neuropsychiatric Inventory (NPI; score range: 0-144, with the highest score indicating worst).^50,51^ The ADAS-Cog11 was administered at baseline and weeks 13, 26, 39, and 52, and the MMSE was administered at screening and weeks 13, 26, 39, and 52. The ADCS-ADL, CDR-SB, and NPI were all administered at baseline and weeks 26 and 52.

### MRI Methods

Magnetic resonance imaging scans were acquired using a standard protocol (eMethods in Supplement 2) and were read locally to confirm eligibility. Magnetic resonance imaging was also performed at week 52 to assess treatment effects on the rate of change in total brain volume, ventricular volume, hippocampal volume, and entorhinal thickness. Measurement relied on nonlinear registration between baseline and follow-up images to calculate point-by-point volumetric change,^52^ along with FreeSurfer-based probabilistic-atlas image segmentation to calculate mean change across regions of interest as defined in the Desikan-Killiany atlas (eMethods in Supplement 2).^53^

### Cerebrospinal Fluid Analysis

Cerebrospinal fluid was obtained optionally in a subset of participants at baseline and week 52 to assess the effect of AZD0530 treatment on CSF total tau and pTau. Samples (≤20 mL) were collected after an 8-hour fast, and study medication was held on the morning of the procedure. A sample of 1 to 2 mL of CSF was sent to the local laboratory for protein, glucose, and cell count. The remaining CSF sample was shipped frozen to the ADCS Biomarker Core for processing and analysis. Levels of AZD0530 in CSF were also obtained at week 52.

### Statistical Analysis

Prospective power was based on pilot estimates for a mean (SD) 12-month reduction in CMRgl, as measured by ^18^F-FDG PET, of 0.0514 (0.0309)^39^ in the control arm. Assuming an attrition rate of 10%, we required a sample of 152 participants to detect a 30% effect of AZD0530 with 80% power at a 2-tailed α level of .05.

Demographic and baseline characteristics of the 2 treatment groups were compared using the Fisher exact test for categorical variables and 2-sample *t* test for continuous variables. Efficacy analyses of all primary and secondary outcomes were conducted in a modified intention-to-treat population, namely, all randomized participants who had at least 1 postbaseline assessment. Clinical outcomes with missing item scores were imputed using a proration strategy as detailed in the eMethods in Supplement 2. We used a serial gatekeeping procedure to maintain an overall experimentwise type I error rate of 5% for 6 outcome hypotheses (^18^F-FDG PET–measured CMRgl, ADAS-Cog11, ADCS-ADL, CDR-SB, MMSE, and NPI).

Because ^18^F-FDG PET images were collected at 2 time points, a linear mixed-effects regression model was used to compare rates of change between treatment groups, assuming a common mean CMRgl at baseline. This model included fixed effects for time from randomization (continuous), age at baseline, and apolipoprotein E (*APOE*) ε4 carrier status as well as participant-specific random intercepts. This model was also used for prespecified post hoc subgroup analyses based on compliance (80%-120% by pill counts), ^18^F-florbetapir PET standardized uptake value ratio (by quartiles), and screening MMSE (median split).

The mixed model of repeated measures was used for all secondary outcome measures assessed at more than 2 time points. The dependent variable of the mixed model of repeated measures was the change from baseline at each follow-up visit. The model treated time as a categorical variable and included fixed effects for the treatment-by-time interactions, baseline outcome, age, and *APOE* ε4 status. An unstructured correlation and heterogeneous variance with respect to time was assumed.

Safety analyses were conducted on the intention-to-treat population, namely, all randomized participants. The Fisher exact test was used to compare frequencies of adverse events or laboratory abnormalities between treatment groups. Population pharmacokinetics analysis of concentration-time data of AZD0530 was also performed using the mixed model of repeated measures. Magnetic resonance imaging and CSF biomarker outcomes were analyzed using analysis of covariance, including mean baseline value, age, and *APOE* ε4 status as covariates. All statistical analyses were performed with R, version 3.4.2 (R Foundation for Statistical Computing), and results are reported as point estimates with 95% CIs. A 2-sided *P* = .05 was considered statistically significant.

As detailed in the eMethods in Supplement 2, the statistical analysis plan changed from the original to the final protocol (Supplement 1). However, these changes did not alter the final results.

## Results

As shown in Figure 1, a total of 293 participants were screened for this trial, and 159 were randomized: 79 were randomized to the AZD0530 group (1 of whom never received the drug) and 80 to the placebo group. All 159 participants were included in the intention-to-treat population for safety analyses, and 128 (80.5%) completed the study (126 of whom were receiving treatment). Early treatment discontinuations, primarily owing to adverse events, included 21 (26.6%) in the AZD0530 group and 11 (13.8%) in the placebo group.

### Baseline Characteristics

Participant baseline characteristics are displayed in Table 1. Of the 159 randomized participants, 87 (54.7%) were male, with a mean (SD) age of 71.0 (7.7) years, and 105 (66.0%) were *APOE* ε4 carriers. The mean (SD) MMSE score was 22.5 (2.5). Baseline characteristics were generally well balanced between treatment groups. However, the mean (SD) baseline NPI score was higher in the AZD0530 group compared with the placebo group (11.6 [13.2] vs 7.5 [8.1]; *P* = .05).

**Table 1.** Baseline Participant Characteristics

Variable	AZD0530 Group	Placebo Group	Combined Group	*P* Value^a^
Total No.	79	80	159	
Sex, No. (%).				.11
Male	38 (48.1)	49 (61.2)	87 (54.7)	
Female	41 (51.9)	31 (38.7)	72 (45.3)	
Race/ethnicity, No. (%)				.16
White (not Hispanic)	74 (93.7)	68 (85.0)	142 (89.3)	
Black or African American	4 (5.1)	3 (3.7)	7 (4.4)	
Hispanic or Latino	1 (1.3)	6 (7.5)	7 (4.4)	
American Indian or Alaskan Native	0	1 (1.2)	1 (0.6)	
>1 Race	0	1 (1.2)	1 (0.6)	
Unknown or not reported	0	1 (1.2)	1 (0.6)	
*APOE* ε4, No. (%)				.51
No	29 (36.7)	25 (31.2)	54 (33.9)	
Yes	50 (63.3)	55 (68.7)	105 (66.0)	
Age, mean (SD), y	70.9 (8.0)	71.2 (7.4)	71.0 (7.7)	.85
Education, mean (SD), y	16.01 (2.84)	16.14 (2.94)	16.08 (2.88)	.86
Baseline ADAS-Cog11 score, mean (SD)	21.35 (8.42)	21.04 (7.09)	21.19 (7.76)	.79
Baseline ADCS-ADL score, mean (SD)	65.80 (8.31)	66.83 (8.47)	66.31 (8.38)	.52
Baseline CDR-SB score, mean (SD)	5.15 (2.28)	5.06 (2.17)	5.11 (2.22)	.81
Screening MMSE score, mean (SD)	22.62 (2.47)	22.32 (2.44)	22.47 (2.45)	.52
Baseline NPI score, mean (SD)	11.62 (13.22)	7.50 (8.09)	9.55 (11.10)	.051^b^
Screening GDS score, mean (SD)	1.54 (1.59)	1.79 (1.51)	1.67 (1.55)	.18
Screening modified Hachinski Ischemia Scale score, mean (SD)^c^	0.633 (0.880)	0.688 (0.773)	0.660 (0.825)	.36

Abbreviations: ADAS-Cog11, Alzheimer’s Disease Assessment Scale–Cognitive Subscale (score range: 0-70, with the highest score indicating worst); ADCS-ADL, Alzheimer’s Disease Cooperative Study–Activities of Daily Living (score range: 0-78, with the highest score indicating best); *APOE*, apolipoprotein E; CDR-SB, Clinical Dementia Rating–Sum of Boxes (score range: 0-18, with the highest score indicating worst); GDS, Geriatric Depression Scale (score range: 0-15, with the highest score indicating most depressive symptoms); MMSE, Mini-Mental State Examination; NPI, Neuropsychiatric Inventory (score range: 0-144, with the highest score indicating worst).

^a^ Fisher exact test was used for categorical variables, and 2-sample *t* test was used for continuous variables.

^b^ Group NPI differences were driven primarily by differences in mean (SD) Anxiety (1.6 [2.9] vs 0.7 [1.3]; *P* = .03) and Agitation (0.9 [1.5] vs 0.6 [1.4]; *P* = .06) Wilcoxon rank sum test scores between the AZD0530 and placebo groups.

^c^ Modified Hachinski Ischemia Scale score range: 0-12, with the highest score indicating highest probability of vascular dementia.

### Safety and Tolerability

The number of participants in each treatment group who experienced adverse events is presented in Table 2. In general, 100 mg to 125 mg daily of AZD0530 was reasonably well tolerated. A total of 593 adverse events were reported (of which 389 were mild, 176 moderate, and 28 severe; 331 of these adverse events were in the AZD0530 group, and 262 in the placebo group). Seventy-three participants (92.4%) receiving AZD0530 and 65 (81.2%) receiving placebo experienced at least 1 adverse event during the study (*P* = .06, Fisher exact test). The most frequent adverse events were gastrointestinal, which occurred in 38 participants (48.1%) receiving AZD0530 and 23 (28.7%) receiving placebo (*P* = .02, Fisher exact test). These gastrointestinal disorders were primarily driven by diarrhea, the most common individual adverse event, which occurred in 22 participants (27.8%) receiving AZD0530 and 9 (11.2%) receiving placebo. Risk differences in adverse events by MedDRA (Medical Dictionary for Regulatory Activities) System Organ Class between participants in the AZD0530 and placebo groups are graphically displayed in eFigure 1 in Supplement 2.

**Table 2.** Reported Adverse Events

Adverse Event	AZD0530 Group (n = 79)	Placebo Group (n = 80)	Total (n = 159)	*P* Value^a^
No. (%) of participants with ≥1 event				
Adverse event	73 (92.4)	65 (81.2)	138 (86.8)	.06
Serious adverse event	12 (15.2)	7 (8.7)	19 (11.9)	.23
No. of participants (%) with ≥1 adverse event by MedDRA System Organ Class^b^				
Gastrointestinal disorders	38 (48.1)	23 (28.8)	61 (38.4)	.02
Infections and infestations	28 (35.4)	24 (30.0)	52 (32.7)	.50
Psychiatric disorders	27 (34.2)	17 (21.2)	44 (27.7)	.08
Investigations	24 (30.4)	17 (21.2)	41 (25.8)	.21
Nervous system disorders	18 (22.8)	16 (20.0)	34 (21.4)	.70
Skin and subcutaneous tissue disorders	18 (22.8)	10 (12.5)	28 (17.6)	.10
General disorders and administration site conditions	15 (19.0)	9 (11.2)	24 (15.1)	.19
Musculoskeletal and connective tissue disorders	14 (17.7)	24 (30.0)	38 (23.9)	.09
Injury, poisoning, and procedural complications	13 (16.5)	22 (27.5)	35 (22.0)	.13
Respiratory, thoracic, and mediastinal disorders	13 (16.5)	7 (8.7)	20 (12.6)	.16
Metabolism and nutrition disorders	11 (13.9)	3 (3.7)	14 (8.8)	.03
Renal and urinary disorders	6 (7.6)	2 (2.5)	8 (5.0)	.17
Eye disorders	5 (6.3)	1 (1.2)	6 (3.8)	.12
Blood and lymphatic system disorders	4 (5.1)	2 (2.5)	6 (3.8)	.44
Cardiac disorders	3 (3.8)	6 (7.5)	9 (5.7)	.50
Ear and labyrinth disorders	3 (3.8)	1 (1.2)	4 (2.5)	.37
Benign, malignant, and unspecified neoplasms (including cysts and polyps)	3 (3.8)	8 (10.0)	11 (6.9)	.21
Reproductive system and breast disorders	3 (3.8)	1 (1.2)	4 (2.5)	.37
Vascular disorders	3 (3.8)	6 (7.5)	9 (5.7)	.50
Immune system disorders	2 (2.5)	1 (1.2)	3 (1.9)	.62
Endocrine disorders	1 (1.3)	0 (0)	1 (0.6)	.50

Abbreviation: MedDRA, Medical Dictionary for Regulatory Activities.

^a^ Fisher exact test; unadjusted for multiple comparisons.

^b^ Sorted by adverse event rate in the AZD0530 group.

A total of 24 serious adverse events were reported during the study, with 16 among participants receiving AZD0530 and 8 among participants receiving the placebo. Two adverse events were deemed by site investigators as possibly related to the study drug: delirium (placebo group) and acute diverticulitis (AZD0530 group). One death (owing to urinary tract infection) was reported in the active treatment group and deemed unrelated to the study drug. Among the participants who met the protocol-specified discontinuation criteria, 2 (1 from the AZD0530 group, and 1 from the placebo group) discontinued the study drug owing to neutropenia, but 0 discontinued for thrombocytopenia or interstitial lung disease.

### Dosing and Pharmacokinetics

Of the 78 participants who received active AZD0530, 15 (19.2%) had their dose escalated from 100 mg to 125 mg at week 4 based on week 2 plasma levels. The mean plasma levels from weeks 13 to 52 were 220 ng/mL and 36nM free (target = 180 ng/mL; 30nM free). Only 13 participants had a week-52 lumbar puncture while receiving active treatment, with a mean (SD) CSF AZD0530 level of 2.3 (1.3) ng/mL (4.3 [2.5]nM). From the previous phase 1b study, we targeted CSF levels of 5nM^54^ or greater and predicted levels of 4.5 ng/mL (8nM), which was within the range of the fyn Ki (inhibition constant) for AZD0530 (5-10nM) and the efficacious levels in Alzheimer disease model mice (5.8-14nM).^27^

### Primary Efficacy Measure: ^18^F-FDG PET

**Figure 2.**
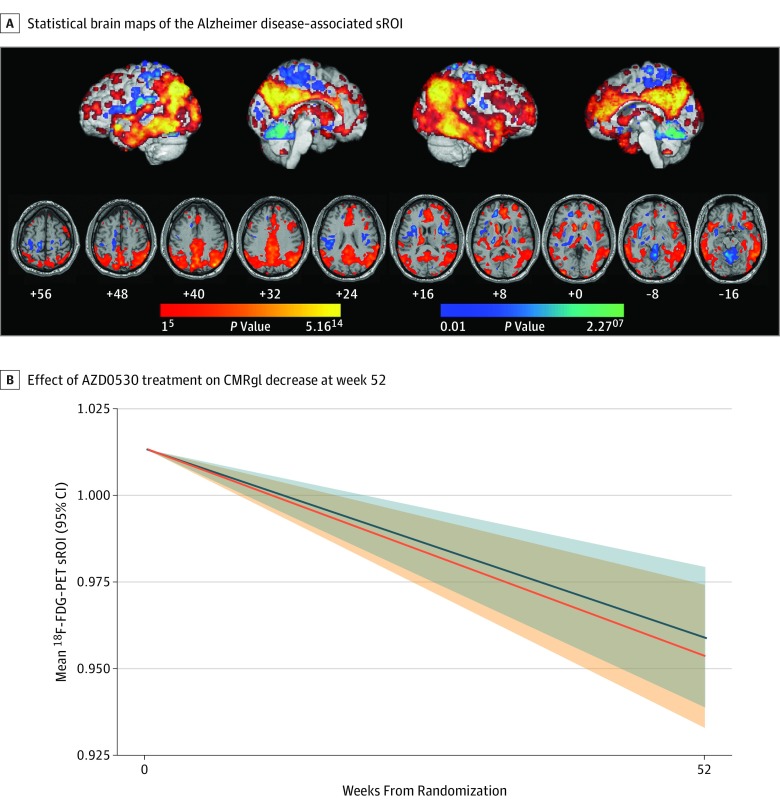
Primary Outcome A, A 52-week cerebral metabolic rate for glucose (CMRgl) decline statistical region of interest (sROI; in the red-to-yellow color scale) and a spared sROI (in the blue-to-green color scale) were generated using baseline and follow-up. Shown are ^18^F-fluorodeoxyglucose (^18^F-FDG) positron emission tomography (PET) images acquired in an Alzheimer’s Disease Neuroimaging Initiative study and updated for amyloid-positive participants with Alzheimer disease, as previously described.^39,45^ B. The y-axis represents relative CMRgl-derived ^18^F-FDG PET in the Alzheimer disease–associated sROI normalized to the spared sROI. The treatment groups did not differ in the 52-week decline in CMRgl (difference, −0.006 units/y; 95% CI, −0.017 to 0.006; *P* = .34). The mean (SD) 12-month decrease in ^18^F-FDG PET CMRgl was .0525 (.0340) in the placebo (control) group (n = 72), which was close to the pilot estimates used in the power analysis, and 0.0569 (0.0303) in the AZD0530 group (n = 59). Blue represents the placebo group; orange, the AZD0530 group.

The primary outcome was ^18^F-FDG PET measurement of a decline in relative CMRgl at week 52 in an Alzheimer disease–associated statistical region of interest^39,45^ (Figure 2). No statistically significant difference was observed between the AZD0530 and placebo groups (difference: −0.006 units/y; 95% CI, −0.017 to 0.006; *P* = .34). One hundred thirty-one participants (59 in the AZD0530 group and 72 in the placebo group) received both baseline and follow-up ^18^F-FDG PET. Prespecified subgroup analyses based on compliance, ^18^F-florbetapir PET standardized uptake value ratio (by quartiles), and screening MMSE (median split) were consistent with the primary analysis. An additional exploratory subgroup analysis based on baseline CMRgl (median split) suggested that treatment differences favored placebo above the median CMRgl (difference: –0.027 units/y; 95% CI, –0.043 to –0.010; *P* = .002) but favored AZD0530 below the median (difference: 0.014 units/y; 95% CI, –0.00002 to 0.027; *P* = .05). Relative CMRgl as the primary outcome was well correlated with standard clinical outcomes both cross-sectionally and long term (eFigure 2 in Supplement 2) and demonstrated greater precision (narrower CIs) compared with any clinical measure (eFigure 3 in Supplement 2).

### Secondary Outcomes

**Figure 3.**
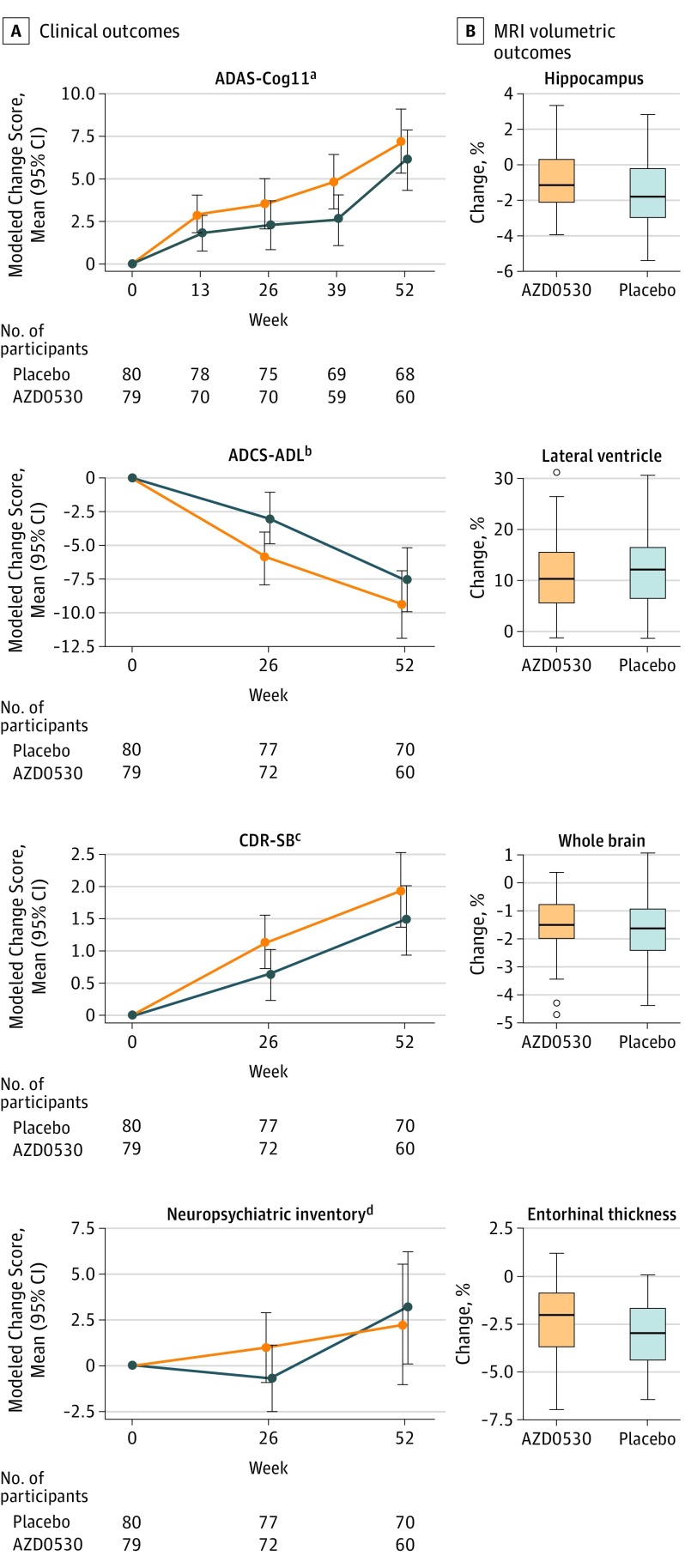
Secondary Outcomes A, Analyses of clinical variables used a mixed model of repeated measures to estimate the mean group difference at each follow-up time, with change from baseline as the outcome, controlling for baseline score, age, and apolipoprotein E (*APOE*) ε4 status. B. Analyses of magnetic resonance imaging (MRI) variables used an analysis of covariance model with percentage of deformation per year from baseline as the outcome, adjusted for mean baseline volume or thickness of brain region, age, and *APOE* ε4 status. Blue represents the placebo group; orange, the AZD0530 group. ^a^Alzheimer’s Disease Assessment Scale–Cognitive Subscale (ADAS-Cog11) score range: 0 (indicating best) to 70 (worst). ^b^Alzheimer’s Disease Cooperative Study–Activities of Daily Living (ADCS-ADL) score range: 0 (worst) to 78 (best). ^c^Clinical Dementia Rating–Sum of Boxes (CDR-SB) score range: 0 (best) to 18 (worst). ^d^Neuropsychiatric Inventory score range: 0 (best) to 144 (worst).

Results for secondary clinical outcomes are summarized in Figure 3A. No statistically significant treatment effects were observed for any outcome. For the ADAS-Cog11, the AZD0530 (treatment) group score increased by 7.26 (95% CI, 5.39-9.14) compared with the placebo (control) group score (6.14; 95% CI, 4.36-7.91; *P* = .39). For the ADCS-ADL, the AZD0530 group score decreased by 9.49 (95% CI, 7.00-11.97) compared with the placebo group score (7.64; 95% CI, 5.28-10.00; *P* = .29). For the CDR-SB, the AZD0530 group score increased by 1.95 (95% CI, 1.37-2.52) compared with the placebo group score (1.47; 95% CI, 0.93-2.01; *P* = .23). For the NPI, the AZD0530 group score increased by 2.24 (95% CI, 1.08-5.56) compared with the placebo group score (3.16; 95% CI, 0.71-6.24; *P* = .69). For the MMSE, the AZD0530 group score decreased by 3.84 (95% CI, 2.71-4.97) compared with the placebo group score (3.33; 95% CI, 2.26-4.39; *P* = .51).

Results for MRI volumetric outcomes are summarized in Figure 3B. For hippocampal volume, the mean (SD) volume of the AZD0530 group (n = 57) decreased by 0.89% (1.81%) compared with the placebo group (n = 62) volume decrease (1.54% [1.99%]; *P* = .09). For lateral ventricular volume, the mean (SD) volume of the AZD0530 group (n = 57) increased by 11.35% (7.09%) compared with the placebo group (n = 62) volume increase (11.67% [6.45%]; *P* = .85). For whole-brain volume, the mean (SD) volume of the AZD0530 group (n = 57) decreased by 1.60% (1.06%) compared with the placebo group (n = 62) volume decrease (1.71% [1.09%]; *P* = .98). For entorhinal cortical thickness, the mean (SD) volume of the AZD0530 group (n = 57) decreased by 2.39% (1.81%) compared with the placebo group (n = 62) volume decrease (3.10 [1.74%]; *P* = .07). Changes in hippocampal volume and entorhinal thickness were not correlated with changes in clinical outcomes (ADAS-Cog11, MMSE, CDR-SB, or NPI) in the overall sample. In light of the trends for slowing of decline by AZD0530 in hippocampal volume and entorhinal thickness, we conducted post hoc exploratory analyses of ^18^F-FDG PET measurement of decline in relative CMRgl in the hippocampus and entorhinal cortex (with global normalization). We observed a slowing of decline in the entorhinal cortex (difference: 0.014 units/y; 95% CI, –0.00052 to 0.027; *P* = .04) but not in the hippocampus (difference: 0.00016 units/y; 95% CI, −0.017 to 0.018; *P* = .99).

The CSF substudy included 53 participants at baseline, 36 at week 52, and 34 at both time points, enabling the calculation of the rates of change in Alzheimer disease biomarkers (eFigure 4 in Supplement 2). No statistically significant treatment differences were observed for rates of change in either CSF total tau (difference: 98.3 pg/mL/y; 95% CI, −24.9 to 221.4; *P* = .11) or pTau (difference: 3.65 pg/mL/y; 95% CI, −7.55 to 14.84; *P* = .51).

## Discussion

This phase 2a randomized clinical trial demonstrated that a 100-mg to 125-mg daily dose of AZD0530 is reasonably safe and well tolerated in participants with mild Alzheimer disease. However, in comparison to placebo, AZD0530 treatment had no significant effect on ^18^F-FDG PET–measured reduction in relative CMRgl at 52 weeks in an Alzheimer disease–associated statistical region of interest. The treatment groups also did not significantly differ in secondary clinical outcomes, including rates of change in ADAS-Cog11, ADCS-ADL, CDR-SB, NPI, or MMSE scores.

Secondary MRI analyses revealed no statistically significant treatment effects on any of 4 volumetric measures but did show trends for slowing the decline in hippocampal volume and entorhinal thickness. Additional credence was lent to these trends by post hoc exploratory analyses of ^18^F-FDG PET–measured reduction in relative CMRgl for entorhinal cortex but not for hippocampus. Although AZD0530 demonstrated no treatment effect on neuroimaging outcomes in this study, we cannot exclude the possibility of some regionally specific effects on brain structure and function. A previous study has shown in Alzheimer disease model mice that chronic fyn inhibition with AZD0530 treatment restores memory function and markers of synaptic density (PSD-95 and SV2a) in the dentate gyrus of the hippocampus induced by APP/PS1 transgenes.^27^ AZD0530 treatment may have a more focal effect on medial temporal lobe structure and function.

Although disappointing, these results do not exclude fyn kinase as a therapeutic target in Alzheimer disease. Our previous findings in Alzheimer disease model mice may not have translated into mild Alzheimer disease dementia because of inadequate study drug dose and limited inhibition of fyn in the brain. Overall, the targeted plasma levels (180 ng/mL; 30nM free) were achieved in the present trial. Mean plasma levels from weeks 13 to 52 were 220 ng/mL and 36nM free. However, in a small CSF pharmacokinetic substudy, drug levels fell below the targets suggested by the previous mouse study^27^ and phase 1b trial.^54^ Preclinical dose reduction from 5 mg/kg/d to 2 mg/kg/d eliminated the efficacy in transgenic mice. The tolerability of a daily dose of 100 mg to 125 mg of AZD0530 in the current study suggests that higher doses may be unfeasible in the Alzheimer disease population such that a narrow therapeutic window in mice is closed for human participants.

Numerically, more participants discontinued treatment with AZD0530 than with placebo (21 vs 11), primarily owing to adverse events. The most frequent adverse events were diarrhea and other gastrointestinal disorders, which were significantly more common in the AZD0530 treatment arm. Nonetheless, selective fyn inhibitors might be developed that would have greater tolerability to permit more complete target engagement. Alternatively, higher AZD0530 doses in those individuals who can tolerate such a regimen, and perhaps who have the greatest ^18^F-FDG hypometabolism, might be effective in limiting cognitive decline. Further optimization of fyn inhibition is required to fully evaluate the enzyme as a target for disease modification in Alzheimer disease.

The results of this trial provide strong support for the use of CMRgl, measured by ^18^F-FDG PET, as a primary outcome in a proof-of-concept study. ^18^F-FDG PET demonstrated the clinical relevance of CMRgl as a biomarker outcome in that it was well correlated with cognitive and functional outcomes both cross-sectionally and longitudinally (eFigure 2 in Supplement 2). Findings are consistent with longitudinal associations between ^18^F-FDG PET and clinical measures in previous observational studies^36^ in the context of a therapeutic trial. Additional studies showing an association between an effective treatment’s ^18^F-FDG PET and clinical findings are needed to provide further support for its theragnostic value. Moreover, relative CMRgl in an Alzheimer disease–associated statistical region of interest proved to be a statistically powerful biomarker measure with at least twice the precision of the best clinical measures, demonstrating that it would have power to detect active placebo differences that are less than half as great as for clinical measures (eFigure 3 in Supplement 2). This trial also supports the feasibility of another novel element: the use of early drug-level monitoring to adjust the final dose. Week 2 plasma drug levels were measured by a central laboratory with rapid turnaround to guide potential double-blind dose escalation in the active treatment arm at week 4.

### Limitations

This study has a number of limitations. First, the larger-than-expected rate of attrition diminished the statistical power of the study to detect all but a large (30%) effect size. Second, the availability of CSF in only 21% of trial participants limited our ability to evaluate the treatment effects on rates of change in CSF total tau or pTau or to assess the adequacy of doses in relation to CSF drug levels.

## Conclusions

In this 52-week randomized clinical trial, we could not detect statistically significant effects of AZD0530 treatment on relative CMRgl decline in an Alzheimer disease–associated region of interest or in secondary clinical or biomarker measures. However, this trial supports the use of CMRgl, as measured by ^18^F-FDG PET, as a statistically powerful outcome measure that is well correlated with clinical outcomes.

## Notes

Supplement 1. Trial Protocol

Supplement 2. **eMethods** **eFigure 1.** Proportion of Participants Reporting At Least One Adverse Event **eFigure 2.** Correlation Between ^18^F-FDG-PET and Clinical Assessments **eFigure 3.** Forest Plot of Effects for Primary and Secondary Outcomes **eFigure 4.** Secondary CSF Outcomes

Supplement 3. Data Sharing Statement
