# Supplementary material for: Effect of AZD0530 on Cerebral Metabolic Decline in Alzheimer Disease: A Randomized Clinical Trial
Source: JAMA Neurol. 2019 Jul 22;76(10):1219–29. doi: 10.1001/jamaneurol.2019.2050 (PMC6646979; doi:10.1001/jamaneurol.2019.2050)
Supplement: Supplement 1. — Trial Protocol [file jamaneurol-76-1219-s001.pdf]

A Phase 2a Multi-Center Study of ^{18}F-FDG PET, Safety, and Tolerability of AZD0530 in Mild Alzheimer's Disease

Protocol Short Title: FYN

Protocol Number: ADC-049

US IND Number: 118607

PROJECT DIRECTOR	COORDINATION & DATA MANAGEMENT CENTER
**Christopher van Dyck, M.D.**<br>Yale University<br>1 Church St. Suite 600<br>New Haven, CT 06510<br>Christopher.vandyck@yale.edu<br>Tel: (203) 764-8100	**Paul Aisen, M.D.**<br>Alzheimer's Therapeutic Research Institute (ATRI)<br>9860 Mesa Rim Road<br>San Diego, CA 92121<br>paisen@usc.edu<br>Tel: (858) 252-8555
**MEDICAL MONITOR**	**PET CENTRAL READ**
**Tiffany Chow, M.D.**<br>Alzheimer's Therapeutic Research Institute (ATRI)<br>9860 Mesa Rim Road<br>San Diego, CA 92121<br>Tel: 858-964-0462	**Kewei Chen, Ph.D.**<br>Director of Core Resources<br>Neuroimaging Research and Analysis Lab<br>Banner Alzheimer's institute<br>901 E. Willetta Street<br>Phoenix, AZ 85006<br>Kewei.Chen@bannerhealth.com<br>Tel: 602-839-4851
**PROTOCOL VERSION**	**VERSION DATE**
Protocol v1.0	19-NOV-2014
Protocol v2.0	10-AUG-2015
Protocol v3.0	31-MAY-2016
**Protocol v4.0**	**31-MAY-2017**

Confidentiality Statement

The information in this document is privileged or confidential and may not be disclosed unless such disclosure is required by applicable laws and regulations. It is the property of ATRI and Yale University and should not be copied by or distributed to persons not involved in the study. Persons to whom the information is disclosed must be informed that the information is privileged or confidential and may not be further disclosed by them. These restrictions on disclosure will apply equally to all future information supplied to you, which is indicated as privileged or confidential. Any unauthorized review, use, disclosure, copying or distribution is strictly prohibited.

Table of Contents

List of Abbreviations 4

PROTOCOL SYNOPSIS 7

1.0 INTRODUCTION 8
- 1.1 Primary Aims 9
- 1.2 Secondary Aims 9

2.0 PRELIMINARY STUDIES 9
- 2.1 Summary of Pre-Clinical Findings 9
- 2.2 Summary of Clinical Findings 14
- 2.3 Safety and Compliance 17

3.0 BACKGROUND AND SIGNIFICANCE 18
- 3.1 Rationale for Study 18
- 3.2 Rationale for Dosage Selection 20
- 3.3 Rationale for ^{18}F-Florbetapir PET and Biomarkers 20

4.0 POTENTIAL RISKS AND BENEFITS ASSOCIATED WITH THIS INVESTIGATIONAL PRODUCT 21
- 4.1 Potential Risks and Benefits Associated with This Investigational Product 21
- 4.2 Relationship of this intervention to current treatment practices in AD 21

5.0 SAMPLE SIZE AND STATISTICAL PLAN 22
- 5.1 Randomization 22
- 5.2 Power and Sample Size Determination 22
- 5.3 Selection of participants to be used in analysis 22
- 5.4 Efficacy Analysis 23
- 5.5 Interim Analysis 24
- 5.6 Criteria for the termination of the trial 25

6.0 STUDY DRUG AND CONCOMITANT MEDICATIONS 26
- 6.1 Name and Description of IP and Comparator 26
- 6.2 Dosage 26
- 6.3 Blinding 26
- 6.4 Packaging/Dispensing/Labeling 27
- 6.5 Storage 27
- 6.6 Drug Accountability 27
- 6.7 Compliance 27
- 6.8 Breaking the Blind 28
- 6.9 Concomitant AD Medications 28
- 6.10 Other Concomitant Medications 28

7.0 STUDY POPULATION 30
- 7.1 Inclusion Criteria 30
- 7.2 Exclusion Criteria 30
- 7.3 Recruitment and Retention Strategies 32

8.0 STUDY TIMELINE 32

9.0 DESCRIPTION OF STUDY VISITS 33
- 9.1 Pre-screening 33

9.2 Screening 33
9.3 Baseline Visit (Week 0) 34
9.4 Treatment Visits (Week 2 – Week 52) 34

10.0 EARLY TREATMENT/STUDY DISCONTINUATION 35
10.1 Reasons for Early Discontinuation 35

11.0 INSTRUMENTS 37
11.1 Cognitive Evaluations 37
11.2 Clinical and Functional Evaluations 38

12.0 PROCEDURES 39
12.1 Safety Assessments 39
12.2 Biofluids 40
12.3 Magnetic Resonance Imaging (MRI) 42
12.4 PET Imaging 43

13.0 PERSONNEL REQUIREMENTS 44

14.0 ADVERSE EVENTS 45
14.1 Definition 45
14.2 Following Up on AEs 45

15.0 SERIOUS ADVERSE EVENTS (SAE) 46
15.1 Definition 46
15.2 Reporting SAEs 46

16.0 DATA AND SAFETY MONITORING BOARD (DSMB) 47

17.0 RECORDING AND COLLECTION OF DATA 48
17.1 Case Report Form 48
17.2 Study Files and Patient Source Documents 48

18.0 ETHICS AND REGULATORY CONSIDERATIONS 49
18.1 Good Clinical Practice 49
18.2 Institutional Review Board (IRB) / Research Ethics Boards (REB) 49
18.3 Informed Consent and HIPAA Compliance 50
18.4 Genetic Research and Storage of Genetic Material 50
18.5 Storage of Biospecimen Samples 51
18.6 MRI and PET Imaging and Data Storage 51
18.7 Inclusion of Children as Participants 51
18.8 Study Monitoring 51

19.0 AUDIT 52

20.0 RECORD RETENTION 52

21.0 PUBLICATION POLICY 52

22.0 SHARING OF FINAL RESEARCH DATA 52

23.0 LITERATURE CITED 53

APPENDIX 1 SCHEDULE OF EVENTS 59

APPENDIX 2 REVISION HISTORY 60

List of Abbreviations

AD	Alzheimer's Disease
ADAS-Cog	Alzheimer's Disease Assessment Scale – Cognitive
ADC	Alzheimer's Disease Center
ADCS	Alzheimer's Disease Cooperative Study
ADCS-ADL	Alzheimer's Disease Cooperative Study - Activities of Daily Living Inventory
ADEAR	Alzheimer's Disease Education & Referral Center, under the NIA
ADNI	Alzheimer's Disease Neuroimaging Initiative
AE	Adverse Event
ALT	Alanine aminotransferase
APOE/APOE4	Apolipoprotein E (APOE) epsilon 4 (APOE4)
AST	Aspartate aminotransferase
ATRI	Alzheimer's Therapeutic Research Institute
Aβ	Beta Amyloid
CDR	Clinical Dementia Rating
CDR-SB	Clinical Dementia Rating, Sum of Boxes
CSF	Cerebrospinal Fluid
CT	Computerized Tomography
CTCAE	Common Terminology Criteria for Adverse Events
DNA	Deoxyribonucleic Acid
DSMB	Data Safety Monitoring Board
DSM	Diagnostic and Statistical Manual of Mental Disorders
ECG	Electrocardiogram
eCRF	Electronic Case Report Form
EDC	Electronic Data Capture System
FDG	Fluoro Deoxy Glucose
FLAIR	Fluid Attenuation Inversion Recovery
GCP	Good Clinical Practice
GDS	Geriatric Depression Scale
HIPAA	Health Insurance Portability and Accountability Act
ICH	International Conference on Harmonization
IRB	Institutional Review Board
LAR	Legally Authorized Representative
LDH	Lactate dehydrogenase
LP	Lumbar Puncture
MCI	Multiple Cerebral Infarctions

MMSE	Mini-Mental State Examination
MPRAGE	Magnetization Prepared Rapid Gradient Echo
MR/MRI	Magnetic Resonance / Magnetic Resonance Imaging
NCATS	National Center for Advancing Translational Sciences
NIA	National Institute on Aging, under the NIH
NIH	National Institutes of Health
NPI	Neuropsychiatric Inventory
PET	Positron-Emission Tomography
PI	Principal Investigator
PK	Pharmacokinetic
REB	Research Ethics Board
RSS	Research Satisfaction Survey
SAE	Serious Adverse Event
T	Tesla
TFT's	Thyroid Function Tests
TSH	Thyroid Stimulating Hormone
vMRI	Volumetric Magnetic Resonance Imaging

SIGNATURE PAGE

(SIGNATURES ON FILE AT COORDINATING CENTER)

______________________________	________________
Christopher van Dyck, M.D.<br>Project Director<br>Yale University	Date (MM/DD/YYYY)
______________________________	________________
Tiffany Chow, M.D.<br>Medical Monitor<br>Alzheimer's Therapeutic Research Institute	Date (MM/DD/YYYY)
______________________________	________________
Paul Aisen, M.D.<br>Director<br>Coordination & Data Management Center<br>Alzheimer's Therapeutic Research Institute	Date (MM/DD/YYYY)

PROTOCOL SYNOPSIS

**PROTOCOL TITLE**	A Phase 2a Multi-Center Study of ^{18}F-FDG PET, Safety, and Tolerability of AZD0530 in Mild Alzheimer's Disease
**PROJECT DIRECTOR**	Christopher van Dyck, M.D.
**STUDY SPONSOR**	National Center for Advancing Translational Sciences (NCATS), through a grant to Yale University
**COORDINATING CENTER**	Alzheimer's Therapeutic Research Institute (ATRI)
**STUDY DESIGN**	A Phase 2a multi-center, randomized, double-blind, placebo-controlled study evaluating ^{18}F-FDG PET, Safety, and Tolerability of AZD0530 in 152 patients with Mild AD
**DURATION OF STUDY PARTICIPATION**	Each subject will have up to a 60-day screening period, followed by 52 weeks of treatment.
**SUMMARY OF INVESTIGATIONAL PRODUCT**	AZD0530 100mg or 125mg, or Placebo tablets taken orally once daily
**SUMMARY OF KEY ELIGIBILITY CRITERIA**	• Diagnosis of probable AD (NIA-AA criteria)<br>• ^{18}F-Florbetapir scan with evidence of elevated Aβ (based on central review)<br>• Age 55-85 (inclusive)<br>• MMSE 18-26 (inclusive)<br>• Modified Hachinski Scale Score <=4
**PRIMARY OUTCOME MEASURE**	Aim 1. ^{18}F-FDG PET measurements of the cerebral metabolic rate for glucose (CMRgl)<br>Aim 2. Adverse events, including symptoms, and abnormal findings on physical examinations, neurological examinations, standard laboratory tests, and PK analysis of AZD0530
**OTHER KEY OUTCOME MEASURES / STUDY PROCEDURES**	Screening ^{18}F-Florbetapir PET, Volumetric MRI, LP sub-study, ADAS-Cog, MMSE, ADCS-ADL, CDR, NPI, genotyping, and biomarker labs

1.0 INTRODUCTION

Alzheimer's disease (AD) is the most common dementing illness and afflicts over 5 million in the USA now, with an annual health care burden near $200 billion[3]. Unfortunately, no therapy available today modifies the course of AD. The clinical dementia of Alzheimer's disease (AD) is coupled to a distinct pathology, with senile plaques consisting of Amyloid-ß (Aß) peptide, and with neurofibrillary tangles consisting of hyperphosphorylated tau protein. The existence of rare autosomal dominant cases of AD caused by mutations of the Amyloid-ß Precursor Protein (APP) or the Presenilin (PS1 and PS2) processing enzymes that produce Aß provides genetic proof that APP/Aß pathways can trigger clinical AD[4-7]. Other APP mutations reduce AD risk[8]. Tau mutations cause dementia in Fronto-Temporal Lobar Degeneration[9]. Biomarker studies of late onset non-familial AD now span the progression of disease from pre-symptomatic stage to mild cognitive impairment to AD[10-12]. Such observations have revealed that Aß dysregulation, as detected by CSF levels or by Positron Emission Tomography, is the earliest detectable change of the AD process, consistent with Aß serving as the trigger for the disease.

Large extracellular and inert plaques of amyloid mark the pathology, but attention has focused on conformationally distinct soluble oligomers of Aß (Aßo) as being neurotoxic[13-16]. Specifically, neurotoxicity is characterized by synaptic malfunction, and is accompanied by loss of dendritic spines. Chronically, synaptic changes are followed by neurofibrillary tangles, by neuro-inflammation and by neuronal cell loss.

Src Family Kinase, Fyn and Synaptic Function

Fyn is a member of the Src family of intracellular non-receptor tyrosine kinases family[17]. There are nine members of Src family kinases. Five of them (Src, Fyn, Lck, Lyn, Yes) are expressed in the central nervous system, but Src and Fyn are most highly expressed in the brain. Fyn activity, like that of other Src family kinases, is regulated by intramolecular interactions that depend on equilibrium between tyrosine phosphorylation and dephosphorylation. In the basal state, catalytic activity is constrained by intramolecular interactions, such as engagement of the SH2 domain by a phosphorylated C-terminal tyrosine 527. Disruption of these interactions by phosphorylation at Tyr 416 in the activation loop of the kinase domain and/or by dephosphorylation of Tyr 527 results in Fyn activation[18].

There are multiple lines of evidence linking Fyn kinase function to synapse plasticity, and the synaptic dysfunction central to AD. Fyn has been localized to the Post Synaptic Density fraction of the brain and amongst its substrates are receptors for the major excitatory transmitter, glutamate. Fyn regulates glutamate receptor trafficking and synaptic plasticity[2,19-21]. Specifically, Fyn is known to phosphorylate the NMDA-type glutamate receptor subunits, NR2A and NR2B[22]. Treatment of rats with transient middle cerebral artery occlusion using an SFK inhibitor (different from AZD0530) has revealed a neuroprotective action, reducing stroke volumes[23].

AZD0530

AZD0530 is an inhibitor of Src and Abl family kinases[1]. It has been developed as treatment for malignancies because these kinases play a role in tumor invasion and proliferation. However, the Src family kinases (SFKs) are highly expressed in the brain and have major effects on synaptic plasticity[2]. Moreover, recent studies have shown that Fyn is aberrantly activated by specific conformations of the Amyloid Beta (Aß) peptide from Alzheimer's disease (AD). Genetic deletion of Fyn rescues AD deficits in preclinical models[42, 49]. This project will test the potential benefit of AZD0530 for Alzheimer's disease modification.

1.1 Primary Aims

Aim 1.

To assess the effect of treatment with AZD0530 on 52-week reductions in fluorodeoxyglucose positron emission tomography (^{18}F-FDG PET) measurements of the cerebral metabolic rate for glucose (CMRgl) using statistical parametric mapping (SPM) statistical region of interest (sROI) in subjects with mild AD.

Aim 2.

To assess the safety and tolerability of treatment with AZD0530 over a 52-week period in subjects with mild AD as assessed by analysis of adverse events, including symptoms, and abnormal findings on physical examinations, neurological examinations, standard laboratory tests, and PK analysis of AZD0530.

1.2 Secondary Aims

Aim 3.

To assess the effect of treatment with AZD0530 on ADAS-cog, MMSE, ADCS-ADL, CDR-SB, and NPI in participants with mild AD.

Aim 4.

To assess the effect of treatment with AZD0530 on the rate of change in: total brain volume, ventricular volume, hippocampal volume, and entorhinal thickness, using volumetric magnetic resonance imaging (MRI).

Aim 5.

To assess the effect of treatment with AZD0530 on CSF biomarkers of AD (particularly CSF total tau and CSF ptau).

Aim 6.

To assess the influence of APOE genotype on the effects of treatment with AZD0530.

2.0 PRELIMINARY STUDIES

2.1 Summary of Pre-Clinical Findings

Fyn is Implicated in AD by Studies of Aß Oligomer Action

A critical early step in AD pathophysiology is the process by which Aßo interacts with the neuronal surface to trigger downstream pathology, and studies of this

pathway have further implicated Fyn in AD pathophysiology. In the only reported genome-wide unbiased screen for Aßo binding sites, Cellular Prion Protein (PrPC) was identified[24]. Aß binding to PrPC has consistently been shown to be of high affinity and is oligomer specific, with little or no affinity for fibrillary or monomeric states[24-27]. *In vivo*, PrPC is not essential for certain Aß-related phenotypes[26-29], but is required for cell death *in vitro*, for reduced survival of APP/PS1 transgenic lines, for epileptiform discharges, for synapse loss, for serotonin axon degeneration and for spatial learning and memory deficits[4, 30-36]. Critically, the ability of human AD brain-derived Aß species to suppress hippocampal synaptic plasticity requires PrPC, and human AD contains PrPC-interacting Aßo species and Aß– PrPC complexes[4, 37-39].

Once Aßo forms and binds to PrPC at the cell surface, changes in neuronal biochemistry occur. Fyn was a candidate downstream mediator because PrPC phenotypes in fish and worm require Fyn[40, 41], because Fyn regulates synaptic plasticity[2, 19-21], and because Fyn interacts with Tau and modulates phenotypes in mouse AD models[42, 7]. It has been shown that both PrPC and Fyn are enriched in the Post-Synaptic Density (PSD)[4]. It is possible to monitor the activation of Src family kinases (SFK) by phospho-specific epitopes. Cultures for SFK activation after exposure to Aßo[4] were examined. Wild type cortical neurons increase pY416-Fyn (SFK) in response to Aßo. This antibody detects pY416 in several SFKs, but kinase-specific immunoprecipitations demonstrate that PrPC-dependent activation is Fyn-specific[4]. In *Prnp*-/- cultures, activation of Fyn by Aßo is eliminated. Having previously detected PrPC-interacting Aß in human AD brain[51], it was assessed whether these assemblies also activate neuronal Fyn. AD brain extracts at 6 µg protein/ml stimulate Fyn activation in mouse cortical cultures, but Control brain extracts do not[4]. Thus, TBS-soluble Aß derived from human AD stimulates neuronal Fyn via PrPC, as confirmed in a subsequent study by Lesne, Aguzzi, and colleagues[44].

Fyn Activation by Aßo–PrPC Alters Synaptic Function

NMDA-Rs play a key role in synaptic plasticity and AD. Intracellular segments of NR2A and NR2B subunits contain tyrosine residues phosphorylated by SFKs[45]. Thus, Aßo/PrPC-mediated Fyn activation may be directed to NMDA-R. It has been shown that Aßo induces a dose-dependent increase in the phosphorylation of NMDA-R, specifically the Fyn-specific phosphorylation of NR2B at Y-1472[4]. Aßo-induced NR2B phosphorylation is eliminated in *Prnp–/–* cultures, in *Fyn–/–* cultures, and by 6D11 anti-PrPC antibody. Moreover, the roles of PrPC and Fyn are gene-dose-dependent, being reduced in heterozygous neurons[4]. The effect is biphasic, such that NR2B phosphorylation is enhanced during the first 15 minutes with Aßo, but phosphorylation is suppressed after 1-3 hours[4].

Phosphorylation of NR2B at Y1472 is known to reduce AP-2 mediated endocytosis[21]. Investigators have examined the extent to which NR2B is accessible at the cell surface versus being sequestered intracellularly. In concert with the NR2B-pY-1472 increase, surface NR2B increases shortly after Aßo exposure[4]. A calcium-sensitive fluorescent dye was utilized to monitor intracellular calcium in cortical neurons. NMDA produces increased fluorescence signal[4]. Pretreatment with Aßo for 15

minutes generates significantly increased NMDA-induced signal, as predicted. By 60 minutes, when NR2B receptors are dephosphorylated and internalized, NMDA-induced calcium signals are suppressed. One hour pretreatment with Aßo suppresses glutamate responses in WT, but not in *Prnp–/–* or *Fyn–/–* neurons[51]. Thus, Aßo-induced, PrPC-mediated alterations in NMDA-R create transient increases and then decreases in neuronal calcium.

It was considered whether the transient increase in surface NR2B might lead to a brief period of excitotoxicity. Brief exposure to Aßo reduces cell viability, with a release of 10% of cellular LDH. Genetic deletion of either Fyn or PrPC expression rescues neurons from Aßo[4]. Heterozygosity for null alleles of *Prnp* or *Fyn* significantly reduces LDH release[4]. Thus, Aßo requires PrPC to induce Fyn activation and subsequent NR2B phosphorylation. This phosphorylation is associated with transient increase in NR2B at the cell surface with consequent excitotoxicity.

A hallmark of AD is synaptic loss[46]. In vitro studies have described dendritic spine loss after acute Aßo exposure[14, 47, 48]. To assess the roles of PrPC and Fyn in Aßo-induced spine loss, neurons were cultured from embryos homozygous for null alleles[4]. Spine destabilization by Aßo is eliminated in *Prnp–/–* and *Fyn–/–* neurons. These studies suggest that Fyn plays a central role in coupling Aßo and PrPC to changes in neuronal function. Consistent with this hypothesis, when Fyn mutants are crossed with APP transgenic mice, Fyn gain-of-function enhances AD-related phenotypes while Fyn loss-of-function ameliorates AD-related phenotypes[42, 49].

Fyn Implicated in AD by Interaction with Tau

Neurofibrillary tangles are composed of hyperphosphorylated tau and are key pathological markers of AD. Similar to studies of Aßo, studies of tau have implicated Fyn mechanistically in AD. Fyn physically associates with tau, and can phosphorylate tyrosine residues near the amino terminus[5-8]. Moreover, the Aßo–PrPC complex-driven activation of Fyn leads to downstream tau phosphorylation[44]. Critically, Fyn and tau interact genetically to modulate synapse loss, behavioral deficits and electroencephalographic abnormalities in APP transgenic mice[42, 49, 50]. Dendritic tau is required to deliver Fyn to the post-synaptic density[9]. Without functional tau, Fyn is uncoupled from NMDA-Rs, and Aß toxicity is rescued[9]. Two studies have reported a genetic association between SNPs at the *FYN* locus and CSF-tau levels in AD, although this was not observed in all cohorts[51, 52]. Thus, PrPC/Fyn signaling appears to couple Aß and tau pathologies and Fyn can be considered a lynchpin of AD pathophysiology.

AZD0530 Inhibits Fyn

AZD0530 is an inhibitor of Src Family kinases, blocking Src, Fyn, Yes and Lyn, with 2-10 nM potency[53]. AZD0530's specific inhibition of Fyn and SFKs has led to its development as therapy for solid tumors, because Src family kinases regulate tumor cell adhesion, migration and invasion, and also regulate proliferation[53]. Clinical tolerability and oral bioavailability have been demonstrated, but Phase 2 studies have demonstrated limited benefit as a single agent in specific oncological indications[54-58]. For tumor cell migration and for oncological applications, it is

estimated that >98% kinase inhibition is required so clinical doses have targeted concentrations >20-fold above the kinase IC50, in the 200-1000 nM concentration. Importantly, CNS preclinical effects, and therefore the anticipated AD dosing, are achieved with much lower concentrations in the 5-50 nM range with 50-95% target inhibition.

Efficacy And Pharmacodynamics Of AZD0530 in AD Model Mice

To test the ability of AZD0530 to treat learning and memory deficits caused by AD pathophysiology, Yale investigators treated WT and APPswe/PS1ΔE9 transgenic mice with the compound. The treatment was started when mice were 11-12 months of age. At this age, other cohorts from previous studies have shown a pronounced learning and memory deficit. The dosing was on a twice a day schedule by oral gavage and control groups received vehicle. The identity of the active versus vehicle groups and the transgene status were unknown to the researchers handling the mice and collecting the behavioral data. Mice were treated with 0, 2 or 5 mg/kg/d of AZD0530. Both 2 and 5 mg/kg/d doses were tested against vehicle in a first cohort and then (because the initial results suggested that 5 mg/kg/d was required for benefit) a second cohort of mice was tested with 0 versus 5 mg/kg/d. The data from the two cohorts were pooled. Different tests were performed at 1-3 weeks (early) or 4-6 weeks (late) time points. Mice were tested in the Morris water maze at both ages, and also in Novel Object Recognition tests at the later time points. After memory testing was complete, the animals were sacrificed without ceasing their drug regimen for histological analysis.

The results of the pooled Morris water maze data for wild type and transgenic mice treated for 4-5 weeks with vehicle or 5 mg/kg/d AZD0530 are shown in Fig. 1 (left panel). The learning trials show wild type mice have progressively shortened latencies to a hidden platform, and this is not altered by treatment with 5 mg/kg/d AZD0530. The transgenic mice are impaired and do not show as much learning, with significantly prolonged latencies on the later blocks of swim trials. Treatment with AZD0530 for 4-5 weeks eliminated the transgenic deficit, normalizing latencies to the hidden platform on trial blocks 4-6.

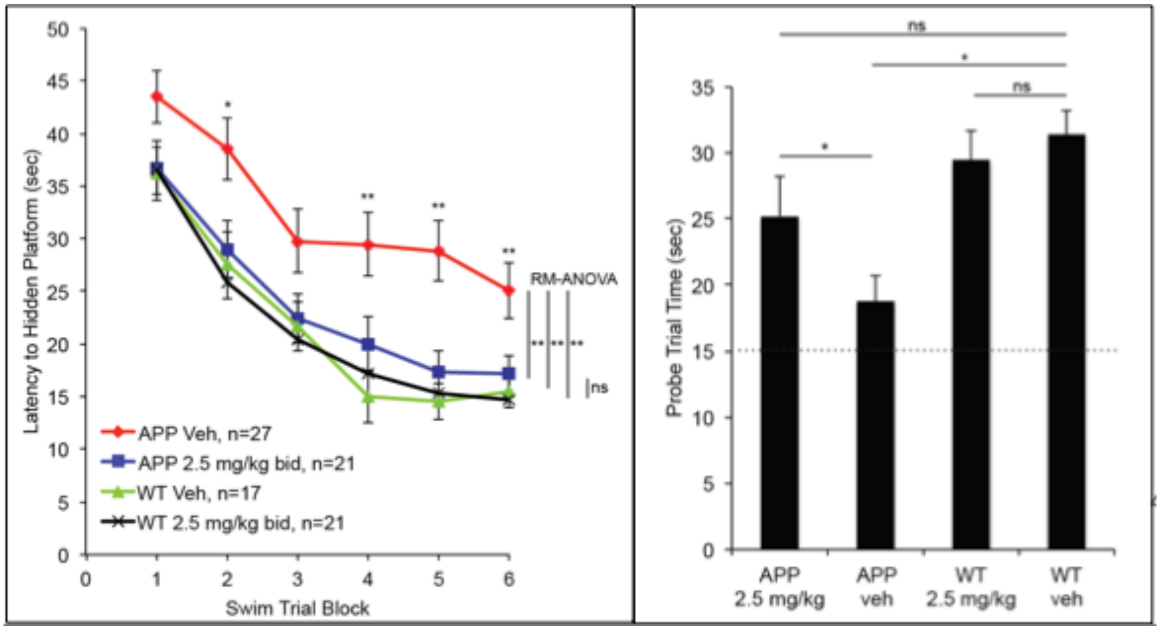

<u>Figure 1. AZD0530 improves learning and memory in transgenic AD mice.</u>
Left panel. *Spatial learning is plotted as latency to find a hidden platform in a Morris water maze at age 11-12 months. Mice were treated bid. by oral gavage for 3-4 weeks prior to the start of testing with 0 or 5 mg/kg/d AZD0530 and treatment was continued through the testing period. Mean ± SEM for the indicated numbers of mice per genotype/treatment group. By Repeated Measures ANOVA with Tukey post-hoc pairwise comparisons across trial blocks 3-6, the vehicle-treated APPswe/PS1ΔE9 group differed from each of the other groups (**P < 0.001), whereas none of the other groups differed from each other (p > 0.05). For specific trial blocks, the vehicle-treated APPswe/PS1ΔE9 group differed from each of the other groups, ** P < 0.005 or * P < 0.05, whereas none of the other groups differed from each other (P > 0.05).*
Right panel. *Memory performance during a 60 sec probe trial, 24 hr after learning, where time spent in the target quadrant was measured. Random chance is 15 sec. Mean ± SEM for n=9-14 in each group. Target quadrant time differed by one-way ANOVA with post hoc LSD pairwise correction for the indicated comparisons (*, P<0.05).*

After the learning trials, the mice were tested the subsequent day in a memory test, the 60 second probe trial with the hidden platform removed (Fig. 1, right panel). Random chance results in 15 sec occupancy in each quadrant. The wild type mice with or without 5 weeks AZD0530 treatment preferred the target quadrant, with typical times of 30 seconds compared to an average of 10 sec in each of the non-target quadrants. In contrast, the APPswe/PS1ΔE9 mice treated with vehicle control spent much less time in the target quadrant, performing close to the chance level of 15 seconds. The AZD0530 5 mg/kg/d treated group showed the same strong preference for the target quadrant as did WT mice. Thus, this dose of AZD0530 fully rescued both learning and memory deficits in aged AD transgenic mice. Shorter

treatment duration or lesser doses did not have significant effects in these tests (not shown).

Dog Toxicology Studies

Previous 6-month dog and rat toxicology studies had been completed by AstraZeneca, and cover 6-month exposure in humans. The FDA advised that a 9-month dog study is the appropriate preclinical study to support 12-month dosing in humans.

From the AstraZeneca Investigator's Brochure, Edition 10, the C-max and AUC values for dosing in dog at 5 mg/kg produces exposure equivalent to 125 mg in human, and the exposure in dogs with multiple dosing is linear from 0.5 to 5 mg/kg oral daily dose. Therefore, investigators at Yale initiated a 9-month dog toxicology study (8 dogs per dose) at 0 mg/kg/d, 0.5 mg/kg/d (NOAEL in 6 month), 2 mg/kg/d (equivalent to 50 mg daily in humans) and 5 mg/kg/d (a dose with mild GI toxicity in the 6-month study and equivalent to 50 mg daily in humans). This study was recently completed (See report "AZD0530: A 39-week Oral Gavage Toxicity Study in Dogs") and supports a 12-month human study.

2.2 Summary of Clinical Findings

Phase 1b Results

"A Phase Ib Multiple Ascending Dose Study of the Safety, Tolerability, and CNS Availability of AZD0530 in Alzheimer's Disease" (HIC #1303011664; ClinicalTrials.gov identifier NCT01864655) has been completed at Yale. The primary aims of the study were to assess the safety and tolerability of oral AZD0530 in patients with AD and to determine dose levels that are well tolerated in AD patients and provide CSF concentrations predicted to slow AD.

Subjects were enrolled in three Cohorts of 8 subjects each. Each cohort consisted of subjects receiving AZD0530 (n=6) or Placebo (n=2) PO daily. The first cohort received 50 mg, the second 100 mg and the third 125 mg.

All subjects completed the study in Cohorts 1 and 2 and there were no serious adverse events (SAEs), significant laboratory abnormalities, or early terminations. In Cohort 3, there was one serious adverse event (SAE) that was also reported to the FDA as a Suspected Unexpected Serious Adverse Reaction (SUSAR), but all other subjects completed the study without SAEs, or early terminations. Adverse event frequencies by treatment group are shown in Table 1.

Two subjects receiving AZD0530 125 mg daily had significant laboratory abnormalities that subsequently reversed: elevated serum creatinine, possibly related to study medication; worsened renal insufficiency, unlikely related to study medication. There were no significant hematological changes. The single SAE occurred in an 85 year old white female who was receiving 125 mg of AZD0530. After 9 days of treatment, she was hospitalized with 4 days of fatigue, anorexia, and body aches, and 1 day of shortness of breath, in the setting of her diuretic being held

for one day. The diagnosis was of congestive heart failure, bronchitis and/or atypical pneumonia. She was treated with additional diuretic (furosemide) and antibiotics (levofloxacin). Study drug was discontinued, and not restarted. She was discharged to short-term rehab (SNF) and has since done well, returning to baseline health. Because previous AstraZeneca experience with AZD0530 included a few subjects with reversible interstitial lung disease deemed to be possibly related to AZD0530, the subject was also evaluated by Yale interstitial lung disease expert, Danielle Antin-Ozerkis, MD. The impression was most consistent with CHF precipitated by pneumonia. However, Dr. Antin-Ozerkis concluded that "Drug toxicity is difficult to entirely rule out." The overall conclusion is that this event was "possibly related" to study drug. This SAE was reported to the DSMB, the Yale HIC, the NIH, and the FDA (as a SUSAR).

Table 1. Adverse Events (preferred terms) by Treatment Group

Adverse Events are per participant (counted only once for each adverse event)

	Treatment group (AZD0539 dose; n = 6 per group)				
**Adverse event**	**Placebo**	**50 mg**	**100 mg**	**125 mg**	**Total**
**Any adverse event***	**3**	**4**	**5**	**4**	16
Diarrhea	2	0	1	2	5
Headache	2	1	1	1	5
Fatigue	1	0	0	2	3
Nausea	2	0	0	1	3
Pneumonia, atypical/bronchitis	0	0	0	1	1
Congestive heart failure	0	0	0	1	1
Renal insufficiency, worsened	0	0	0	1	1
Elevated serum creatinine	0	0	0	1	1
Cough, worsened	0	0	0	1	1
Postnasal drip	0	0	0	1	1
Pulmonary hypertension	0	0	0	1	1
Anorexia	0	0	0	1	1
Tinnitus	0	0	0	1	1
Myalgias	0	0	0	1	1
Squamous cell carcinoma	0	0	1	0	1
Basal cell carcinoma	0	0	1	0	1
Wrist pain	0	0	1	0	1
Noncardiac chest pain	0	0	1	0	1
Upper respiratory infection	0	1	0	0	1
Flu-like symptoms	0	1	0	0	1
Lightheadedness	0	1	0	0	1
Vomiting	1	0	0	0	1

*Includes participants who reported at least one adverse event. The effect of treatment group is not significant for any adverse event or individual adverse events (Fisher's exact test, $P > 0.05$).

All subjects underwent standard clinical efficacy assessments at baseline and at Week 4 endpoint. These included Mini Mental Status Examination (MMSE), Alzheimer's Disease Assessment Scale, cognitive subscale (ADAS-cog), Alzheimer's Disease Cooperative Study Activities of Daily Living Scale (ADCS-ADL), Clinical Dementia Rating, sum of boxes score (CDR-SB), and Neuropsychiatric Inventory (NPI). There was a tendency for MMSE scores to improve over time, which may have

been due to practice effects as the MMSE was also administered at weekly visits as a safety measure. No statistically significant effect of treatment was observed on any of clinical efficacy assessment measures, which was not surprising given the small samples sizes and the short duration of treatment with an investigational therapy that is being developed primarily for longer-term disease modifying potential.

The Phase 1b study collected both CSF and plasma for AZD0530 levels using a GLP-validated LC/MS/MS assay (MPI Research, Mattawan, Mi). There was a close correlation between free AZD0530 in plasma and CSF AZD0530 (Fig. 3, left panel). CSF levels were about 1/3rd of plasma free drug levels. The free drug levels are calculated from the total plasma drug levels based on the molecular weight of the compound and a protein binding percentage in plasma of 91%. Comparisons were performed of the CSF levels across the dosage groups. There was variability within any one group, but clear dose dependency (Fig. 3, right panel). The increase in CSF level was greater than linear, with a 3-fold increase from 50 to 100 and a 2-fold increase from 100 to 125 mg, and this was most consistent with an exponential relationship. The trough CSF at 50 mg was 0.5 ng/ml to 1.2 ng/ml = 0.9 to 2.2 nM. The trough CSF at 100 mg was ng/ml to 4.5 ng/ml = 2.1 to 8.3 nM. The trough CSF at 125 mg was 1.4 ng/ml to 7.6 ng/ml = 2.5 to 14.0 nM. Using the ratio of CSF:Brain in mouse, the estimated human brain concentrations at 50 mg = 3 to 7 nM, at 100 mg = 7 to 27 nM, and at 125 mg = 8 to 46 nM. As mentioned above, the Fyn Ki for AZD0530 is 5 to 10 nM. Moreover, the mouse trough CSF at a dose effective in rescuing memory for transgenic mice is 3.1 to 7.6 ng/ml = 5.8 to 14 nM. This value overlaps with the human 100 mg levels and spans the human 125 mg range (Fig. 3, right panel).

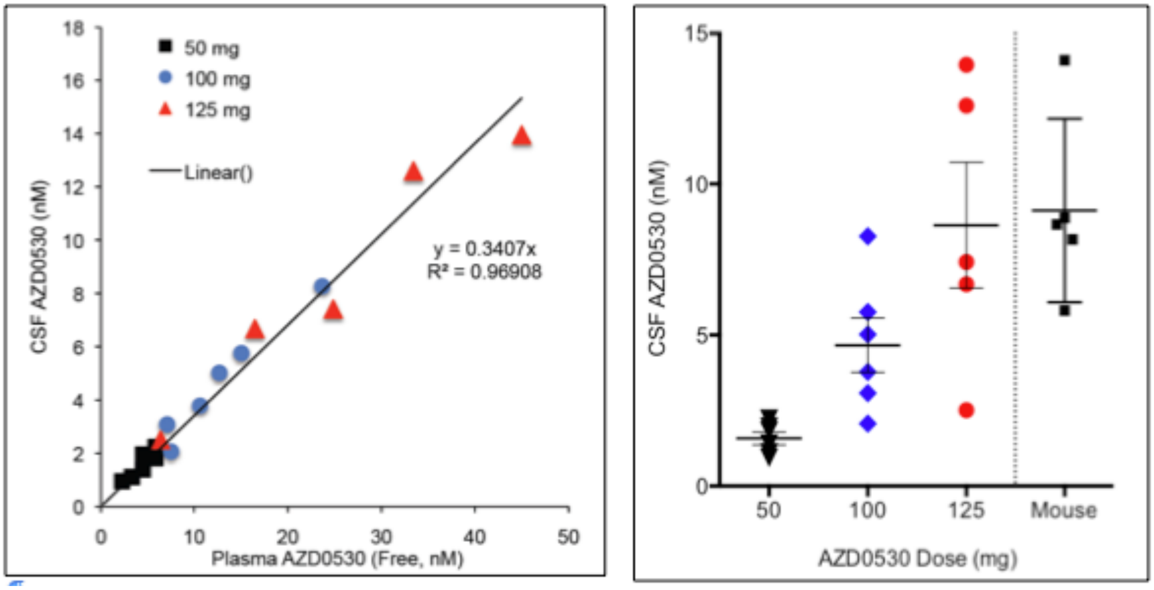

Figure 3. AZD0530 in Human CSF at Different Doses.
Left Panel*. Relationship between CSF and plasma levels of AZD0530. Each point is from a different individual. The different dose groups are illustrated with different colors. There is a tight correlation of plasma and CSF level as indicated.*
Right Panel*. Each point represents trough CSF AZD0530 level from a different human subject for the left three columns. The mouse trough CSF values are derived from brain levels at the 5 mg/kg/d dose that rescued memory deficits in Fig. 1. Bars represent mean ± sem.*

2.3 Safety and Compliance

AZD0530 has been extensively studied in human subjects, including published studies with healthy individuals[89, 90], and patients with solid tumors[91, 92]. AstraZeneca has extensive safety data of this drug in human subjects, described in detail in the Investigator Brochure (IB), and described in detail below. Notably, in a multiple ascending dose study, AZD0530 doses of 60 mg to 185 mg were well tolerated, and although adverse events were noticeably more frequent and severe at the 250 mg dose, no major safety issues were identified from adverse events at any of the doses studied.

To date, 187 healthy volunteers and 421 patients with advanced cancer have received single or multiple once-daily oral doses of AZD0530 (Saracatinib). In a single ascending dose study, the maximally tolerated dose (MTD) was considered 1000mg. In a multiple ascending dose study, the MTD was considered 250mg, although a toxicity-limiting dose was not pursued. In patients with advanced cancer, the MTD was 175mg. Existing preclinical toxicology in two species demonstrates safety through 6 months, supporting chronic administration for this duration in humans. In clinical oncology, the duration of AZD0530 treatment has been 2-12+ weeks in most trials.

The doses proposed in this study, ranging from 100-125 mg have generally been well tolerated in both healthy volunteers, and in patients with advanced solid tumors. The most common adverse events at these doses have included rash, headache, and diarrhea. Less common adverse events have included nausea, vomiting, anorexia, anemia, palpitations, fatigue, influenza-like symptoms, and abnormal liver function tests. Other adverse effects at higher doses have included upper respiratory infection, myalgias, abdominal pain and tenderness, and epididymitis.

Importantly, with AZD0530 monotherapy, febrile neutropenia or other serious hematologic adverse events have not been documented in doses equal to or lower than 175 mg. In a total of 7 phase 1 trials, including 187 healthy volunteers, no major safety issues were identified from adverse events in any of the doses studied, up to 250 mg daily (AstraZeneca Investigator's Brochure, Edition 10, May 9, 2013). There is a potential for mild decreases in values for both white cell count and

platelets with doses of 125 mg and above, but these abnormalities never fell below reference levels, and levels normalized while patients remained on (AstraZeneca Investigator's Brochure, Edition 10, May 9, 2013). The timing for hematologic effects of AZD0530 is within 14 days of drug initiation, providing the rationale for the 30-day monitoring period in the phase 1 study (AstraZeneca Investigator's Brochure, Edition 10, May 9, 2013). It should also be noted that 2/3 of the safety data have been generated from patients with solid tumors, and some of these patients were included in the study despite mild baseline pancytopenia.

3.0 BACKGROUND AND SIGNIFICANCE

3.1 Rationale for Study

Despite the enormous and growing burden of AD, there remains no effective disease-modifying therapy today. The approaches now in clinical trials are limited in their mechanism of action. Most AD clinical trials have centered on efforts to alter Aß itself, either its production or clearance or aggregation. No major trial has centered on the signal transduction downstream of toxic Aß species. In this sense, the focus on Fyn inhibition is highly innovative in the AD field. There are efforts to develop approaches to targeting tau and tau kinases in AD, but few have advanced in clinic trials, and none provides a unified approach to Aß and tau. Fyn is unique as an AD target in being central to Aß signal transduction and having major functional interactions with tau as well. Thus, the target is innovative in addressing toxicity related to both hallmarks of AD pathology.

If AZD0530 blocks Fyn kinase activation in the brain of AD patients, it is predicted that it will provide the first disease-modifying therapy for AD.

3.1.1 Rationale for Primary and Secondary Endpoints

A major challenge in the development of AD treatments is the ability to evaluate them in a rapid, cost-effective, and rigorous way[59]. Due to the slow progression of cognitive and functional decline and the high test–retest variability of the clinical outcomes used in randomized clinical trials (RCTs), researchers have sought to develop biomarkers that reflect AD progression or pathology and that could be used to assess putative AD-slowing treatments with better statistical power[55, 56]. An established biomarker of AD progression is fluorodeoxyglucose positron emission tomography (^{18}F-FDG PET) measurements of regional cerebral metabolic rate for glucose decline[59-61] (CMRgl). Studies spanning more than twenty years with ^{18}F-FDG PET have reported characteristic and progressive CMRgl reductions in posterior cingulate, precuneus, parietal, temporal and frontal regions in patients with AD and mild cognitive impairment (MCI). Moreover, longitudinal decline in CMRgl is strongly associated with concurrent cognitive and functional decline[62, 63].

Kewei Chen et al.[63] at Banner Alzheimer's Institute, have introduced the use of an empirically pre-defined statistical region-of-interest (sROI) to characterize CMRgl declines with optimal power and freedom from multiple comparisons, and to estimate the number of subjects needed to characterize AD-slowing treatment

effects in multi-center RCTs. They described twelve-month CMRgl declines in 69 probable AD patients, 154 amnestic mild cognitive impairment (MCI) patients, and 79 cognitively normal controls (NCs) from the AD Neuroimaging Initiative (ADNI) using statistical parametric mapping (SPM). The AD and MCI groups each had significant twelve-month CMRgl declines bilaterally in posterior cingulate, medial and lateral parietal, medial and lateral temporal, frontal and occipital cortex, which were significantly greater than those in the NC group and correlated with measures of clinical decline. Using a training dataset comprised of 40% of the subject sample, these authors determined the settings associated with optimal CMRgl decline. The following settings were identified as optimal for the AD group a) a spatial resolution of 8 mm FWHM (i.e., no additional smoothing), b) a spared region defined by a significance threshold of $p<0.0005$, consisting of 6665 voxels associated with twelve-month regional-to-whole-brain CMRgl increases and located primarily in white matter and cerebellum, and c) a decline sROI defined by a significance threshold of $p<0.0005$, consisting of 32,807 voxels associated with twelve-month regional-to-spared sROI decline and located in the same regions known to be preferentially affected in cross-sectional and longitudinal brain-mapping studies of AD. They then applied these sROIs to assess CMRgl declines in independent test dataset comprised of the remaining 60% of the subject sample and estimated the samples needed for AD patients per treatment group to detect a AD-slowing treatment effect in a twelve-month, multi-center RCT with 80% power and two-tailed alpha=0.05. Note: this power analysis forms the basis of this study's proposed sample size.

Secondary Endpoints. Although this trial will exploit the statistical power of a surrogate biomarker (glucose metabolism) for the primary efficacy endpoint, it will also acquire data for standard clinical endpoints to help power a future Phase 3 pivotal trial. These endpoints (Aim 3) include ADAS-cog, MMSE, ADCS-ADL, CDR-SB, and NPI. Finally, as additional secondary endpoints, other recognized outcome biomarkers of AD progression have been selected in Aim 4 (MRI volumetrics) and Aim 5 (CSF biomarkers).

Volumetric MRI allows the in vivo assessment of brain structure volume and provides a measure of atrophy rate. Results from volumetric MRI studies suggest that the patterns of atrophy in AD, which mirror the pathological progression of the disease, can reliably be detected and tracked across time (for review, see Atiya et al.[64]). Atrophy of the medial temporal lobe, including hippocampus and entorhinal cortex, has long been described in volumetric MRI studies of AD[65]. Hippocampal volume derived from MRI correlates with histological hippocampal volume and degree of neuronal loss and AD pathology[66], and entorhinal cortical thickness change appears to be an early and sensitive indicator of neurodegeneration associated with AD[67, 68]. Longitudinal MRI measures of regional and whole-brain volumetric change provide a valuable complement to cognitive measures in that they are not influenced by temporary symptomatic improvements, and they provide an early index of the study drug's ability to reach the target organ and have an effect on AD-related atrophy.

Subjects will have the option to participate in the CSF Substudy, in which they will undergo lumbar punctures (LPs) at the Baseline visit and Week 52 visit. In this substudy, the following putative biomarkers of AD will be measured: CSF total tau, and CSF pTau. These markers will be assessed at baseline and after 52 weeks of treatment with either AZD0530 or placebo. These putative biomarkers are among the leading candidates for AD biomarker development[78, 79]. For example, Gilman et al.[80] reported a highly significant ($p < 0.001$) effect of Aβ immunization (with AN1792) on CSF tau with a total sample size of only 21 (N = 11 antibody responders and 10 placebo control participants). Critically, Aßo/PrP/Fyn signaling has been linked to pTau in preclinical studies[44]. Although not expected to be altered by treatment with AZD0530, CSF Aβ42 will also be assessed.

3.2 Rationale for Dosage Selection

The dosing plan is based on the results of the Phase 1b trial, as well as studies of "Efficacy and Pharmacodynamics of AZD0530 in AD Model Mice" (See Section 2.0). Among subjects in the Phase 1b study in the 100 mg dose group, those with steady state total plasma levels above the median value 70 ng/mL in the Phase 1 MPI Research laboratory results (a calculated free drug concentration of 11.7 nM) also attained CSF levels of at least 5 nM, within the range of the Fyn Ki for AZD0530 (5 to 10 nM) and the efficacious levels in AD model mice (5.8 to 14 nM). In preparation for the Phase 2 trial responsibility for conducting the plasma drug level measurement assay has been transferred from the MPI Research laboratory to the UCSD laboratory. Cross validation studies have shown that plasma AZD0530 values are very tightly correlated between laboratories, but the UCSD laboratory values are approximately 1.5 times higher than the MPI laboratory used for Phase 1. Based on these observations, the dosing plan aims to maximize the number of subjects who achieve total plasma AZD0530 levels >100 ng/mL as measured at the UCSD laboratory, while minimizing the safety and tolerability problems that would result from forced titration of all subjects to 125 mg daily. From the Phase 1 results, it is expected that approximately 50% of subject starting on 100mg AZD0530 will be titrated up to 125mg AZD0530 daily.

3.3 Rationale for ^{18}F-Florbetapir PET and Biomarkers

To streamline the evaluation of AZD0530, a number of biomarkers—both of AD/Aβ pathogenesis and of AD progression—have been incorporated. A significant rate of amyloid PET negativity is evident in longitudinal studies that enroll subjects on clinical criteria alone—as high as 23% in the ADNI study[75]. Therefore, for study inclusion, an eligibility biomarker of AD and Aβ Pathogenesis—amyloid PET positivity—will be required to enrich the subject sample for individuals with true cases of AD and who are more likely to respond to AZD0530 as an inhibitor of Fyn in an Aβ-dependent pathway. Requiring evidence of fibrillar Aβ burden using PET imaging for study inclusion follows emerging diagnostic recommendations[73] and is designed to enhance the pathological specificity of the diagnosis of AD[74].

To augment statistical power, the surrogate biomarker of glucose metabolism has been selected for the primary efficacy Aim 1. Other recognized outcome biomarkers of AD progression have been selected in Aim 4 (MRI volumetrics) and Aim 5 (CSF biomarkers).

4.0 POTENTIAL RISKS AND BENEFITS ASSOCIATED WITH THIS INVESTIGATIONAL PRODUCT

4.1 Potential Risks and Benefits Associated with This Investigational Product

4.1.1 Potential Benefits

The study drug may slow cognitive decline in patients with mild AD. The results of the study might help people with a similar condition in the future.

4.1.2 Potential Risks

AZD0530 has been extensively studied in human subjects, including healthy individuals [89,90], and patients with solid tumors[91, 92]. AstraZeneca has extensive safety data of this drug in human subjects, described in detail in the Investigator Brochure (IB) and summarized in Risks Associated with Study Drug, AZD0530. Overall, the safety profile of the doses of AZD0530 proposed in this project is good, and the Phase 1b study found that this safety profile can be also be extended to a patient population with AD.

Conception must be avoided during maternal or paternal exposure to AZD0530. Women of childbearing potential are excluded from participation in this study and male study participants who are sexually active will be advised by site personnel regarding the use of appropriate forms of contraception and avoiding sperm donation for the duration of the trial and for a washout period of 1 week after cessation of study medication.

There have though been cases where AZD0530 has been associated with potentially serious neutropenia and thrombocytopenia, although this has not been seen in healthy volunteers with doses up to 250 mg daily.

To minimize potential risks in the study, subjects with unstable medical conditions will be excluded. Subject safety will be evaluated clinically and by laboratory measures (especially hematology studies for neutropenia, thrombocytopenia, and anemia), and thoracic high resolution CT (HRCT) if pulmonary symptoms arise. During the visits, vital signs will be recorded, and they will receive a physical and neurological examination by a study physician at screen and throughout the study (see Appendix 1, Schedule of events). Specific parameters for intervention discontinuation are detailed below.

4.2 Relationship of this intervention to current treatment practices in AD

Currently, there is no approved treatment to prevent progression of Alzheimer's disease. Approved treatments for Alzheimer's disease include the following AChE

inhibitors: donepezil (Aricept), rivastigmine (Exelon), and galantamine (Razadyne) which offer modest symptomatic relief (in terms of cognitive test scores and global functioning) for some patients but do not constitute standard treatment for the disease. Memantine (Namenda) is an approved treatment of moderate to severe Alzheimer's disease. AZD0530 is an investigational drug that may have a disease-modifying effect on AD. A placebo group is included in this study to allow for accurate assessment of safety, tolerability, and efficacy of AZD0530.

5.0 SAMPLE SIZE AND STATISTICAL PLAN

5.1 Randomization

Participants who have signed an informed consent and meet screening eligibility requirements will be randomly assigned using a 1:1 allocation ratio to AZD0530 or placebo by a random permuted block treatment assignment method, stratified by site.

5.2 Power and Sample Size Determination

Aim 1. ^{18}F-FDG PET

Sample size calculations were based on a two-sided, two-sample t-test to compare the differences in mean change in CMRgI from baseline to month 12 by treatment arm. The statistical software R (version 2.14.0), http://www.r-project.org, was used for all power calculations. Using the reported CMRgl decline mean and standard deviation of 0.0514 ± 0.0309 in Chen et al.[63], assuming 15% attrition, an alpha set to 0.05, a sample size of 128 participants (64 participants per arm) is needed to detect a treatment effect of 30%. Assuming 15% per year attrition rate, a total of 152 subjects need to be enrolled at the start of the study.

Aim 2. Safety and Tolerability

Power calculations were based on a two-sided chi-square test for detecting a difference between two proportions assuming a Type I error of 0.05. With a sample size of 64 patients in each group, and assuming an adverse event (AE) rate of 12.5% in the placebo group, there will be 80% power to detect an AE rate of 33.1% in the active treatment group (absolute difference in AE rates of 20.6% or higher). In other words, the AE rate will need to be as high as 33.1% in the active treatment group before there is sufficient power to assert that the result is statistically significant.

5.3 Selection of participants to be used in analysis

In general, biomarker and clinical outcome analyses will incorporate the modified intent-to-treat principle (mITT), namely, all randomized participants who have been given study medication with a valid baseline and at least one post-baseline measurement will be included in the analysis while the safety analysis will be conducted on the intent-to-treat (ITT) population (all randomized participants). All results will be reported as point estimates (proportions or mean differences across groups, as appropriate) and interval estimates (95% confidence intervals) with two-sided p-values denoting statistical significance. A basic serial gatekeeping procedure

will be used to maintain over-all experiment-wise Type I error at $a = 0.05$ (two-sided). The six hypotheses regarding (i) F-FDG PET, (ii) ADAS-Cog, (iii) ADCS-ADL, (iv) CDR-SB, (v) MMSE, and (vi) NPI will be tested in order using a basic serial testing strategy. If, for example, the *p*-value for FDG is ≥0.05, no subsequent hypotheses will be declared statistically significant. Otherwise if the *p*-value for FDG is <0.05, the ADAS-Cog hypothesis is tested by comparing the *p*-value for ADAS to 0.05. This strategy continues until a *p*-value is ≥0.05, or the NPI hypothesis is declared statistically significant with $p < 0.05$. Regardless of the outcome of the multiple testing strategy, *p*-values and 95% confidence intervals will be reported for all planned analyses. Statistical analysis will be conducted using the statistical software R (www.r-project.org).

Demographic and baseline characteristics will be compared between the study groups using Fisher's exact test for categorical variables, and two-sample t-tests for continuous variables. Appropriate non-parametric alternatives will be considered, if parametric assumptions fail.

5.4 Efficacy Analysis

5.4.1 Analysis of the Primary Outcome(s)

Aim 1. ^{18}F-FDG PET

The rate of CMRgI change will be compared between the two treatment groups using a linear mixed-effect model with CMRgI at each time point (including baseline) as the outcome variable. The model will include fixed effects for time (as a continuous variable), time-by-treatment interaction, age, and APOE; and participant-specific random intercepts. The model will constrain the two treatment groups to have the same mean at baseline. The hypothesis will be tested using the *p*-value corresponding to the mean group difference in rate of CMRgI change.

Aim 2. Safety and Tolerability

Summary statistics will be tabulated for the adverse events and clinical laboratory studies. Fisher's Exact Test will be used to compare the frequencies of adverse events or laboratory abnormalities between the participants who receive AZD0530 and those receiving placebo.

Population PK analysis of concentration-time data of AZD0530 will be performed using a Mixed Model of Repeated Measures (MMRM). The outcome measure will be change from baseline in AZD0530 at each follow-up visit. The model will treat time as a categorical variable and include fixed effects for the treatment-by-time interactions, baseline AZD0530, age, and APOE. The model will assume an unstructured correlation and heterogeneous variance with respect to time. If the unstructured correlation model fails to converge, simpler structures (autoregressive of order one and compound symmetric) will be assumed in turn. The hypothesis will be tested using the *p*-value for the mean group difference in AZD0530 at week 52.

5.4.2 Analysis of Secondary Outcomes

Aim 3. Clinical and Cognitive

A similar categorical time MMRM as above will be used to estimate the mean group difference in each secondary outcome at 52 weeks. The outcome measure will be change from baseline in the outcome at each follow-up visit. The model will treat time as a categorical variable and include fixed effects for the treatment-by-time interactions and baseline outcome. In addition, age and APOE4 genotype will be included in the model as fixed effect covariates. The model will assume an unstructured correlation and heterogeneous variance with respect to time. If the unstructured correlation model fails to converge, simpler structures (autoregressive of order one and compound symmetric) will be assumed in turn. The hypothesis will be tested using the *p*-value for the mean group difference at week 52.

Aim 4. Volumetric MRI

The volumetric MRI outcomes will include (i) hippocampal volume, (ii) ventricular volume, (iii) total brain volume, and (iv) entorhinal thickness. The regional volume changes will be determined by nonlinear registration between baseline and follow-up images, and expressed as % deformation per years from baseline. The rate of volumetric MRI change will be compared between the two treatment groups using an ANCOVA model with % deformation per year as the outcome variable and covariates for baseline regional volume (or entorhinal thickness), treatment, age, and APOE. The hypothesis will be tested using the *p*-value corresponding to the mean group difference in rate of volume change.

Aim 5. CSF Biomarkers

Similar to the analysis for Hypothesis 1, the rate of CSF total Tau and pTau change will be compared between the two treatment groups using a linear mixed-effect model with observations at each time point as the outcome variable. The model will include fixed effects for time (as a continuous variable), time-by-treatment interaction, age, and APOE; and participant-specific random intercepts. The model will constrain the two treatment groups to have the same mean at baseline. The hypothesis will be tested using the p-value corresponding to the mean group difference in rate of change.

Aim 6. APOE Genotype

This analysis will focus on the potential influence of APOE4 genotype on outcome measures. Analyses of subgroups defined by the presence or absence of the APOE4 allele (APOE+ versus APOE-) will be performed using analyses techniques analogous to the ones uses in Aims 1-5 to obtain separate assessment of treatment efficacy for both APOE4+ and APOE4- participants.

5.5 Interim Analysis

There are no planned interim analysis being conducted for this study, but the DSMB may modify this during ongoing safety monitoring.

5.6 Criteria for the termination of the trial

The trial may be terminated by the Project Director, NCATS and/or AstraZeneca based on issues of safety, feasibility, and DSMB recommendations.

6.0 STUDY DRUG AND CONCOMITANT MEDICATIONS

6.1 Name and Description of IP and Comparator

AZD0530 (saracatinib) is an inhibitor of Src Family kinases, blocking Src, Fyn, Yes and Lyn, with 2-10 nM potency[53]. A placebo comparator will be used. AstraZeneca will provide both study drug and placebo.

6.2 Dosage

The AZD0530 treatment group will initially receive 100 mg PO daily. At the Week 2 visit, total plasma drug levels will be measured (ADCS Biomarker Core, UCSD). Those subjects in the active treatment group who at week 2 are sufficiently compliant with dose regimen but have not attained a total plasma AZD0530 level of at least 100 ng/mL will be increased at the Week 4 visit to 125 mg of AZD0530 daily. All subjects will then remain on their Week 4 doses for the remainder of the study.

The placebo group will receive the placebo comparator for the duration of the study.

Study medication should be taken in the morning and may be taken with or without food.

6.3 Blinding

This is a double-blind study in which subjects and site personnel will be blinded to both treatment assignment and dose level. In order to maintain the blind, each subject will be prescribed the same number of tablets (3) each day throughout the trial – either all placebo or a combination of placebo and AZD0530. The following table describes the number and type of tablets to be taken each day based on treatment group and, for the AZD0530 group, dose level (see Section 6.2 for dose escalation rules).

	**Number of Tablets per day**	
	Baseline – Week 4	Week 4 – Week 52
**AZD0530 Treatment Group:**<br><u>without</u> week 4 dose escalation	50mg AZD0530: 2<br>placebo-for-125mg: 1	50mg AZD0530: 2<br>placebo-for-125mg: 1
**AZD0530 Treatment Group:**<br><u>with</u> week 4 dose escalation		placebo-for-50mg: 2<br>125mg AZD0530: 1
**Placebo Group**	placebo-for-50mg: 2<br>placebo-for-125mg: 1	placebo-for-50mg: 2<br>placebo-for-125mg: 1

A module within the Data Portal will be used to assign bottles to be dispensed to each subject at each visit as appropriate based on treatment assignment. The module will use the week 2 compliance and plasma drug level data to determine the need for a dose adjustment and will assign bottles for the remainder of the study accordingly.

6.4 Packaging/Dispensing/Labeling

Study medication will be packaged and dispensed according to the table below. The dosing schedule will be clearly explained to the caregiver before dispensing the study drug and it will be printed on the labels.

Dispensing Visit	# 50mg AZD0530 or Placebo tablets dispensed	# 125mg AZD0530 or Placebo tablets dispensed
**Baseline**	1 bottle with 80 tablets	1 bottle with 40 tablets (placebo only)
**Week 4**	2 bottles with 80 tablets each	2 bottles with 40 tablets each
**Week 13**	3 bottles with 80 tablets each	3 bottles with 40 tablets each
**Week 26**	3 bottles with 80 tablets each	3 bottles with 40 tablets each
**Week 39**	3 bottles with 80 tablets each	3 bottles with 40 tablets each

6.5 Storage

Medication should be stored in a facility at the site to which only the investigator, study coordinator and/or other designated personnel has access. Study medication must be stored at controlled room temperature (25° C, with excursions permitted to 15° – 30° C).

6.6 Drug Accountability

Study medication will be shipped to study sites from a central depot. The investigator or designee must receive and store study medication supplies in a secure area and dispense according to protocol. The participant will be advised to return all unused or partially used study medication to the investigator for counting and recording by study staff. All empty bottles and unused or partially used supplies will be counted and verified by a clinical monitor prior to being destroyed according to local site procedures.

6.7 Compliance

Site personnel will assess compliance at every visit after Baseline. Participants and study partners will be instructed to bring medication bottles and any unused study medication each visit. If they do so, site personnel will count returned tablets and evaluate compliance. If they do not bring medication with them to a visit, site personnel will discuss adherence to study medication regimen with the participant and study partner. Participants found to be < 80% or > 120% compliant will receive specific instruction from site personnel, including re-instruction on study procedures and additional telephone contact between visits.

Prior to the Week 2 visit, site personnel will contact the participant and study partner to remind them of dose regimen, in particular to ensure that the necessary level of compliance is achieved to accurately measure plasma study drug levels. Details regarding timing of contact and compliance assessment for the week 2 blood draw will be provided in the Procedures Manual.

For LP sub-study participants, a similar focus on compliance, with site personnel discussing the dose regimen with the participant and study partner , will occur in the period leading up to the final LP to ensure that the necessary level of compliance is achieved to accurately measure CSF study drug levels.

6.8 Breaking the Blind

Only in the case of an emergency, when knowledge of whether the patient has received the investigational product is essential for the clinical management or welfare of the patient, may the Investigator request to unblind a patient's treatment assignment. If the Investigator needs the blind to be unmasked for a patient for any reason, the Investigator must contact the Medical Monitor (and/or Project Director) to obtain an approval. Breaking the blind must be reported to the Coordinating Center within 5 business days, documenting the date, the site personnel exposed to the treatment assignment, and the reason the blind was broken. Refer to the study Procedures Manual for more detailed procedures related to breaking the blind and reporting.

6.9 Concomitant AD Medications

This protocol allows concomitant treatment with cholinesterase inhibitors and memantine, if on a stable dose for 12 weeks prior to screening.

Initiation of or modifications to concomitant AD medications during the course of the study should be discouraged, however, if a change occurs or a new medication is initiated, the site should consult with the Project Director and/or Medical Monitor for further guidance.

6.10 Other Concomitant Medications

6.10.1 Prohibited Concomitant Medications

Use of the following medications is prohibited within 4 weeks of screening and throughout the study except as noted in section 6.10.2:

- tricyclic antidepressants
- antipsychotics (except risperidone ≤1.5 mg/day, quetiapine ≤100 mg/day, olanzapine ≤5 mg/day, and aripiprazole ≤10 mg/day)
- mood-stabilizing psychotropic agents (e.g. lithium salts)
- psychostimulants
- opiate analgesics
- antiparkinsonian medications (except for non-parkinsonian indications)
- anticonvulsant medications (except gabapentin and pregabulin for non-seizure indications
- systemic corticosteroids
- medications with significant central anticholinergic activity
- anxiolytics or sedative hypnotics: Use of short- to medium-acting benzodiazepines for treatment for insomnia or anxiety is permitted. Use of sedatives or hypnotics should be avoided for 8 hours before administration of cognitive tests unless they are given chronically.

AZD0530 may partially inhibit the CYP3A4 enzyme, which is part of the P450 system. For this reason, subjects taking the following drugs (sensitive CYP3A4 substrates or CYP4A4 substrates with narrow therapeutic ranges, whose metabolism may be slowed by AZD0530) will be excluded from the trial: carbamazepine, colchicine, cyclosporine, disopyramide, fluticasone (except as a nasal spray which is allowable), oral steroids, quinidine, vinblastine, and vincristine. Subjects taking sildenafil, tadalafil, and vardenafil (also CYP3A4 substrates)—and their prescribing physicians—will be advised that these medications may be potentiated by AZD0530 (e.g., greater hypotensive effects) and that they should be used with greater caution and possible dose reduction.

Initiation of prohibited medications during the course of the study should be discouraged, however, if an excluded medication is initiated after screening, the site should consult with the Project Director and/or Medical Monitor for further guidance.

Investigational agents are prohibited one month prior to entry and for the duration of the trial. Also exclusionary is previous treatment with an investigational small molecule with anti-amyloid properties or passive immunization against amyloid within 1 year of entry or previous treatment with an active immunization against amyloid.

Particular attention should be paid to AST and ALT and clinical symptoms of rhabdomyolysis in subjects taking lovastatin or simvastatin.

Current use of warfarin or other anticoagulants are exclusionary for CSF substudy only.

6.10.2 Permitted Concomitant Medications

Gabapentin and pregabulin for non-seizure indications are permitted.

Use of short- to medium-acting benzodiazepines for treatment for insomnia or anxiety is permitted. Use of sedatives or hypnotics should be avoided for 8 hours before administration of cognitive tests unless they are given chronically.

7.0 STUDY POPULATION

The study will enroll males and females age 55-85 with mild Alzheimer's disease as specified in the entry criteria below. Subjects who do not meet all inclusion criteria, disease diagnostic criteria, or who meet any exclusion criteria may not be randomized into the study without prior approval from the Project Director (and/or Medical Monitor). Efforts will be made to target at least 20% minority enrollment, which will be facilitated through special minority outreach effort coordinated by the Coordinating Center.

7.1 Inclusion Criteria

Subjects must meet the following criteria:

1. NIA-Alzheimer's Association core clinical criteria for probable AD[73]
2. ^{18}F-Florbetapir scan with evidence of elevated Aβ (based on central review)
3. Age between 55-85 (inclusive)
4. MMSE[81] score between 18 and 26 (inclusive)
5. Stability of permitted medications for 4 weeks (see sections 6.9 and 6.10.2 for further details). In particular:
    - Stable doses of antidepressants lacking significant anticholinergic side effects (if they are not currently depressed and do not have a history of major depression within the past 1 year)
    - Cholinesterase inhibitors and memantine are allowable if stable for 12 weeks prior to screen
6. Geriatric Depression Scale[94] less than 6 [Note: a score ≥6 on this screening scale may be permissible, if the subject is examined by a site clinician and judged not to be depressed.]
7. Study partner is available who has frequent contact with the subject (e.g., average of 10 hours per week or more), and can accompany the subject to most visits to answer questions about the subject
8. Visual and auditory acuity adequate for neuropsychological testing
9. Good general health with no disease expected to interfere with the study
10. Subject is not pregnant, lactating, or of childbearing potential (i.e., women must be two years post-menopausal or surgically sterile)
11. Modified Hackinski[95] less than or equal to 4
12. Completed six grades of education or has a good work history
13. Must speak English or Spanish fluently

7.2 Exclusion Criteria

Subjects must not meet the following criteria:

1. Any significant neurologic disease other than AD, such as Parkinson's disease, multi-infarct dementia, Huntington's disease, normal pressure hydrocephalus, brain tumor, progressive supranuclear palsy, seizure disorder, subdural hematoma, multiple sclerosis, or history of significant head trauma followed by persistent neurologic defaults or known structural brain abnormalities

2. Screening/baseline MRI scan with evidence of infection, infarction, or other focal lesions or multiple lacunes or lacunes in a critical memory structure
3. Subjects that have any contraindications for MRI studies, including claustrophobia, the presence of metal (ferromagnetic) implants, or cardiac pacemaker
4. Major depression, bipolar disorder as described in DSM-IV within the past 1 year or psychotic features, agitation or behavioral problems within 3 months, which could lead to difficulty complying with the protocol
5. History of schizophrenia (DSM V criteria)
6. History of alcohol or substance abuse or dependence within the past 2 years (DSM V criteria)
7. Clinically significant or unstable medical condition, including uncontrolled hypertension, uncontrolled diabetes, or significant cardiac, pulmonary, renal, hepatic, endocrine, or other systemic disease in the opinion of the Investigator, may either put the subject at risk because of participation in the study, or influence the results, or the subject's ability to participate in the study.
8. Has had a history within the last 5 years of a primary or recurrent malignant disease with the exception of non-melanoma skin cancers, resected cutaneous squamous cell carcinoma in situ, basal cell carcinoma, cervical carcinoma in situ, or in situ prostate cancer with normal prostate-specific antigen post-treatment
9. Clinically significant abnormalities in B12 or TFTs that might interfere with the study. A low B12 is exclusionary, unless follow-up labs (homocysteine (HC) and methylmalonic acid (MMA)) indicate that it is not physiologically significant.
10. Residence in skilled nursing facility.
11. Use of any excluded medication as described in section 6.10.1.
12. Current or recent participation in any procedures involving radioactive agents, including current, past, or anticipated exposure to radiation in the workplace, such that the total radiation dose exposure to the subject in a given year would exceed the limits of annual and total dose commitment set forth in the US Code of Federal Regulations (CFR) Title 21 Section 361.1. This guideline is an effective dose of 5 rem received per year.
13. Neutropenia defined as absolute neutrophils count of <1,800/microliter
14. Thrombocytopenia defined as platelet count <120x103/microliter
15. For CSF sub-study participants, a current blood clotting or bleeding disorder, or significantly abnormal PT or PTT at screening
16. Clinically significant abnormalities in screening laboratories, including:
    - Aspartate aminotransferase (AST) >1.5 times ULN
    - Alanine aminotransferase (ALT) > 1.5 times ULN
    - Total bilirubin >1.5 times ULN
    - Serum creatinine >2.0 times ULN
17. History of interstitial lung disease
18. Patients whom the PI deems to be otherwise ineligible

7.3 Recruitment and Retention Strategies

Study-wide recruitment efforts will be overseen by a recruitment team at the Coordinating Center, which has developed a coordinated recruitment plan to ensure that enrollment occurs in a timely fashion. The overall goals of the plan are to raise awareness of the trial among targeted populations to ensure adequate enrollment. The Coordinating Center partners with NIA and coordinates with its ADEAR Center to leverage existing resources. The recruitment and retention team will develop materials specific to the AZD0530 trial for use by the sites, provide ongoing recruitment assistance and support, and develop tracking procedures to monitor effectiveness of recruitment efforts. The recruitment plan for the AZD0530 trial will include both the utilization of participants existing at clinical trial centers and the recruitment of additional populations by methods of outreach.

8.0 STUDY TIMELINE

The approximate timeline for this study is projected as follows: 1) approximately 6 months for study startup activities that may include FDA/IND submission, site IRB and regulatory approvals, and the Investigator training meeting; and 2) approximately 2 years for screening and treatment period, and analysis.

9.0 DESCRIPTION OF STUDY VISITS

9.1 Pre-screening

During the prescreen phase, sites will identify potential participants through a variety of mechanisms (i.e. reviewing patients enrolled in ADCs, de novo recruitment, and referrals) and assess if they may potentially qualify for the study following the site's standard practice.

9.2 Screening

Up to 60 days will be permitted to allow for completion of all Screening procedures, assessment of eligibility criteria, and completion of all Baseline procedures, including randomization. The Screening procedures will typically require 3 visits to complete.

Initial Screening (Visit 1a):

The purpose of this visit is to determine initial study eligibility. Potential participants, or legally authorized representatives, and their study partners must sign an informed consent form prior to administration of any study-related procedures. Information regarding the subject's demographics, concurrent medications, and medical history will be gathered along with cognitive, clinical, and safety assessments. For a complete list of all Visit 1a procedures, refer to the Schedule of Events (Appendix 1). Procedures for Visit 1a may be completed over multiple days.

All Visit 1a procedures must be completed and all information related to eligibility, including screening lab results, must be reviewed by the investigator or qualified designee for assessment of the subject's eligibility before the subject can proceed to the screening MRI scan (Visit 1b).

MRI (Visit 1b):

The MRI will be read locally to confirm eligibility. The site clinician should review the local read for exclusionary findings prior to completion of the ^{18}F-Florbetapir PET scan. If there are no exclusionary findings on the MRI the subject will continue on to the ^{18}F-Florbetapir PET scan.

^{18} F-Florbetapir PET (Visit 1c):

The ^{18} F-Florbetapir PET scan will be read centrally to confirm eligibility. If the ^{18} F-Florbetapir PET scan shows evidence of elevated Aβ (per inclusion criteria), the subject may continue to baseline following clinical monitor review and verification that all eligibility requirements have been met. If the PET scan does not show evidence of elevated Aβ, then the subject will be excluded.

9.2.1 Re-screens

Unless otherwise approved by the Project Director, only one re-screen is allowed, should the original screen be a failure. The re-screen should typically occur at least 3 months after the original screen failure. Individuals who fail screening due to ineligible results on the amyloid PET scan may not be re-screened.

9.3 Baseline Visit (Week 0)

The Baseline visit may only be initiated following completion of all Screening assessments and required approvals. The Baseline visit procedures may be completed over multiple days and will typically require at least two (2) visits. All Baseline procedures, including randomization must be completed within 60 days of initiation of Screening Visit procedures.

Baseline procedures include cognitive, functional, and behavioral assessments, safety assessments, review of concurrent medications and adverse events, ^{18}F-FDG PET and biofluid collection (blood, plasma, and optional CSF). For a complete list of all Baseline procedures, refer to the Schedule of Events (Appendix 1).

Baseline cognitive assessments for CSF sub-study subjects must be done prior to, or at least 48 hours after, the lumbar puncture. If done on the same day, Baseline cognitive assessments must be done prior to the ^{18}F-FDG PET scan. In no instance should cognitive assessments be performed while the subject is in a fasted state.

For further information regarding specific visit procedures, refer to section 12.

Following completion of all Baseline procedures and confirmation that the Baseline FDG PET has passed central QC, participants who continue to meet all protocol inclusion criteria and no exclusion criteria, will be randomized and dispensed study medication. If holding randomization for the return of FDG QC results were to unduly burden a participant, e.g. due to lengthy travel, the site may consult with the Project Director (and/or Medical Monitor) for guidance and a possible exception.

9.4 Treatment Visits (Week 2 – Week 52)

Evaluations of safety and tolerability, clinical and biomarker assessments, and study drug dispensing will be performed throughout the treatment period. For a complete list of all visits and visit procedures, refer to the Schedule of Events (Appendix 1).

Timing of each follow-up visit is calculated from the Baseline visit date. Procedures for each visit may be completed over multiple days, but must occur within the window for that visit. Visit window for each visit are as follows:

**Visit / Target Date**	**Visit Window**
**Week 2**	10-21 days from baseline
**Week 4**	21-35 days from baseline<br>Note: there must be sufficient time between weeks 2 and 4 for drug level data to be obtained
**Weeks 6 and 8**	+/- 1 week from target date
**Other visits**	+/- 2 weeks from target date

Plasma drug level measured at week 2 will determine study drug allocation that will be dispensed at week 4 as described in Section 6.2.

At Week 52, the MRI, ^{18}F-FDG PET scan, and lumbar puncture (for CSF sub-study participants) should occur during the 2 weeks prior to the in-clinic visit (while subject remains on study medication). Cognitive assessments for CSF sub-study subjects must be done at least 48 hours after the lumbar puncture. If done on the same day, cognitive assessments must be done prior to the ^{18}F-FDG PET scan. In no instance should cognitive assessments be performed while the subject is in a fasted state.

CSF sub-study participants who did not have a successful CSF collection at Baseline should still attempt a collection at Week 52.

For further information regarding specific visit procedures, refer to section 12.

10.0 EARLY TREATMENT/STUDY DISCONTINUATION

The investigators at each site will make every reasonable effort to maximize participant retention. However, if an investigator removes a participant from treatment or study, or if a participant declines further treatment or study participation, an Early Discontinuation Visit will be completed as soon as possible following discontinuation of the study drug and or at time of study discontinuation.

The Early Discontinuation Visit will contain the same assessments as the week 52 visit, to allow collection of the main outcome measures. Depending on when the last study visit was conducted, certain procedures (e.g. LP, MRI or FDG PET scan) may not be required at the Early Discontinuation Visit. Refer to the Procedures Manual for further guidance. If an in-person visit is not possible, site personnel will complete as much of the Early Discontinuation Visit as possible by telephone. All early treatment discontinuation participants will be strongly encouraged to complete all remaining study visits.

Replacement participants are not required for those who discontinue from treatment or study (i.e. an additional participant does not need to enroll in the study for every participant discontinued).

10.1 Reasons for Early Discontinuation

AZD0530 has been associated with potentially serious neutropenia and thrombocytopenia, although this has not been seen in healthy volunteers with doses up to 250 mg daily. While we do not expect to see these adverse events with the doses of AZD0530 in this study, the laboratory criteria for considering drug discontinuation are as follows:

a. Neutropenia defined as absolute neutrophil count of <1,500/microliter
b. Thrombocytopenia defined as platelet count <100X103/microliter
c. Interstitial lung disease by high resolution chest CT, attributed to drug exposure. The following algorithm will be used:

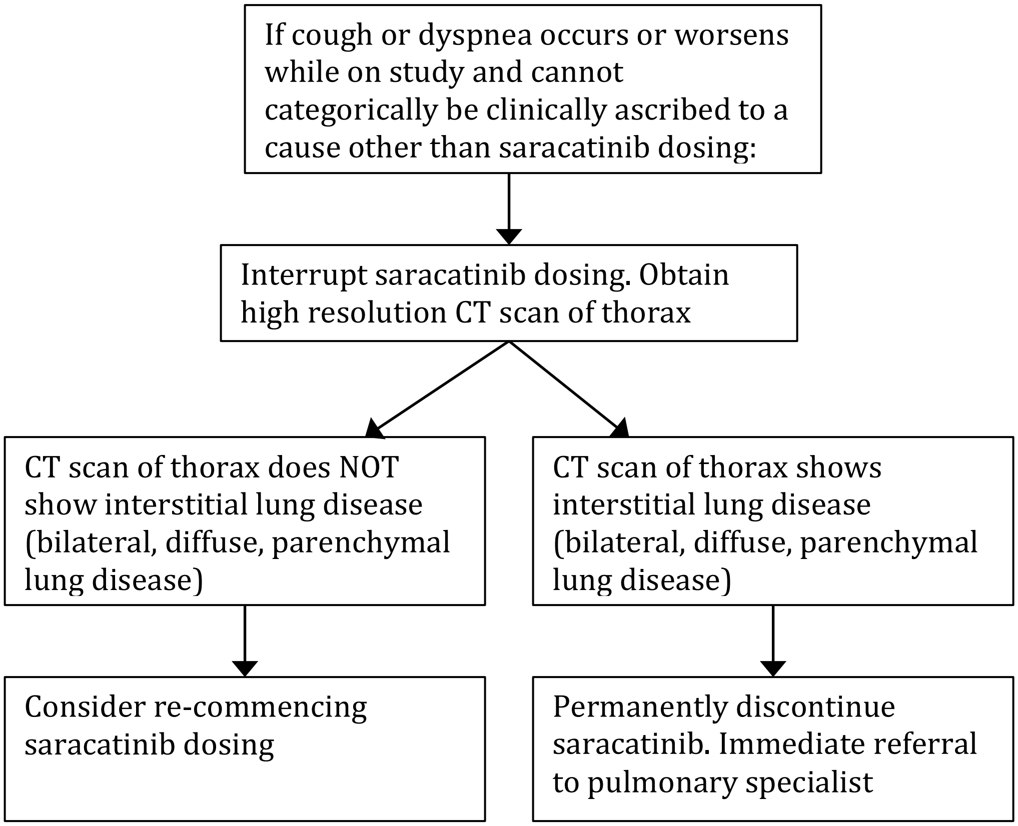

If the laboratory criteria for drug discontinuation due to neutropenia or thrombocytopenia as described above are triggered, study drug should be held and labs should be repeated (locally or centrally) as soon as possible for confirmation of the findings. If findings are confirmed, the participant should be permanently discontinued from study drug. If the study physician thinks that discontinuation of study drug is not warranted he/she should consult with the Project Director and/or Medical Monitor for final guidance.

Other abnormal laboratory values will be evaluated individually. The clinical criterion for discontinuation is defined as a single Serious (or intolerable) Adverse Event that the site clinician or the DSMB consider related to study medication. Clinical symptoms and decisions regarding drug discontinuation will be reviewed and made on an individual basis by the study physician, in collaboration with the Project Director, Medical Monitor, and DSMB as needed.

Participants may also be discontinued from treatment/study for reasons such as the following:

- Adverse experience: The participant has experienced an adverse event that, in the opinion of the investigator, requires early termination. This may include abnormal laboratory values.
- Death
- Safety risk: Any participant who becomes a safety risk during the trial will be withdrawn.
- Protocol violation: The participant fails to meet protocol entry criteria or did not adhere to protocol requirements.
- Non-compliance: The participant is non-compliant with completion of study-related evaluations and or intake of study drugs.
- In the investigator's judgment, it is in the participant's best interest to discontinue participation in the treatment/study.
- Consent is withdrawn. The participant wishes to withdraw from the study, or the legally authorized representative wish the participant to be withdrawn.
- The study is terminated by the Project Director/Coordinating Center, alone or at the recommendation of the Data Safety Monitoring Board.
- Lost to follow up. Participant could not be recalled back to conduct follow up visits.
- Loss of informed study partner. The participant no longer has a responsible study partner to oversee participant visits and administration of study drug.
- Coordinating Center Request. The coordinating center determines it is in the participant's best interest to discontinue participation from the treatment and or study.

11.0 INSTRUMENTS

11.1 Cognitive Evaluations

11.1.1 Alzheimer's Disease Assessment Scale-Cognitive (ADAS-Cog12)

The ADAS-Cog[82] is a structured scale that evaluates memory (word recall, word recognition), reasoning (following commands), language (naming, comprehension), orientation, ideational praxis (placing letter in envelope) and constructional praxis (copying geometric designs). Ratings of spoken language, language comprehension, word finding difficulty, and ability to remember test instructions are also obtained. The test is scored in terms of errors, with higher scores reflecting poorer performance and greater impairment. For secondary analyses, the ADAS-cog 11 (which excludes the 10-point Delayed Word Recall item assessing recall of the learned word list[83]) will be used with scores ranging from 0 (best) to 70 (worse).

11.1.2 Mini-Mental State Examinations (MMSE)

The MMSE[81] is a brief, frequently used screening instrument for Alzheimer's disease drug studies. The MMSE scale evaluates orientation, memory, attention, concentration, naming, repetition, comprehension, and ability to create a sentence and to copy two overlapping pentagons. The MMSE is scored as the number of correctly completed items with a lower score indicative of poorer performance and

greater cognitive impairment. The total score ranges from 0 (worse) to 30 (perfect performance).

11.2 Clinical and Functional Evaluations

11.2.1 ADCS-AD Activities of Daily Living (ADCS-ADL-Standard)

The Alzheimer's Disease Cooperative Study - Activities of Daily Living Scale (ADCS-ADL) is an activities of daily living questionnaire aimed at detecting functional decline in people with mild to moderate Alzheimer's Disease. It was developed as a result of a research project funded by the National Institute on Aging (NIA) and conducted by the ADCS to develop cognitive and functional instruments for the assessment of patients with AD with relevance to clinical trials[84]. The ADCS-ADL was selected to best cover the assessment of patients with mild to moderate dementia due to AD, and has been used widely in clinical trials where patients have baseline MMSE scores in a range of 16-24/30 [43, 93]. Questions are administered to a qualified study partner about a set of 6 basic and 17 instrumental ADL (23 ADL items are covered overall). Performance of each of these activities during the past 4 weeks, as well as the level of performance, are rated. A total score is derived by summing scores across items, and ranges from 0 (maximal impairment) to 78 (maximally independent function).

11.2.3 Geriatric Depression Scale

The Geriatric Depression Scale (Short Form) is a self-report scale designed to screen for symptoms of depression in the elderly[94]. The scale consists of 15 questions that the participant is asked to answer yes or no on the basis of how they felt over the past week. The more benign items are asked first. Answers to 5 of the items are negatively oriented for depression (e.g., Do you feel full of energy?) and 10 positively oriented (e.g., Do you often feel helpless?). One point is given for each appropriate positive or negative answer indicative of a symptom of depression, for a possible total of 15 points. Total scores of 0-5 are considered likely to be normal and scores of 6-15 are considered to be more likely to be depressed.

11.2.4 Neuropsychiatric Inventory (NPI)

The NPI is a well-validated, reliable, multi-item instrument to assess psychopathology in AD based on interview with the study partner[87, 88]. The NPI evaluates both the frequency and severity of 10 (or 12) neuropsychiatric features, including delusions, hallucinations, agitation/aggression, dysphoria, anxiety, euphoria, apathy, disinhibition, irritability/lability, and aberrant motor behavior, as well as evaluates sleep and appetite/eating disorders. Frequency assessments range from 1 (occasionally, less than once per week) to 4 (very frequently, once or more per day or continuously) as well as severity (1=mild, 2=moderate, 3=severe). The score for each subscale is the product of severity and frequency and the total score is the sum of all subscales. The 12-item NPI will be used in this study.

11.2.5 Modified Hachinski

The Modified Hachinski is a brief questionnaire that incorporates questions about medical history, cognitive symptoms and features of stroke, reported by a study partner as well as the neurological examination and neuroimaging studies[95].

11.2.6 Research Satisfaction Survey

The Research Satisfaction Survey (RSS) was adapted from the work of Larsen et al[96] to measures the satisfaction of elderly volunteers in clinical trials to treat or prevent cognitive loss or dementia. The RSS builds on the Client Satisfaction Questionnaire (CSQ-8) which demonstrates a high degree of validity and internal consistency and measured consumer satisfaction with health and human service programs. Higher scores on the CSQ indicate a greater level of satisfaction in service programs[98]; conversely, lower CSQ-8 scores have been associated with higher dropout rates[98] and missed appointments in treatment settings[97] and in urban Community Mental Health program[96]. Open-ended questions about satisfaction are part of the CSQ, and allow participants to provide feedback about specific operational procedures of a particular service program[96]. The RSS is similarly designed with a 7- item survey and may include additional items to inquire about the specific aspects of a given trial. Four items are designed to be answered with a modified likert scale and can be used to create a summary score with a range of 4-16, which can be analyzed with parametric statistics; three items provide for open ended responses about the intervention, the assessments and the frequency of visits and may be used to understand retention rates.

11.2.7 Treatment Blinding Questionnaire

The Treatment Blinding Questionnaire assesses the perception of blind being maintained until the end of the study. The questionnaire is to be completed by the study participant, study partner, and principal investigator at the end of study.

12.0 PROCEDURES

12.1 Safety Assessments

12.1.1 Physical and Neurological Examination

A medically qualified professional will perform a brief physical examination that consists of a review of the major body systems (I.E. skin, head/ears/eyes/nose/throat (HEENT), cardiovascular, pulmonary, abdomen, musculoskeletal, and extremities). Assessments of height, weight, and vital signs (systolic and diastolic blood pressure, pulse, temperature, oxygen saturation, and respiration) are included. Neurological examination will include an assessment of cranial nerves, strength, coordination, reflexes, sensation, tremor, gait and mental status.

12.1.2 Electrocardiogram (ECG)

An appropriately qualified individual will conduct a standard 12-lead resting ECG. The ECG report must be reviewed, signed, and dated by the site Principal Investigator (or a medically-qualified individual delegated by the PI). Those with clinically significant ECG findings will be referred for follow-up as deemed appropriate by the investigator and may be excluded from the study.

12.1.3 Clinical Laboratory Evaluations

All routine laboratory samples will be analyzed by a central laboratory, which will provide a procedures manual and supplies. Lab reports will be reviewed, signed and dated by the site Principal Investigator (or a medically-qualified individual delegated by the PI). If a value is outside of the laboratory's normal range, the clinician will indicate if it is clinically significant or not. Those results that are deemed clinically significant may need to be repeated and follow up with the patient's primary care physician for further evaluation should occur.

For results that are deemed possibly related to study medication, the site investigator and/or the Medical Monitor and the Project Director may request additional unscheduled laboratory tests or may temporarily or permanently discontinue study medication. Study drug will always be temporarily held for laboratory-related AEs deemed "severe" (corresponding to CTCAE grade 3 or higher). Refer to Section 10.1 for laboratory criteria for drug discontinuation.

12.2 Biofluids

12.2.1 CSF Substudy

Subjects will have the option to participate in the CSF Sub-study.

All samples will be collected after an 8 hour fast, preferably in the morning. Site personnel should advise the subject not to take study medication on the morning of the procedure. Efforts should be made to conduct the follow-up LP at the same time of day as the Baseline LP.

Prior to lumbar puncture (LP), a coagulation panel will be obtained to rule out a clotting disorder. Subjects taking an anti-platelet agent (e.g. aspirin) may, at the discretion of the site clinician, be discontinued from that agent for a period of time prior to lumbar puncture and/or continue off agent for a period of time post LP. Participants who are taking anticoagulants (e.g. warfarin (Coumadin) and/or dabigatran (Pradaxa)) may not undergo an LP and are not suitable to participate in this substudy.

When done at the same visit, the LP must always occur after (or a minimum of 72 hours prior to) the MRI scan. LP under fluoroscopy is permitted, if needed. Site personnel should advise the subject that use of fluoroscopy (x-rays) involves exposure to radiation.

A total volume of 20 mL of CSF should be collected for each LP. To clear blood associated with needle insertion, the first 1-2 mL (or more if needed) of CSF should be discarded. 1-2 mL CSF (or volume per local laboratory requirements) will be sent to the local laboratory for protein, glucose, and cell count. Remaining CSF will be frozen on dry ice or in a -80 freezer and shipped overnight frozen to the ADCS Biomarker Core at UCSD for processing and analysis. Analyses will include AD biomarkers (e.g. Aβ and tau, exploratory biomarkers), and, at week 52, analysis of

drug levels. Samples will also be frozen and banked at the ADCS Biomarker Core at UCSD and Strittmatter laboratory at Yale for future analysis.

Each study participant or a person designated to speak for them will be contacted by phone one day after the LP to confirm participant well being and to query about any adverse events.

12.2.2 Plasma for Biomarkers

Plasma samples will be collected at Baseline and at Week 52 for analysis of AD biomarkers (e.g. Aβ40 & 42, exploratory biomarkers).

Blood for plasma collection should be collected in a uniform fashion into 10ml lavender top EDTA tubes (provided by Coordinating Center). Once blood is collected, it is shipped overnight at ambient temperature to the ADCS Biomarker Core at UCSD for processing and analysis.

Blood samples will be processed for plasma by the ADCS Biomarker Core and stored at UCSD and the Strittmatter laboratory at Yale for future analysis.

12.2.3 Plasma for AZD0530 Levels

AZD0530 plasma levels will be analyzed at select visits as described in the Schedule of Events (Appendix 1). It is crucial that site personnel advise subjects not to take study medication on the morning of the collection.

For participants in the CSF sub-study, at week 52, plasma for AZD0530 levels should be collected as close as possible to the time of the LP to allow for comparisons of drug levels in plasma and CSF.

Plasma is collected as described above in 12.2.2 using lavender top EDTA tubes. Once blood is collected into a 10 mL EDTA plastic tube, it is shipped overnight at ambient temperature to the ADCS Biomarker Core at UCSD for analysis.

12.2.4 APOE Genotype and other DNA Markers

DNA will be extracted from participant blood samples at the Baseline visit and will be analyzed for APOE genotype. This will allow secondary analyses of data on the impact of the APOE genotype on putative biomarkers of AD, clinical outcome measures, and adverse events (Aim 6). APOE polymorphisms are an important genetic risk factor for AD. Individuals with one or two APOE4 alleles have a higher risk of developing AD and an earlier age of onset. Furthermore, those with one or two APOE4 alleles exhibit a more rapid rate of clinical change in some, but not all studies.

Blood is collected in a uniform fashion using EDTA as anti-coagulant, as described in section 12.2.2. Once blood is collected into a 10 mL EDTA plastic tube, it is shipped overnight at ambient temperature to the ADCS Biomarker Core at UCSD for APOE genotyping. Participants will be asked to consent to optional DNA banking for future

research studies. The DNA will be banked at the ADCS Biomarker Core at UCSD and Strittmatter laboratory at Yale.

12.3 Magnetic Resonance Imaging (MRI)

Subjects will have a brain MRI scan as part of the screening evaluation, as well as at Week 52 to enable volumetric MRI analyses as a secondary biomarker of AD progression (Aim 4). Measurement will rely on nonlinear registration between baseline and follow-up images to calculate point-by-point volumetric change[68], along with FreeSurfer-based probabilistic-atlas image segmentation to identify and calculate average change across regions of interest (ROIs) as defined in the Desikan et al. brain atlas[69]. The entirety of the Desikan atlas will be available for post-hoc exploratory analyses, but secondary outcome measures will be limited to the regions noted in the study aims to reduce planned comparisons.

12.3.1 Site Qualification

Each site's scanner will have an identifying number, which will be appended to the image header of all MRI data. Each scanner will be subjected to a pre-qualifying process that includes an evaluation for excessive vibration or other image artifact revealed through submitted MR images of an American College of Radiology phantom obtained by applying the study's standardized protocol. If the scanner has not been used previously in an applicable trial, then an additional qualification step will require the scanning of a human volunteer. If the scanner was previously used in an applicable trial, the qualification process can often be shortened and a human volunteer may not be needed, except in special circumstances. Sites must demonstrate the ability to follow the imaging protocol as prescribed in the MRI procedures manual and to provide 3D T1 MPRAGE or IR-FSPGR images using the procedures for submitting scans for automated segmentation. The resulting segmentations will be examined centrally to assess suitability for quantitative analysis. Because longitudinal analysis of brain volumetry will be used in this study, sites will be required to use the same qualified scanner for all scans of a particular subject.

12.3.2 Data Acquisition

When done at the same visit, the MRI scan must always occur prior to (or at least 72 hours after) an LP.

Briefly, the protocol for image acquisition will include a brief localizer scan, followed by a high-resolution 3D T1 structural series (MPRAGE or IR-SPGR), a T2-weighted series (FLAIR), a diffusion weighted scan and a gradient recalled echo scan. The volumetric analysis procedure will include corrections for gradient nonlinearities[70] and intensity non-uniformity[71, 72] using methods developed within the Morphometry Biomedical Informatics Research Network (mBIRN; http://www.birncommunity.org/). Analysis procedures will be repeated for follow-up scans.

The MRI will be performed using a 1.5 or 3.0 Tesla magnet. The MRI will take approximately 30 minutes to complete. Injectable contrast agents will not be used.

Conscious sedation can be used in rare cases if deemed appropriate by the site clinician. Refer to section 6.10.2 for details of timing of administration of sedating medications relative to cognitive testing.

12.3.3 Clinical Read of MRIs

Each site will be required to procure a local clinical read of each subject's MRI data by qualified personnel. At screening, this local read should be used to ensure that subjects do not meet exclusion criteria as described in section 7.2.

12.3.4 Data Management and Quality Control

MRI data will be uploaded to the secure study data portal. All imaging data will be de-identified through a numerical coding system. Each MRI will be assessed centrally for scan quality allowing volumetric analysis. Quality control for MRI will result in failure of some scans which may need to be repeated. Repeat scans must be scheduled within four weeks of the visit date.

12.4 PET Imaging

12.4.1 Site Qualification

Each site must be qualified for PET. If the machine being used has already been certified by the ADNI PET Core and has not experienced any major software upgrades, re-qualification will not be required. Qualification of the PET scanner applies to both the ^{18}F-FDG PET and ^{18}F-Florbetapir imaging protocols. Qualification will employ the same methods utilized for site qualification in ADNI. Sites will be provided with a Hoffman brain phantom (if one is not available to the site) and a technical manual for the data acquisition using ^{18}F, generally FDG. The phantom must be scanned on two sequential days using the protocol identical to that required for human imaging. This enables the University of Michigan to ascertain the characteristics of the scanner (particularly resolution and uniformity) and assure that sites are capable of performing the protocol for acquisition and image reconstruction. All phantom images will be provided to the University of Michigan for review and qualification.

12.4.2 ^{18}F-Florbetapir PET Data Acquisition

Conscious sedation for PET imaging is not allowable.

PET data will be acquired from study participants using ADNI protocols: a 20-min dynamic emission scan will be performed, consisting of four 5-min frames in the 3D mode 50 minutes after an intravenous administration of 10 mCi of ^{18}F-Florbetapir, either preceded by a CT scan (for PET/CT scanners) or followed by a transmission scan (for PET-only scanners). Data will be corrected for radiation-attenuation and scanner using transmission scans or X-ray CT, and reconstructed using reconstruction algorithms specified for each type of scanner as described in the PET technologist's manual. ^{18}F-Florbetapir PET data will be transferred to the study data portal to be retrieved by the team at Banner Alzheimer's Institute imaging facility for central review and analysis following quality control checks and standardization by University of Michigan. Eligibility for the study will be determined based on both visual and quantitative metrics for elevated amyloid burden. Visual readings from

the raw images will be based on recently validated algorithms[76] and guidelines provided in the Amyvid package insert[77]. The final determination regarding PET scan eligibility will be made available via the study data portal for the site investigator and coordinator to access.

12.4.3 ^{18}F-FDG PET Data Acquisition

Conscious sedation for PET imaging is not allowable.

^{18}F-FDG PET scans will be acquired according to a standardized protocol. Participants will be instructed to fast for at least four hours prior to a scan. A 30-min dynamic emission scan, consisting of six 5-min frames, either preceded by a CT scan (for PET/CT scanners) or followed by a transmission scan (for PET-only scanners), will be acquired starting 30 minutes after the intravenous injection of 5 mCi of ^{18}F-FDG, as the subject lies quietly in a dimly lit room with their eyes open and with minimal sensory stimulation. Data will be corrected for radiation-attenuation and scatter using transmission scans or X-ray CT, and reconstructed using reconstruction algorithms specified for each type of scanner as described in the PET technologist's manual.

12.4.4 ^{18}F-Florbetapir and ^{18}F-FDG PET Data Management and Quality Control

The University of Michigan under the direction of Robert A. Koeppe, PhD will assess each PET scan for scan quality. Quality control for PET will result in failure of some scans which may need to be reprocessed or repeated. Repeat scans must be scheduled within four (4) weeks of the visit date.

All raw and processed image data required to reconstruct the PET images (including the normalization, CT, transmission, and blank scans must be archived. It is necessary to archive and store raw and processed data at the imaging site for the duration of the project.

Each acquired PET dataset will be reviewed and pre-processed using standardized procedures to identify artifacts and minimize scanner-dependent differences in ^{18}F-Florbetapir or ^{18}F-FDG uptake. During the pre-processing, automated algorithms will be used to register and average each subject's six (FDG) or four (Florbetapir) 5-min emission frames, transform each registered image into a 160×160×1.5 mm voxel matrix with sections parallel to a horizontal section through the anterior and posterior commissures (without any adjustment for size or shape), normalize the images for individual variations in absolute image intensity, and apply a filter function previously customized for each scanner using a Hoffmann brain phantom scanned during the site qualification process to ensure an isotropic spatial resolution of 8 mm full-width-at-half-maximum (FWHM).

13.0 PERSONNEL REQUIREMENTS

The Protocol Principal Investigator (PI) is responsible for the overall conduct of the study at the site. The PI is to supervise project personnel and ensure that clinical raters maintain a high level of skill and accuracy in conducting assessments.

Additionally, the PI will perform or supervise clinical evaluation of all participants and ensure protocol adherence. Additional key personnel may be required, as specified in the procedures manual.

14.0 ADVERSE EVENTS

14.1 Definition

An adverse event (AE) is defined as per the Code of Federal Regulation Title 21 Part 312:
http://www.accessdata.fda.gov/scripts/cdrh/cfdocs/cfcfr/cfrsearch.cfm?fr=312.32

Adverse events which occur after informed consent is signed and up to 30 days after the study drug has been discontinued, include but are not limited to: (1) worsening or change in nature, severity, or frequency of conditions or symptoms present at the start of the study; (2) participant deterioration due to primary illness; (3) intercurrent illness; and (4) drug interaction. An abnormal laboratory value will only be reported as an AE if the investigator considers it to be clinically significant, or if it leads to the participant being withdrawn from the study.

The investigator should attempt to establish a diagnosis of the event based on signs, symptoms, and or other clinical information. In such cases, the diagnosis should be documented as the AE and not the individual signs or symptoms. Symptoms and conditions present at the beginning of the study will be characterized, so that AEs can be defined as any new symptom, or any increase in frequency or severity of an existing symptom. Adverse events should be described with medical terminology so that the event can be matched against a medical coding dictionary, such as MedDRA (Medical Dictionary for Regulatory Activities).

Investigators should report their assessment of the potential relatedness of each AE to the protocol procedure(s) and also to the investigational product. Attribution of AEs will be rated as definite, probable, possible, unlikely or unrelated.

Following questioning and evaluation, all AEs, whether determined to be related or unrelated to the study drug by a medically qualified site PI or clinician must be documented in the participant's records, in accordance with the investigator's normal clinical practice, and on the AE eCRF.

14.2 Following Up on AEs

The investigator is obliged to follow participants with AEs until the events have subsided, the conditions are considered medically stable, or the participants are no longer available for follow up. Participants who discontinue due to adverse experiences will be treated and followed according to established medical practice. All pertinent information will be entered into the eCRF. All adverse events will be reported to the independent Data Safety Monitoring Board (DSMB). Adverse events will be rated as mild, moderate or severe.

15.0 SERIOUS ADVERSE EVENTS (SAE)

15.1 Definition

A serious adverse event is defined as per the Code of Federal Regulation Title 21 Part 312:
http://www.accessdata.fda.gov/scripts/cdrh/cfdocs/cfcfr/cfrsearch.cfm?fr=312.32

Medical and scientific judgment should be exercised in deciding whether reporting a serious adverse event is appropriate in other situations, such as important medical events that may not be immediately life-threatening or result in death or hospitalization but may jeopardize the subject or may require intervention to prevent one of the outcomes listed in the code of Federal Regulations Title 21 Part 312.

Hospitalizations that fulfill one of the following conditions will not have to be reported as SAE:

- Admission for treatment of a pre-existing condition that is not associated with the development of a new AE or with a worsening of the pre-existing condition (i.e., work-up for persistent lab abnormality that occurred prior to the study)
- Social admission (i.e., participant has no place to sleep)
- Administrative admission (i.e., yearly physical exam)
- Protocol-specified admission (i.e., for a procedure required by the study protocol)
- Optional admission not associated with a precipitating clinical AE (i.e., pre-planned treatments, elective cosmetic surgery)

15.2 Reporting SAEs

Any serious adverse event which occurs during the course of the investigation (i.e. anytime after informed consent, regardless of study drug exposure) or within 30 days of receiving the last dose or last study visit must be reported to the Project Director and the Coordinating Center within 24 hours of learning of the event. This in turn will trigger an alert to the appropriate Coordinating Center personnel and Protocol Project Director, which will lead to the initiation of the creation of the report. A notification will be sent to all participating sites and the DSMB once the report is available. Sites will inform their IRB of the event based on local IRB requirements. The Coordinating Center will also report unanticipated problems and SAEs to the NCATS program official, NCATS Medical Monitor(s), and to the FDA – IND holder, Dr. Stephen Strittmatter.

The local site investigator and research staff will monitor the study procedures for this trial for overall safety and scientific relevance on an ongoing basis. The local site PI (in conjunction with, as needed, the Medical Monitor, the Project Director, and the DSMB) will evaluate every adverse event for safety and causality, and will determine

whether the adverse event affects the Risk/Benefit ratio of the study and whether modifications to the protocol or consent form are required.

The Office for Human Research Protections considers unanticipated problems involving risks to subjects or others to include, in general, any incident, experience, or outcome that meets all of the following criteria:

- unexpected in terms of nature, severity, or frequency given (a) the research procedures that are described in the protocol-related documents, such as the IRB approved research protocol and informed consent document; and (b) the characteristics of the subject population being studied
- related or possibly related to participation in the research ("possibly related" means there is a reasonable possibility that the incident, experience, or outcome may have been caused by the procedures involved in the research);
- suggests that the research places subjects or others at a greater risk of harm (including physical, psychological, economic, or social harm) than was previously known or recognized.

ATRI will manage these unanticipated problems as SUSARs that involve the project director and DSMB or as AEs of Medical Interest, which would be reported in local IRB reports from sites.

16.0 DATA AND SAFETY MONITORING BOARD (DSMB)

The DSMB is an independent advisory group to the Coordinating Center Director, the Project Director, and the NCATS. No investigator involved in the trial is a member of the DSMB.

The initial task of the DSMB will be to review the protocol to identify any necessary modifications. If modifications are necessary, revisions will be reviewed by the DSMB prior to its recommendation on initiation of the project. The DSMB, based on its review of the protocol, will work with the Coordinating Center personnel to identify the study-specific data parameters and format of the information to be regularly reported. The DSMB will initially be provided with data blinded to treatment status, but they may request unblinded data if there is a safety concern. The DSMB and NIA representative will meet in person or by conference call on a quarterly basis.

Additionally, the DSMB will be informed of the occurrence of any serious adverse events within 7 days of being reported to the Coordinating Center. The DSMB may at any time request additional information from the Coordinating Center.

Based on the review of safety data, the DSMB will make recommendations regarding the conduct of the study. These may include amending safety monitoring procedures, modifying the protocol or consent, terminating the study or continuing the study as designed. Using the Safety Review Process and the DSMB described above, there is substantial oversight and case review to alert the investigators, in a

timely manner, to any safety issues that may arise. For further details please refer to the DSMB charter.

17.0 RECORDING AND COLLECTION OF DATA

17.1 Case Report Form

The Principal Investigator or designee will record all data collected (either written or electronic record of data). Written or electronic data of record must be entered on the electronic Case Report Form (eCRF) provided for that purpose. In some instances no prior written or electronic record of data may exist and data recorded directly on the eCRF is considered source data. The site will be suitably trained on the use of the eCRF and appropriate site personnel will be authorized to provide electronic signatures. The Principal Investigator is responsible to verify the integrity of the data and acknowledge as such by signature.

All site entries will be made in a secured web site and the Principal Investigator will review the record for completeness. If corrections are necessary to the eCRFs, the Principal Investigator or designee will update the eCRF and provide documentation for the reason for change.

Completed eCRFs will be submitted according to the Coordinating Center's instructions, and reviewed by the Coordinating Center to determine their acceptability. If necessary, data correction requests will be generated for resolution by the study site.

17.2 Study Files and Patient Source Documents

Patient confidentiality is strictly held in trust by the participating investigators and research staff. This confidentiality is extended to cover testing of biological samples and genetic tests in addition to the clinical information relating to participants.

The study protocol, documentation, data and all other information generated will be held in strict confidence. No information concerning the study or the data will be released to any unauthorized third party, without prior written approval of the sponsoring institution. Authorized representatives of the sponsoring institution may inspect all documents and records required to be maintained by the Investigator, including but not limited to, medical records (office, clinic or hospital) and pharmacy records for the participants in this study. The clinical study site will permit access to such records. Any data, specimens, forms, reports, and other records that leave the site will be identified only by a participant identification number (Participant ID, PID) to maintain confidentiality. All records will be kept in a locked file cabinet. All computer entry and networking programs will be done using PIDs only. Information will not be released without written permission of the participant, except as necessary for monitoring by IRB, the FDA, the NIA, and the OHRP.

Information about study participants will be kept confidential and managed according to the requirements of the Health Insurance Portability and Accountability Act of 1996 (HIPAA). Those regulations require a signed participant HIPAA Authorization informing the participant of the following:

- What protected health information (PHI) will be collected from participants in this study
- Who will have access to that information and why
- Who will use or disclose that information
- The rights of a research participant to revoke their authorization for use of their PHI.

In the event that a participant revokes authorization to collect or use PHI, the investigator, by regulation, retains the ability to use all information collected prior to the revocation of participant authorization. Each site PI, under the guidance of his/her IRB, is responsible for ensuring that all applicable HIPAA regulations and State laws are met.

18.0 ETHICS AND REGULATORY CONSIDERATIONS

18.1 Good Clinical Practice

This study will be conducted in compliance with the protocol and accordance with Good Clinical Practice (GCP) guidelines, as defined by the International Conference on Harmonisation (ICH) Guideline, Topic E6, the United States Code of Federal Regulations, Title 21, Part 50 (21CFR50) – Protection of Human Subjects and Part 56 – Institutional Review Boards (IRBs), HIPAA, State and Federal regulations and all other applicable local regulatory requirements and laws.

Study personnel involved in conducting this study will be qualified by education, training and experience to perform their respective task(s) in accordance with GCP.

No study document shall be destroyed without prior written agreement between the Coordinating Center and the investigator. Should the investigator wish to assign study records to another party or move them to another location, he/she may do so only with the prior written consent of the Coordinating Center.

18.2 Institutional Review Board (IRB) / Research Ethics Boards (REB)

Institutional Review Boards and Research Ethics Boards must be constituted and their authority delegated through the institution's normal process of governance according to applicable State and Federal requirements for each participating location. Each participating institution must provide for the review and approval of this protocol and the associated informed consent documents and recruitment material by an appropriate IRB registered with the Office for Human Research Protections (OHRP). Any amendments to the protocol or consent materials must also be approved before they are placed into use. In the United States, only

institutions holding a current US Federalwide Assurance issued by OHRP may participate. Refer to: http://www.hhs.gov/ohrp/assurances/.

The investigator must obtain approval from the IRB for all subsequent protocol amendments and, when warranted, changes to the informed consent document and recruitment materials. Protocol and informed consent form amendments can be made only with the prior approval of the Coordinating Center. The investigator may not implement any protocol deviation without prior notification to the Coordinating Center and prior review and documented approval of the IRB, except where necessary to eliminate an immediate hazard to study participants, or when change(s) involve only logistical or administrative aspects of the trial (ICH 4.5.4). The investigator shall notify the IRB of deviations from the protocol or serious adverse events occurring at the site, in accordance with local procedures.

18.3 Informed Consent and HIPAA Compliance

Informed consent will be obtained in accordance with US 21 CFR 50.25, the Tri-Council Policy Statement: Ethical Conduct of Research Involving Humans and the Health Canada and ICH Good Clinical Practice. Applicable HIPAA privacy notifications will be implemented and HIPAA authorizations signed before protocol procedures are carried out. Information should be given in both oral and written form as deemed appropriate by the Site's IRB.

Prior to the beginning of the trial, the investigator should have the IRB's written approval of the written informed consent form and any other written information to be provided to participants and be acceptable by Regulatory Affairs at the Coordinating Center. Consent forms must be in a language fully comprehensible to the prospective participants and/or their authorized representatives and study partners. Participants, their relatives, guardians, or authorized representatives and study partners will be given ample opportunity to inquire about the details of the study. Prior to a participant's participation in the trial, the written informed consent form should be signed and personally dated by the participant or by the participant's legally acceptable representative, and by the person who conducted the informed consent discussion. Participants should be provided a copy of the signed ICF.

The informed consent will not only cover consent for the trial itself, but for the genetic samples/data/storage, biomarker samples/data/storage, and imaging scans/data/storage as well. Consent forms will specify that DNA and biomarker samples are for research purposes only; the tests on the DNA and biomarker samples are not diagnostic in nature and participants will never receive results.

18.4 Genetic Research and Storage of Genetic Material

The DNA is banked in locked -80 degree freezers in the ADCS Biomarker Core laboratory at UCSD and in the Strittmatter laboratory at Yale University. Sample tubes are bar-coded and linked to participant ID number only and banked without

personal identifiers. Samples are recorded into a computerized inventory database that is encrypted and password-protected.

Only DNA from consenting participants will be banked and used to facilitate future research on aging and dementia, particularly in the discovery of genetic polymorphisms that may influence risk of developing AD. Collection of DNA will permit qualified investigators to probe candidate genetic polymorphisms as predictors of outcome in future studies. The samples will be stored by the ADCS as long as funding is available from the NIH. If funding should lapse completely, the UCSD ADRC will provide responsible custodianship of the ADCS Biomarker Core.

18.5 Storage of Biospecimen Samples

All biospecimens being banked for future AD biomarker research will be shipped to and stored by the ADCS Biomarker Core at UCSD and the Strittmatter laboratory at Yale University. Sample tubes are bar-coded and linked to participant ID number only and banked without any personal identifiers. Samples are tracked by a computerized inventory database that is encrypted and password-protected at UCSD. Similarly, at Yale, sample records will be computerized, locked, and password protected.

Only biospecimens from consenting participants will be banked and used to facilitate future research on aging and dementia. The samples will be stored by the ADCS as long as funding is available from the NIH. If funding should lapse completely, the UCSD ADRC will provide responsible custodianship of the ADCS Biomarker Core.

18.6 MRI and PET Imaging and Data Storage

MRI and PET scans will be labeled according to each site's imaging machine capabilities using Subject ID and scanner specific series descriptions as detailed in the Technical Manual. All imaging data will be de-identified using participant identifiers as detailed in the Technical Manual and checked centrally to confirm the absence of participant identifying information. MRI scan findings of clinical significance, as determined by the local clinical read and the site PI, may be shared with the study participant and the participant's local physician.

18.7 Inclusion of Children as Participants

Children will not be included.

18.8 Study Monitoring

The clinical monitor is responsible for inspecting the electronic case report forms and source documentation at regular intervals throughout the study to verify adherence to the protocol, completeness and accuracy of the data, and adherence to local regulations on the conduct of clinical research. The monitoring visits must be conducted according to the applicable ICH and GCP guidelines to ensure protocol adherence, quality of data, drug accountability, compliance with regulatory requirements and continued adequacy of the investigational site and its facilities.

The Site Investigator will cooperate in the monitoring process by ensuring the availability of the eCRFs, source documents and other necessary documents at the time of the monitoring visits. Site Investigator will promptly address any matters brought to his/her attention by the monitor. The Site Investigator may also be asked to meet in-person with the site monitor during certain visits.

19.0 AUDIT

In accordance with ICH GCP, representatives of the Coordinating Center and/or regulatory agency may select this study for audit. The Investigator and study staff are responsible for maintaining the site master file containing all study-related regulatory documentation as outlined by Regulatory Affairs that will be suitable for inspection at any time by the Coordinating Center, its designees, and/or regulatory agencies. Inspection of site facilities (e.g., pharmacy, laboratories) to evaluate the trial conduct and compliance with the protocol may also occur.

20.0 RECORD RETENTION

Essential documents and study records must be retained for a minimum of seven years following primary publication of study results. The Coordinating Center will notify sites when retention of such documents is no longer required.

21.0 PUBLICATION POLICY

The results of this study will be published. To coordinate dissemination of data from this study, a publication committee will be formed. The committee will consist of Dr. van Dyck, Dr. Strittmatter, interested Principal Investigators and appropriate Coordinating Center personnel. The committee will solicit input and assistance from other Investigators as appropriate and adhere to all Coordinating Center Publications Policies.

22.0 SHARING OF FINAL RESEARCH DATA

Data from this research will be shared with other researchers pursuant to the 02/26/2003 “NIH Final Statement on Sharing Research Data”. The NIH policy can be found at:
http://grants.nih.gov/grants/guide/notice-files/NOT-OD-03-032.html

NIH believes that data sharing is important for further translation of research results into knowledge, products, and procedures to improve human health. The NIH endorses the sharing of final research data to serve these and other important scientific goals. To protect participants’ rights and confidentiality, identifiers will be removed from the data before they are shared.

23.0 LITERATURE CITED

1. Gonzalez-Maeso, J., et al. Identification of a serotonin/glutamate receptor complex implicated in psychosis. Nature 452, 93-97 (2008).
2. Grant, S.G., et al. Impaired long-term potentiation, spatial learning, and hippocampal development in fyn mutant mice. Science 258, 1903-1910 (1992).
3. Alzheimer's, A. 2012 Alzheimer's disease facts and figures. Alzheimers Dement 8, 131-168 (2012).
4. Um, J.W., et al. Alzheimer amyloid-beta oligomer bound to postsynaptic prion protein activates Fyn to impair neurons. Nat Neurosci 15, 1227-1235 (2012).
5. Bhaskar, K., Hobbs, G.A., Yen, S.H. & Lee, G. Tyrosine phosphorylation of tau accompanies disease progression in transgenic mouse models of tauopathy. Neuropathol Appl Neurobiol 36, 462-477 (2010).
6. Bhaskar, K., Yen, S.H. & Lee, G. Disease-related modifications in tau affect the interaction between Fyn and tau. J Biol Chem 280, 35119-35125 (2005).
7. Lee, G., et al. Phosphorylation of tau by fyn: implications for Alzheimer's disease. J Neurosci 24, 2304¬2312 (2004).
8. Lee, G., Newman, S.T., Gard, D.L., Band, H. & Panchamoorthy, G. Tau interacts with src-family non-receptor tyrosine kinases. J Cell Sci 111 (Pt 21), 3167-3177 (1998).
9. Ittner, L.M., et al. Dendritic function of tau mediates amyloid-beta toxicity in Alzheimer's disease mouse models. Cell 142, 387-397 (2010).
10. Holtzman, D.M., Morris, J.C. & Goate, A.M. Alzheimer's disease: the challenge of the second century. Science translational medicine 3, 77sr71 (2011).
11. Shaw, L.M., et al. Cerebrospinal fluid biomarker signature in Alzheimer's disease neuroimaging initiative subjects. Ann Neurol 65, 403-413 (2009).
12. Jack, C.R., Jr., et al. Hypothetical model of dynamic biomarkers of the Alzheimer's pathological cascade. Lancet Neurol 9, 119-128 (2010).
13. Lesne, S., et al. A specific amyloid-beta protein assembly in the brain impairs memory. Nature 440, 352-357 (2006).
14. Shankar, G.M., et al. Amyloid-beta protein dimers isolated directly from Alzheimer's brains impair synaptic plasticity and memory. Nat Med 14, 837-842 (2008).
15. Walsh, D.M., et al. Naturally secreted oligomers of amyloid beta protein potently inhibit hippocampal long-term potentiation in vivo. Nature 416, 535-539 (2002).
16. Lambert, M.P., et al. Diffusible, nonfibrillar ligands derived from Abeta1-42 are potent central nervous system neurotoxins. Proc Natl Acad Sci U S A 95, 6448-6453 (1998).
17. Thomas, S.M. & Brugge, J.S. Cellular functions regulated by Src family kinases. Annual review of cell and developmental biology 13, 513-609 (1997).
18. Hunter, T. A tail of two src's: Mutatis mutandis. Cell 49, 1-4 (1987).
19. Nakazawa, T., et al. Characterization of Fyn-mediated tyrosine phosphorylation sites on GluR epsilon 2 (NR2B) subunit of the N-methyl-D-aspartate receptor. J Biol Chem 276, 693-699 (2001).
20. Kojima, N., Ishibashi, H., Obata, K. & Kandel, E.R. Higher seizure susceptibility and enhanced tyrosine phosphorylation of N-methyl-D-aspartate receptor subunit 2B in fyn transgenic mice. Learning & memory (Cold Spring Harbor, N.Y 5, 429-445 (1998).
21. Prybylowski, K., et al. The synaptic localization of NR2B-containing NMDA receptors is controlled by interactions with PDZ proteins and AP-2. Neuron 47, 845-857 (2005).

22. Suzuki, T. & Okumura-Noji, K. NMDA receptor subunits epsilon 1 (NR2A) and epsilon 2 (NR2B) are substrates for Fyn in the postsynaptic density fraction isolated from the rat brain. Biochem Biophys Res Commun 216, 582-588 (1995).
23. Liang, S., et al. Neuroprotective profile of novel SRC kinase inhibitors in rodent models of cerebral ischemia. J Pharmacol Exp Ther 331, 827-835 (2009).
24. Lauren, J., Gimbel, D.A., Nygaard, H.B., Gilbert, J.W. & Strittmatter, S.M. Cellular prion protein mediates impairment of synaptic plasticity by amyloid-beta oligomers. Nature 457, 1128-1132 (2009).
25. Chen, S., Yadav, S.P. & Surewicz, W.K. Interaction between human prion protein and amyloid-beta (Abeta) oligomers: role OF N-terminal residues. J Biol Chem 285, 26377-26383 (2010).
26. Calella, A.M., et al. Prion protein and Abeta-related synaptic toxicity impairment. EMBO Mol Med 2, 306-314 (2010).
27. Balducci, C., et al. Synthetic amyloid-beta oligomers impair long-term memory independently of cellular prion protein. Proc Natl Acad Sci U S A 107, 2295-2300 (2010).
28. Kessels, H.W., Nguyen, L.N., Nabavi, S. & Malinow, R. The prion protein as a receptor for amyloid-beta. Nature 466, E3-4; discussion E4-5 (2010).
29. Cisse, M., et al. Ablation of cellular prion protein does not ameliorate abnormal neural network activity or cognitive dysfunction in the J20 line of human amyloid precursor protein transgenic mice. J Neurosci 31, 10427-10431 (2011).
30. Resenberger, U.K., et al. The cellular prion protein mediates neurotoxic signaling of beta-sheet-rich conformers independent of prion replication. Embo J 30, 2057-2070 (2011).
31. Bate, C. & Williams, A. Amyloid-beta-induced synapse damage is mediated via cross-linkage of cellular prion proteins. J Biol Chem 286, 37955-37963 (2011).
32. Alier, K., Ma, L., Yang, J., Westaway, D. & Jhamandas, J.H. Abeta Inhibition of Ionic Conductance in Mouse Basal Forebrain Neurons Is Dependent upon the Cellular Prion Protein PrPC. J Neurosci 31, 16292-16297 (2011).
33. Chung, E., et al. Anti-PrPC monoclonal antibody infusion as a novel treatment for cognitive deficits in an alzheimer's disease model mouse. BMC Neurosci 11, 130 (2010).
34. Gimbel, D.A., et al. Memory impairment in transgenic Alzheimer mice requires cellular prion protein. J Neurosci 30, 6367-6374 (2010).
35. Kudo, W., et al. Cellular prion protein is essential for oligomeric amyloid-beta-induced neuronal cell death. Hum Mol Genet 21, 1138-1144 (2012).
36. You, H., et al. Abeta neurotoxicity depends on interactions between copper ions, prion protein, and N-methyl-D-aspartate receptors. Proc Natl Acad Sci USA 109, 1737-1742 (2012).
37. Zou, W.Q., et al. Amyloid-{beta}42 Interacts Mainly with Insoluble Prion Protein in the Alzheimer Brain. J Biol Chem 286, 15095-15105 (2011).
38. Freir, D.B., et al. Interaction between prion protein and toxic amyloid beta assemblies can be therapeutically targeted at multiple sites. Nat Commun 2, 336 (2011).
39. Barry, A.E., et al. Alzheimer's disease brain-derived amyloid-beta-mediated inhibition of LTP in vivo is prevented by immunotargeting cellular prion protein. J Neurosci 31, 7259-7263 (2011).

40. Bizat, N., et al. Neuron dysfunction is induced by prion protein with an insertional mutation via a Fyn kinase and reversed by sirtuin activation in Caenorhabditis elegans. J Neurosci 30, 5394-5403 (2010).
41. Malaga-Trillo, E., et al. Regulation of embryonic cell adhesion by the prion protein. PLoS Biol 7, e55 (2009).
42. Chin, J., et al. Fyn kinase induces synaptic and cognitive impairments in a transgenic mouse model of Alzheimer's disease. J Neurosci 25, 9694-9703 (2005).
43. Quinn JF, Raman R, Thomas RG, Yurko-Mauro K, Nelson EB, Van Dyck C, Galvin JE, Emond J, Jack CR Jr, Weiner M, Shinto L, Aisen PS. Docosahexaenoic acid supplementation and cognitive decline in Alzheimer disease: a randomized trial. JAMA. 2010 Nov 3;304(17):1903-11 (2010).
44. Larson, M., et al. The Complex PrPc-Fyn Couples Human Oligomeric Abeta with Pathological Tau Changes in Alzheimer's Disease. J Neurosci 32, 16857-16871 (2012).
45. Salter, M.W. & Kalia, L.V. Src kinases: a hub for NMDA receptor regulation. Nat Rev Neurosci 5, 317-328 (2004).
46. Scheff, S.W., DeKosky, S.T. & Price, D.A. Quantitative assessment of cortical synaptic density in Alzheimer's disease. Neurobiol Aging 11, 29-37 (1990).
47. Lacor, P.N., et al. Abeta oligomer-induced aberrations in synapse composition, shape, and density provide a molecular basis for loss of connectivity in Alzheimer's disease. J Neurosci 27, 796-807 (2007).
48. Shankar, G.M., et al. Natural oligomers of the Alzheimer amyloid-beta protein induce reversible synapse loss by modulating an NMDA-type glutamate receptor-dependent signaling pathway. J Neurosci 27, 2866-2875 (2007).
49. Roberson, E.D., et al. Amyloid-beta/Fyn-induced synaptic, network, and cognitive impairments depend on tau levels in multiple mouse models of Alzheimer's disease. J Neurosci 31, 700-711 (2011).
50. Chin, J., et al. Fyn kinase modulates synaptotoxicity, but not aberrant sprouting, in human amyloid precursor protein transgenic mice. J Neurosci 24, 4692-4697 (2004).
51. Cruchaga, C., et al. SNPs associated with cerebrospinal fluid phospho-tau levels influence rate of decline in Alzheimer's disease. PLoS Genet 6(2010).
52. Bekris, L.M., et al. Tau phosphorylation pathway genes and cerebrospinal fluid tau levels in Alzheimer's disease. American journal of medical genetics. Part B, Neuropsychiatric genetics : the official publication of the International Society of Psychiatric Genetics 159B, 874-883 (2012).
53. Hennequin, L.F., et al. N-(5-chloro-1,3-benzodioxol-4-yl)-7-[2-(4-methylpiperazin-1-yl)ethoxy]-5-(tetrahydro-2H-pyran-4-yloxy)quinazolin-4-amine, a novel, highly selective, orally available, dual-specific c-Src/Abl kinase inhibitor. J Med Chem 49, 6465-6488 (2006).
54. Gangadhar, T.C., Clark, J.I., Karrison, T. & Gajewski, T.F. Phase II study of the Src kinase inhibitor saracatinib (AZD0530) in metastatic melanoma. Invest New Drugs (2012).
55. Gucalp, A., et al. Phase II trial of saracatinib (AZD0530), an oral SRC-inhibitor for the treatment of patients with hormone receptor-negative metastatic breast cancer. Clinical breast cancer 11, 306-311 (2011).
56. Mackay, H.J., et al. A phase II trial of the Src kinase inhibitor saracatinib (AZD0530) in patients with metastatic or locally advanced gastric or gastro esophageal junction

(GEJ) adenocarcinoma: a trial of the PMH phase II consortium. Invest New Drugs 30, 1158-1163 (2012).
57. Fury, M.G., et al. Phase II study of saracatinib (AZD0530) for patients with recurrent or metastatic head and neck squamous cell carcinoma (HNSCC). Anticancer Res 31, 249-253 (2011).
58. Renouf, D.J., et al. A phase I/II study of the Src inhibitor saracatinib (AZD0530) in combination with gemcitabine in advanced pancreatic cancer. Invest New Drugs 30, 779-786 (2012).
59. Reiman, E.M. & Langbaum, J.B.S. Brain imaging in the evaluation of putative Alzheimer's disease slowing, risk-reducing and prevention therapies. in Imaging the Aging Brain (eds. Jagust, W.J. & D'Esposito, M.) 319-350 (Oxford University Press, New York, 2009).
60. Alexander, G.E., Chen, K., Pietrini, P., Rapoport, S.I. & Reiman, E.M. Longitudinal PET Evaluation of Cerebral Metabolic Decline in Dementia: A Potential Outcome Measure in Alzheimer's Disease Treatment Studies. Am J Psychiatry 159, 738-745 (2002).
61. Reiman, E.M., et al. Declining brain activity in cognitively normal apolipoprotein E epsilon 4 heterozygotes: A foundation for using positron emission tomography to efficiently test treatments to prevent Alzheimer's disease. Proc Natl Acad Sci USA 98, 3334-3339 (2001).
62. Landau, S.M., et al. Associations between cognitive, functional, and FDG-PET measures of decline in AD and MCI. Neurobiol Aging 32, 1207-1218 (2011).
63. Chen, K., et al. Twelve-month metabolic declines in probable Alzheimer's disease and amnestic mild cognitive impairment assessed using an empirically pre-defined statistical region-of-interest: findings from the Alzheimer's Disease Neuroimaging Initiative. NeuroImage 51, 654¬664 (2010).
64. Atiya, M., Hyman, B.T., Albert, M.S. & Killiany, R. Structural magnetic resonance imaging in established and prodromal Alzheimer disease: a review. Alzheimer Dis Assoc Disord 17, 177-195 (2003).
65. Jack, C.R., Jr., et al. Medial temporal atrophy on MRI in normal aging and very mild Alzheimer's disease. Neurology 49, 786-794 (1997).
66. Jack, C.R., Jr., et al. Antemortem MRI findings correlate with hippocampal neuropathology in typical aging and dementia. Neurology 58, 750-757 (2002).
67. Knight, W.D., et al. Acceleration of cortical thinning in familial Alzheimer's disease. Neurobiol Aging 32, 1765-1773 (2011).
68. Holland, D., et al. Subregional neuroanatomical change as a biomarker for Alzheimer's disease. Proc Natl Acad Sci U S A 106, 20954-20959 (2009).
69. Desikan, R.S., et al. An automated labeling system for subdividing the human cerebral cortex on MRI scans into gyral based regions of interest. NeuroImage 31, 968-980 (2006).
70. Jovicich, J., et al. Reliability in multi-site structural MRI studies: effects of gradient non-linearity correction on phantom and human data. NeuroImage 30, 436-443 (2006).
71. Sled, J.G., Zijdenbos, A.P. & Evans, A.C. A nonparametric method for automatic correction of intensity nonuniformity in MRI data. IEEE Trans Med Imaging 17, 87-97 (1998).

72. Arnold, J.B., et al. Qualitative and quantitative evaluation of six algorithms for correcting intensity nonuniformity effects. NeuroImage 13, 931-943 (2001).
73. McKhann, G.M., et al. The diagnosis of dementia due to Alzheimer's disease: recommendations from the National Institute on Aging-Alzheimer's Association workgroups on diagnostic guidelines for Alzheimer's disease. Alzheimers Dement 7, 263-269 (2011).
74. Clark, C.M., et al. Use of florbetapir-PET for imaging beta-amyloid pathology. JAMA 305, 275-283 (2011).
75. Landau, S.M., et al. Amyloid deposition, hypometabolism, and longitudinal cognitive decline. Ann Neurol 72, 578-586 (2012).
76. Mintun, M., et al. Evaluation of a binary read methodology for florbetapir-PET images. in Human Amyloid Imaging (Miami, FL, 2012).
77. Eli Lilly and Company. Amyvid [package insert]. (Lilly USA, LLC, Indianapolis, IN, 2012).
78. de Leon, M.J., et al. MRI and CSF studies in the early diagnosis of Alzheimer's disease. J Intern Med 256, 205-223 (2004).
79. Grossman, M., et al. Cerebrospinal fluid profile in frontotemporal dementia and Alzheimer's disease. Ann Neurol 57, 721-729 (2005).
80. Gilman, S., et al. Clinical effects of Abeta immunization (AN1792) in patients with AD in an interrupted trial. Neurology 64, 1553-1562 (2005).
81. Folstein, M.F., Folstein, S.E. & McHugh, P.R. "Mini-mental state". A practical method for grading the cognitive state of patients for the clinician. Journal of psychiatric research 12, 189-198 (1975).
82. Rosen, W.G., Mohs, R.C. & Davis, K.L. A new rating scale for Alzheimer's disease. Am J Psychiatry 141, 1356-1364 (1984).
83. Mohs, R.C., et al. Development of cognitive instruments for use in clinical trials of antidementia drugs: additions to the Alzheimer's Disease Assessment Scale that broaden its scope. The Alzheimer's Disease Cooperative Study. Alzheimer Dis Assoc Disord 11 Suppl 2, S13-21 (1997).
84. Galasko, D., Bennett, D., Sano, M., Ernesto, C., Thomas, R., Grundman, M., Ferris, S. An inventory to assess activities of daily living for clinical trials in Alzheimer's disease. The Alzheimer's Disease Cooperative Study. Alzheimer Dis. Assoc. Disord. 11, S33-39 (1997).
85. Morris, J.C. The Clinical Dementia Rating (CDR): current version and scoring rules. Neurology 43, 2412-2414 (1993).
86. Hughes, C.P., Berg, L., Danziger, W.L., Coben, L.A. & Martin, R.L. A new clinical scale for the staging of dementia. Br J Psychiatry 140, 566-572 (1982).
87. Cummings, J.L. The Neuropsychiatric Inventory: assessing psychopathology in dementia patients. Neurology 48, S10-16 (1997).
88. Cummings, J.L., et al. The Neuropsychiatric Inventory: Comprehensive assessment of psychopathology in dementia. Neurology 44, 2308-2314 (1994).
89. Dalton, R.N., Chetty, R., Stuart, M., Iacona, R.B. & Swaisland, A. Effects of the Src inhibitor saracatinib (AZD0530) on renal function in healthy subjects. Anticancer Res 30, 2935-2942 (2010).
90. Hannon, R.A., et al. Effects of Src kinase inhibition by saracatinib (AZD0530) on bone turnover in advanced malignancy in a Phase I study. Bone 50, 885-892 (2012).

91. Baselga, J., et al. Phase I safety, pharmacokinetics, and inhibition of SRC activity study of saracatinib in patients with solid tumors. Clinical cancer research: an official journal of the American Association for Cancer Research 16, 4876-4883 (2010).
92. Fujisaka, Y., et al. First report of the safety, tolerability, and pharmacokinetics of the Src kinase inhibitor saracatinib (AZD0530) in Japanese patients with advanced solid tumours. Invest New Drugs (2012).
93. Sano M, Bell KL, Galasko D, Galvin JE, Thomas RG, van Dyck CH, Aisen PS. A randomized, double-blind, placebo-controlled trial of simvastatin to treat Alzheimer disease. Neurology. 2011 Aug 9;77(6):556-63 (2011).
94. Sheikh, J. & Yesavage, J. Geriatric Depression Scale (GDS): Recent evidence and development of a shorter version. in Clinical gerontology: A guide to assessment and intervention (ed. Brind, T.L.) 165-173 (Haworth Press, New York, 1986).
95. Rosen, W.G., Terry, R.D., Fuld, P.A., Katzman, R. & Peck, A. Pathological verification of ischemic score in differentiation of dementias. Ann. Neurol. 7, 486-488 (1980).
96. Larsen, D.L., Attkisson, C.C., Hargreaves W.A., & Nguyen T.D. Assessment of client/patient satisfaction: development of a general scale. Eval Program Plann., 2(3), 197-207 (1979).
97. Larsen, D.L., Nguyen T.D., Green R.S., & Attkisson, C.C. Enhancing the utilization of outpatient mental health services. Community Mental Health J., 19(4), 305-320 (1983).
98. Lunnen, K.M., & Ogles B.M. A multiperspective, multivariable evaluation of reliable change. J Consult Clin Psychol., 66(2), 400-410 (1998).

APPENDIX 1 SCHEDULE OF EVENTS

**Visit Number**	**1**	**2**	**3**	**4**	**5**	**6**	**7**	**8**	**9**	**10**	**11**	**12**	**13**
**Visit Name (Week)**[a]		0	2	4	6	8	13	19	26	32	39	45	52[f]
**Visit Name**	Screening	Baseline											
Informed Consent	X												
Demographics	X												
Medical History	X												
Geriatric Depression Scale	X												
MMSE	X						X		X		X		X
ADAS-Cog		X					X		X		X		X
ADCS-ADL		X							X				X
CDR-SB		X							X				X
NPI		X							X				X
Physical Examination	X			X		X	X	X	X	X	X	X	X
Neurological Examination	X			X		X	X	X	X	X	X	X	X
Modified Hachinski	X												
Vital Signs[b]	X	X	X	X	X	X	X	X	X	X	X	X	X
Height	X												
ECG	X												X
Venipuncture	X	X	X	X	X	X	X	X	X	X	X	X	X
Safety Labs [c]	X[d]		X	X	X	X	X	X	X	X	X	X[d]	X
Urinalysis	X												
APOE Genotyping		X											
DNA Banking		X											
AZD0530 plasma level			X				X		X		X		X
AD Biomarkers (plasma)		X											X
MRI	X												X[e]
Florbetapir PET	X												
FDG PET		X											X[e]
LP/CSF AD Biomarkers (optional)		X											X[e]
AZD0530 CSF level (optional)													X[e]
Adverse Events	X	X	X	X	X	X	X	X	X	X	X	X	X
Medication Compliance			X	X	X	X	X	X	X	X	X	X	X
Concomitant Medications	X	X	X	X	X	X	X	X	X	X	X	X	X
Dispense Study Medication		X		X			X		X		X		
Research Satisfaction Survey		X							X				X
Treatment Blinding Questionnaire													X

[a] See section 9.4 for definition of visit windows.
[b] Vital signs will include sitting blood pressure, pulse, temperature, respiration rate, weight, and oxygen saturation
[c] Screening labs include: Hematology, Chemistry Panel, TSH, Vitamin B12. Follow-Up labs include: Hematology and Chemistry Panel
[d] At Screening and Week 45, PT/PTT will also be collected for patients in the CSF sub-study.
[e] These assessments will occur two weeks prior to the week 52 visit; 7 consecutive days of study medication compliance prior to the week 52 LP are necessary for a valid CSF drug level.
[f] The Early Discontinuation Visit will contain the same assessments as the week 52 visit.

APPENDIX 2 REVISION HISTORY

Protocol Version	Summary of Changes
**v2.0**<br>**10-AUG-2015**	• Changed Coordinating Center from ADCS to ATRI throughout<br>• Corrected typographical errors<br>• Added Appendix 2 – Revision History
**v3.0**<br>**31-MAY-2016**	• Updated Medical Monitor name and contact information. (Cover Page, Signature Page)<br>• Revised timeframe for completion of all screening and baseline procedures to 60 days. (Synopsis, Sections 9.2 and 9.3)<br>• Added language consistent with IB regarding avoiding conception during participation in the trial (Section 4.1.2)<br>• Corrected modified intent-to-treat definition to reflect inclusion of randomized participants with a valid baseline and post-baseline measurement. (Section 5.3)<br>• Added study sponsor, NCATS to list of entities that could terminate the trial and recipients of reports regarding SAEs and unanticipated problems. (Sections 5.6 and 15.2)<br>• Clarified that any designated personnel may access study drug. (Section 6.5)<br>• Added focused period of compliance assessment in the period leading up to the final study LP. (Section 6.7 and Appendix 1)<br>• Added exceptions to some excluded medication categories. (Section 6.10.1)<br>• Revised entry criteria for exclusionary neutropenia and thrombocytopenia. (Section 7.2)<br>• Added requirement to hold randomization until the Baseline FDG PET has passed central QC. (Section 9.3)<br>• Clarified procedures to follow and considerations to be made if the laboratory criteria for discontinuation are triggered. (Section 10.1)<br>• Revised requirement about discontinuation of anti-platelet meds pre/post LP – that this should left to the discretion of the site clinician. (Section 12.2.1)<br>• Clarified that sedation is not allowed for PET imaging. (Sections 12.4.2 and 12.4.3)<br>• Added language clarifying the Office for Human Research Protections definition of "unanticipated problems" (Section 15.2)<br>• Clarified that biospecimen samples are only banked for those who consent to banking. (Section 18.5)<br>• Clarified that CSF drug levels will be analyzed at the final visit for LP sub-study participants. (Appendix 1)<br>• Other formatting and grammatical corrections. (Throughout)
**v4.0**<br>**31-MAY-2017**	• Incorporated revisions to Section 5 and to description of secondary analysis (changed from ADAS-cog12 to ADAS-Cog11) to be consistent with the Statistical Analysis Plan<br>• Corrected acronym use for CDR-Sum of Boxes.<br>• Updated ATRI address

Keck School of Medicine of USC

Alzheimer's Therapeutic Research Institute

CONFIDENTIAL DOCUMENT

STATISTICAL ANALYSIS PLAN v1.0

A Phase 2a Multi-Center Study of F-FDG PET, Safety, and Tolerability of AZD0530 in Mild Alzheimer's Disease

April 20, 2017
Author: Yanxin Jiang, MS
From the ATRI Biostatistics Team

Contents

1 Introduction 3

2 Abbreviations 3

3 Study Design/Summary 4
3.1 Overview . . . 4
3.2 Study Aims . . . 4
3.2.1 Primary Aims . . . 4
3.2.2 Secondary Aims . . . 4
3.3 Study Population . . . 4
3.4 Power and Sample Size Determination . . . 4
3.5 Treatment Allocation . . . 5

4 Study Outcome Variables 6
4.1 Primary Outcome . . . 6
4.2 Secondary Outcomes . . . 6
4.3 Safety Outcomes . . . 6
4.4 Biomarker and Imaging Outcomes . . . 6

5 Sequence of Planned Analysis 6
5.1 Interim Analysis . . . 6
5.2 Final Analyses and Reporting . . . 6

6 Statistical Methods 7
6.1 Analysis Populations . . . 7
6.2 Multiple-testing Strategy . . . 7
6.3 Incomplete Follow-up/ Missing Data . . . 7
6.4 Covariates . . . 7
6.5 Data Management and Analysis Software . . . 7

7 Statistical Analysis 8
7.1 Study Flow . . . 8
7.2 Patient characteristics and baseline variables . . . 8
7.3 Analysis of Primary Outcome . . . 8
7.4 Analysis of Secondary Outcomes . . . 9
7.5 Analysis of Biomarker and Imaging Outcomes . . . 9
7.6 Exploratory Subgroup Analyses . . . 10
7.6.1 Quartiles of Drug Exposure . . . 10

8 Approvals 11

9 Revision History 11

CONFIDENTIALITY STATEMENT: This report from the USC Alzheimer's Therapeutic Research Institute (ATRI) is for the sole use of the intended recipient(s) and contains confidential and privileged information. Any unauthorized review, use, disclosure, copying or distribution is strictly prohibited.

1 Introduction

This Statistical Analysis Plan (SAP) describes the planned analyses and reporting for the study, *A Phase 2a Multi-Center Study of F-FDG PET, Safety, and Tolerability of AZD0530 in Mild Alzheimer's Disease* (FYN).

The planned analyses identified in this SAP, will be included in future manuscripts. Exploratory analyses not identified in this SAP may be performed to support planned analyses. Any post-hoc or unplanned analyses not specified in this SAP will be clearly identified as such in published manuscripts.

The following documents were reviewed when preparing this SAP:

- FYN Clinical Research Protocol (`FYN Protocol v3.0 - 20160531 - clean.pdf`)
- Case report forms (CRFs) for FYN
- ATRI Statistical Analysis Plan SOP (`ATRI SOP BI-002 Statistical Analysis Plan v1.0 20150908.pdf`)

Readers of this SAP should also read the Clinical Research Protocol for details on the conduct of this study and the operational aspects of clinical assessments and timing for completing a patient in this study.

2 Abbreviations

ABBREVIATION	DEFINITION
ADAS-Cog	Alzheimer's Disease Assessment Scale-Cognitive
ADCS-ADL	Alzheimer's Disease Cooperative Study-Activities of Daily Living
AE	Adverse Event
ANCOVA	Analysis of Covariance
ATRI	Alzheimer's Therapeutic Research Institute
CDR	Clinical Dementia Rating
CMRgl	Cerebral Metabolic Rate for glucose
CRF	Case Report Form
CSF	Cerebrospinal Fluid
DSMB	Data and Safety Monitoring Board
FDG	Fluoro Deoxy Glucose
mITT	Modified Intention-To-Treat
MMRM	Mixed Model of Repeated Measures
MMSE	Mini- Mental State Examination
MRI	Magnetic Resonance Imaging
NPI	Neuropsychiatric Inventory
PET	Positron-Emission Tomography
PK	Pharmacokinetic
SOP	Standard Operating Procedure
SAE	Serious Adverse Event
SAP	Statistical Analysis Plan
SPM	Statistical Parametric Mapping
sROI	statistical region of interest

CONFIDENTIALITY STATEMENT: This report from the USC Alzheimer's Therapeutic Research Institute (ATRI) is for the sole use of the intended recipient(s) and contains confidential and privileged information. Any unauthorized review, use, disclosure, copying or distribution is strictly prohibited.

3 Study Design/Summary

This section is from the Clinical Research Protocol, `FYN Protocol v3.0 - 20160531 - clean.pdf`

3.1 Overview

FYN is a Phase 2a multi-center, randomized, double-blind, placebo-controlled study evaluating F-FDG PET, Safety, and Tolerability of AZD0530 in 152 patients with Mild AD.

3.2 Study Aims

3.2.1 Primary Aims

Aim 1. To assess the effect of treatment with AZD0530 on 52-week reductions in fluorodeoxyglucose positron emission tomography (F-FDG PET) measurements of the cerebral metabolic rate for glucose (CMRgl) using statistical parametric mapping (SPM) statistical region of interest (sROI) in subjects with mild AD.

Aim 2. To assess the safety and tolerability of treatment with AZD0530 over a 52-week period in subjects with mild AD as assessed by analysis of adverse events, including symptoms, and abnormal findings on physical examinations, neurological examinations, standard laboratory tests, and PK analysis of AZD0530.

3.2.2 Secondary Aims

Aim 3. To assess the effect of treatment with AZD0530 on ADAS-cog, MMSE, ADCS-ADL, CDR-SB, and NPI in participants with mild AD.

Aim 4. To assess the effect of treatment with AZD0530 on the rate of change in: total brain volume, ventricular volume, hippocampal volume, and entorhinal thickness, using volumetric magnetic resonance imaging (MRI).

Aim 5. To assess the effect of treatment with AZD0530 on CSF biomarkers of AD (particularly CSF total Tau and CSF pTau).

Aim 6. To assess the influence of APOE genotype on the effects of treatment with AZD0530.

3.3 Study Population

The study will enroll males and females age 55-85 with mild Alzheimer's disease as specified in the entry criteria listed in the Clinical Research Protocol. Subjects who do not meet all inclusion criteria, disease diagnostic criteria, or who meet any exclusion criteria may not be randomized into the study without prior approval from the Project Director (and/or Medical Monitor). Efforts will be made to target at least 20% minority enrollment, which will be facilitated through special minority outreach effort.

3.4 Power and Sample Size Determination

Power Analyses for Aim 1. F-FDG PET
Sample size calculations were based on a two-sided, two-sample t-test to compare the differences in mean change in CMRgl from baseline to month 12 by treatment arm. The statistical software R (version 2.14.0), http://www.r-project.org, was used for all power calculations. Using the reported CMRgl decline mean and standard deviation of 0.0514 +/- 0.0309 in Chen et al., a sample size of 128 participants (64 participants per arm) is needed to detect a treatment effect of 30% with 80% power and two-sided $\alpha = 0.05$. Assuming 15% per year attrition rate, a total of 152 subjects need to be enrolled at the start of the study.

CONFIDENTIALITY STATEMENT: This report from the USC Alzheimer's Therapeutic Research Institute (ATRI) is for the sole use of the intended recipient(s) and contains confidential and privileged information. Any unauthorized review, use, disclosure, copying or distribution is strictly prohibited.

Power Analyses for Aim 2. Safety and Tolerability
Power calculations were based on a two-sided chi-square test for detecting a difference between two proportions assuming a Type I error of 0.05. With a sample size of 64 patients in each group, and assuming an adverse event (AE) rate of 12.5% in the placebo group, there will be 80% power to detect an AE rate of 33.1% in the active treatment group (absolute difference in AE rates of 20.6% or higher). In other words, the AE rate will need to be as high as 33.1% in the active treatment group before there is sufficient power to assert that the result is statistically significant.

3.5 Treatment Allocation

Participants are assigned to either AZD0530 100mg or 125mg, or Placebo tablets taken orally once daily.

CONFIDENTIALITY STATEMENT: This report from the USC Alzheimer's Therapeutic Research Institute (ATRI) is for the sole use of the intended recipient(s) and contains confidential and privileged information. Any unauthorized review, use, disclosure, copying or distribution is strictly prohibited.

4 Study Outcome Variables

4.1 Primary Outcome

F-FDG PET measurements of the cerebral metabolic rate for glucose (CMRgl)

4.2 Secondary Outcomes

- ADAS-Cog (11 items)
- ADCS-ADL
- CDR-SB
- MMSE
- NPI

4.3 Safety Outcomes

- Adverse Events (AEs)
- Serious Adverse Events (SAE)
- Deaths
- Hospitalizations
- Symptoms, and abnormal findings on physical examinations, neurological examinations, and standard laboratory tests.
- PK analysis of plasma AZD0530 (sample drawn less than 12 hours post-dose will be excluded from analysis)

4.4 Biomarker and Imaging Outcomes

- Volumetric MRI outcomes include (i) hippocampal volume, (ii) ventricular volume, (iii) total brain volume, and (iv) entorhinal thickness.
- CSF Biomarkers including total Tau and pTau

5 Sequence of Planned Analysis

5.1 Interim Analysis

AEs and SAEs are being monitored on a regular basis by a Data and Safety Monitoring Board (DSMB). There are no planned interim analysis being conducted for this study, but the DSMB may modify this during ongoing safety monitoring.

5.2 Final Analyses and Reporting

All final planned analyses identified in the clinical research protocol and in this SAP will be performed only after the last patient has completed assessments scheduled for the length of the study period and the database has been cleaned and locked. Any post-hoc exploratory analyses performed to provide support for planned analyses but not identified in this SAP will be documented and clearly identified as unplanned analysis in the manuscript.

CONFIDENTIALITY STATEMENT: This report from the USC Alzheimer's Therapeutic Research Institute (ATRI) is for the sole use of the intended recipient(s) and contains confidential and privileged information. Any unauthorized review, use, disclosure, copying or distribution is strictly prohibited.

6 Statistical Methods

6.1 Analysis Populations

- Primary efficacy modified intention-to-treat (mITT) population: All randomized participants with CMRgl observed at baseline and follow-up.
- Secondary efficacy mITT population: All randomized participants with secondary outcome of interest observed at baseline and at least one follow-up.
- Safety ITT population: All randomized participants.
- Compliers: Subjects with compliance between 80 and 120%, as determined by the ratio of actual to expected pill counts.

6.2 Multiple-testing Strategy

We propose a multiple-testing strategy using a basic serial gatekeeping procedure to maintain over-all experiment-wise Type I error at α = 0.05 (two-sided). The six hypotheses regarding (i) F-FDG PET, (ii) ADAS-Cog, (iii) ADCS-ADL, (iv) CDR-SB, (v) MMSE, and (vi) NPI will be tested in order using a basic serial testing strategy. If, for example, $p_{\mathrm{FDG}} \geq 0.05$, no subsequent hypotheses will be declared statistically significant. Otherwise if $p_{\mathrm{FDG}} < 0.05$, the ADAS-Cog hypothesis is tested by comparing p_{ADAS} to 0.05. This strategy continues until a p-value is ≥ 0.05, or the NPI hypothesis is declared statistically significant with $p_{\mathrm{NPI}} < 0.05$.

Regardless of the outcome of the multiple testing strategy, p-values and 95% confidence intervals will be reported for all planned analyses.

6.3 Incomplete Follow-up/ Missing Data

If any of the individual items for the clinical outcomes are missing, every effort will be made to obtain the score for the missing item or items.

Total scores for assessments with missing item scores will be imputed using a proration strategy. If the maximum score with the non-missing items represents $\geq 70\%$ of maximum possible total score, the total score will be imputed. The score observed with the non-missing items will be prorated to the score with all items, as follows:

$$\text{Imputed score} = \text{Maximum score with all items} \times \frac{\text{Score observed with non-missing items}}{\text{Maximum score with non-missing items}}$$

The imputed score will be rounded to the nearest integer. If the maximum score with the non-missing items represents $< 70\%$ of the maximum score with all items, the total score for the assessment at that visit will not be imputed and will be considered missing.

6.4 Covariates

Analysis models will include fixed effects covariates for age and APOE, in addition to covariates for time and treatment as specified for each hypothesis below.

6.5 Data Management and Analysis Software

Most data manipulation, tables, figures, listings and analyses will be created using R programs and performed using R statistical software (http://www.r-project.org). R version used will be specified in the analysis reports.

CONFIDENTIALITY STATEMENT: This report from the USC Alzheimer's Therapeutic Research Institute (ATRI) is for the sole use of the intended recipient(s) and contains confidential and privileged information. Any unauthorized review, use, disclosure, copying or distribution is strictly prohibited.

7 Statistical Analysis

7.1 Study Flow

A CONSORT style flow diagram will illustrate patient progression through the trial from initial screening for eligibility to completion of the final primary outcome assessment. Number (percentage) will be included for subjects screened, screen failed (with reasons), randomized, study discontinued (with reasons), treatment discontinued (with reasons), analyzed, excluded from analysis (with reasons). The diagram will differentiate treatment groups starting from randomization.

7.2 Patient characteristics and baseline variables

Baseline demographics and clinical characteristics will be summarized by frequencies and percentages for categorical variables, continuous variables will be summarized by mean and standard deviation as well as quartiles. Group comparisons will be performed using Student t-tests for continuous variables. Assumptions of normality (using Shapiro-Wilk tests) and equal variances (using F-tests) will be tested, if violated, the alternative non-parametric Wilcoxon-rank sum test will be used. Fisher's Exact test will be used for categorical data. The following baseline demographic and clinical characteristics will be presented:

- Demographics
  - Age
  - Gender
  - Race
  - Ethnicity
  - Language
  - Marital status
  - Retirement
  - Living situation
  - Education
  - APOE genotype (ε4 carrier vs non-carrier)
- Clinical
  - ADAS-Cog
  - MMSE
  - NPI
  - ADCS-ADL
  - CDR-SB

7.3 Analysis of Primary Outcome

Because the primary outcome is scheduled at two time points, and the secondary outcomes are scheduled at three or five time points, we apply different analytic approaches.

Hypothesis 1

Individuals in the AZD0530 arm will demonstrate smaller rate of CMRgl decline relative to the placebo arm.

The rate of CMRgl change will be compared between the two treatment groups using a linear mixed-effect model with CMRgl at each time point (including baseline) as the outcome variable. The model will include fixed effects for time (as a continuous variable), time-by-treatment interaction, age, and APOE; and participant-specific random intercepts. The model will constrain the two treatment groups to have the same mean at baseline. The hypothesis will be tested using the p-value corresponding to the mean group difference in rate of CMRgl change.

CONFIDENTIALITY STATEMENT: This report from the USC Alzheimer's Therapeutic Research Institute (ATRI) is for the sole use of the intended recipient(s) and contains confidential and privileged information. Any unauthorized review, use, disclosure, copying or distribution is strictly prohibited.

7.4 Analysis of Secondary Outcomes

Hypothesis 2

To assess the safety and tolerability of treatment with AZD0530 over a 52-week period in subjects with mild AD, adverse events, including symptoms and abnormal findings on physical examinations, neurological examinations, standard laboratory tests, and PK data of AZD0530 will be analyzed.

Summary statistics, including frequencies and percentage, will be tabulated for the adverse events (AEs), serious adverse events (SAEs) and clinical laboratory studies. Fisher's Exact Test will be used to compare the frequencies of adverse events between the participants who receive AZD0530 and those receiving placebo. Further, AEs/SAEs will be recorded and categorized by MedDRA System Organ Class (SOC) and Preferred Term (PT), severity, and relationship to imaging, lumbar puncture, investigational product, study procedure(s), Amyvid or concomitant therapy.

Population PK analysis of concentration-time data of AZD0530 will be performed using a Mixed Model of Repeated Measures (MMRM). The outcome measure will be change from baseline in AZD0530 at each follow-up visit. The model will treat time as a categorical variable and include fixed effects for the treatment-by-time interactions, baseline AZD0530, age, and APOE. The model will assume an unstructured correlation and heterogeneous variance with respect to time. If the unstructured correlation model fails to converge, simpler structures (autoregressive of order one and compound symmetric) will be assumed in turn. The hypothesis will be tested using the p-value for the mean group difference in AZD0530 at week 52.

Hypothesis 3

Individuals in the AZD0530 arm will demonstrate less decline at week 52 relative to placebo in clinical and cognitive outcomes including, ADAS-Cog, ADCS-ADL, CDR-SB, MMSE and NPI.

A similar categorical time MMRM as above will be used to estimate the mean group difference in each secondary outcome at 52 weeks. The outcome measure will be change from baseline in the outcome at each follow-up visit. The model will treat time as a categorical variable and include fixed effects for the treatment-by-time interactions and baseline outcome. In addition, age and APOE4 genotype will be included in the model as fixed effect covariates. The model will assume an unstructured correlation and heterogeneous variance with respect to time. If the unstructured correlation model fails to converge, simpler structures (autoregressive of order one and compound symmetric) will be assumed in turn. The hypothesis will be tested using the p-value for the mean group difference at week 52.

7.5 Analysis of Biomarker and Imaging Outcomes

Hypothesis 4

Individuals in the AZD0530 arm will demonstrate improved rate of change in volumetric MRI in comparison to the placebo arm.

The volumetric MRI outcomes will include (i) hippocampal volume, (ii) ventricular volume, (iii) total brain volume, and (iv) entorhinal thickness. The regional volume changes will be determined by nonlinear registration between baseline and follow-up images, and expressed as % deformation per years from baseline. The rate of volumetric MRI change will be compared between the two treatment groups using an ANCOVA model with % deformation per year as the outcome variable and covariates for baseline regional volume (or entorhinal thickness), treatment, age, and APOE. The hypothesis will be tested using the p-value corresponding to the mean group difference in rate of volume change.

Hypothesis 5

Individuals in the AZD0530 arm will demonstrate improved rate of CSF total Tau and pTau change relative to the placebo arm.

Similar to the analysis for Hypothesis 1, the rate of CSF total Tau and pTau change will be compared between the two treatment

CONFIDENTIALITY STATEMENT: This report from the USC Alzheimer's Therapeutic Research Institute (ATRI) is for the sole use of the intended recipient(s) and contains confidential and privileged information. Any unauthorized review, use, disclosure, copying or distribution is strictly prohibited.

groups using a linear mixed-effect model with observations at each time point as the outcome variable. The model will include fixed effects for time (as a continuous variable), time-by-treatment interaction, age, and APOE; and participant-specific random intercepts. The model will constrain the two treatment groups to have the same mean at baseline. The hypothesis will be tested using the p-value corresponding to the mean group difference in rate of change.

Hypothesis 6

APOE genotype will influence the effect of treatment with AZD0530.

This analysis will focus on the potential influence of APOE genotype on the effect of treatment with AZD0530. Analyses of subgroups defined by the presence or absence of the APOEε4 allele (ε4 carriers versus non-carriers) will be performed using the similar analysis techniques describe for Hypotheses 1-5 to obtain separate assessment of treatment efficacy for both ε4 carriers and non-carriers. These models will include fixed effect covariates for time, time-by-treatment interaction, and age.

7.6 Exploratory Subgroup Analyses

All analyses described above will be repeated on the following subgroups:

1. Compliers
2. Quartiles of amyloid SUVR at baseline
3. Median split of MMSE at screen

7.6.1 Quartiles of Drug Exposure

Drug exposure among subjects in active group will be determined by multiplying the median of post-two-week plasma AZD0530 levels times the duration of drug exposure. The above analyses will then be repeated using the quartiles of drug exposure (four groups) instead of randomized treatment group (two groups). In addition to quartile group profiles and contrasts, the main effect of exposure will be tested using a likelihood ratio test comparing the model with versus without the parameters for drug exposure quartiles.

CONFIDENTIALITY STATEMENT: This report from the USC Alzheimer's Therapeutic Research Institute (ATRI) is for the sole use of the intended recipient(s) and contains confidential and privileged information. Any unauthorized review, use, disclosure, copying or distribution is strictly prohibited.

8 Approvals

Approved by	Title	Signature	Date
Christopher van Dyck, MD	Principle Investigator	[signature] MD	4.24.17
Paul Aisen, MD	Director, ATRI	[signature]	5/1/17
Michael Donohue, PhD	Lead Biostatistician	[signature]	5/1/2017

9 Revision History

Version		
Number	Date	Revision Summary
v1.0	20170420	Original

CONFIDENTIALITY STATEMENT: This report from the USC Alzheimer's Therapeutic Research Institute (ATRI) is for the sole use of the intended recipient(s) and contains confidential and privileged information. Any unauthorized review, use, disclosure, copying or distribution is strictly prohibited.
