# Supplementary material for: Effect of AZD0530 on Cerebral Metabolic Decline in Alzheimer Disease: A Randomized Clinical Trial
Source: JAMA Neurol. 2019 Jul 22;76(10):1219–29. doi: 10.1001/jamaneurol.2019.2050 (PMC6646979; doi:10.1001/jamaneurol.2019.2050)
Supplement: Supplement 2. — eMethods eFigure 1. Proportion of Participants Reporting At Least One Adverse Event eFigure 2. Correlation Between 18F-FDG-PET and Clinical Assessments eFigure 3. Forest Plot of Effects for Primary and Secondary Outcomes eFigure 4. Secondary CSF Outcomes [file jamaneurol-76-1219-s002.pdf]

Supplementary Online Content

van Dyck CH, Nygaard HB, Chen K, et al. Effect of AZD0530 on cerebral metabolic decline in Alzheimer disease: a randomized clinical trial. *JAMA Neurol.* Published online July 22, 2019. doi:10.1001/jamaneurol.2019.2050

eMethods

eFigure 1. Proportion of Participants Reporting At Least One Adverse Event

eFigure 2. Correlation Between ^{18}F-FDG-PET and Clinical Assessments

eFigure 3. Forest Plot of Effects for Primary and Secondary Outcomes

eFigure 4. Secondary CSF Outcomes

This supplementary material has been provided by the authors to give readers additional information about their work.

© 2019 van Dyck CH et al. *JAMA Neurology*.

eMethods

Participant Exclusion Criteria

Participants were excluded from the study if they exhibited any significant neurologic disease other than AD or had a screening magnetic resonance imaging scan showing evidence of infection, infarction, or other focal lesions as well as multiple lacunes or lacunes in a brain region critical for memory. Participants were also excluded for clinically significant or unstable medical conditions (including a history of interstitial lung disease), major psychiatric disorder, or a history of alcohol or substance abuse or dependence within the past 2 years (DSM V criteria). Prohibited medications included tricyclic antidepressants, typical antipsychotics, mood-stabilizing psychotropic agents, psychostimulants, opiate analgesics, antiparkinsonian medications, anticonvulsant medications for epilepsy, systemic corticosteroids, central anticholinergics, anticoagulants (CSF substudy), strong CYP3A4 inhibitors or inducers and certain CYP3A4 substrates. Additionally, the following laboratory results were considered exclusionary: neutropenia defined as an absolute neutrophil count of <1,800/µl, thrombocytopenia defined as platelet count $<120\times10^3$/µl, aspartate aminotransferase >1.5×upper limit of normal (ULN); alanine aminotransferase >1.5×ULN; total bilirubin >1.5×ULN; serum creatinine >2.0×ULN.

Rationale for Dose Selection

The dosing plan was based on the results of the Phase 1b trial[1], as well as studies of efficacy and pharmacodynamics of AZD0530 in AD model mice[2]. Among participants in the Phase 1b study, those in the 100 mg dose group with steady state plasma levels above the median value 70

© 2019 van Dyck CH et al. *JAMA Neurology*.

ng/mL (MPI Research laboratory; a calculated free drug concentration of 11.7 nM based on MW of 542 and plasma protein binding of 91%) also attained CSF total drug levels of ≥5 nM[1], within the range of the Fyn Ki for AZD0530 (5-10 nM) and the efficacious levels in AD model mice (5.8-14 nM)[2]. In the present Phase 2a trial, the plasma drug level assay was transferred from the MPI Research laboratory to the ADCS Biomarker Core after cross validation studies demonstrated a tight correlation between plasma AZD0530 values between laboratories, but with the ADCS laboratory values approximately 1.5 times higher than those for the MPI laboratory. Based on these observations, the dosing plan aimed to maximize the number of participants who achieved total plasma AZD0530 levels >100 ng/mL as measured at the ADCS laboratory, while minimizing the safety and tolerability problems that would result from forced titration of all participants to 125 mg daily. From the Phase 1b results, we estimated that approximately 50% of participants starting on 100 mg AZD0530 would be titrated up to 125 mg AZD0530 daily.

Matching of both doses of AZD0530 and Placebo

Participants in the active and placebo arms received identical appearing tablets both before and after dose escalation. This was accomplished by administering one 125 mg tablet (or matched placebo and two 50 mg tablets (or matched placebo) throughout the study. Thus, those participants assigned to 100 mg dose received one placebo-for-125mg tablet and two 50mg AZD0530 tablets; whereas those participants assigned to 125 mg dose received one 125 mg AZD0530 tablet and two placebo-for-50mg tablets. An Interactive Web Response System (IWRS) instructed sites which bottles to dispense based on the participant's treatment arm and dose level assignments.

© 2019 van Dyck CH et al. *JAMA Neurology*.

^{18}F-Florbetapir-PET Methods

PET data were acquired from study participants using ADNI protocols: a 20-min dynamic emission scan was performed, consisting of four 5-min frames in the 3D mode 50 minutes after an intravenous administration of 10 mCi of ^{18}F-Florbetapir, either preceded by a CT scan (for PET/CT scanners) or followed by a transmission scan (for PET-only scanners). Data were corrected for radiation-attenuation and scanner using transmission scans or X-ray CT and reconstructed using reconstruction algorithms. PET data were transferred to the ADCS data portal and retrieved by the team at Banner Alzheimer's Institute imaging facility for central review and analysis following quality control checks and standardization by University of Michigan (eMethods). Eligibility for the study was determined based on both visual and quantitative metrics for elevated amyloid burden. Visual readings from the raw images were based on recently validated algorithms[3] and guidelines provided in the Amyvid package insert[4].

^{18}F-Florbetapir and ^{18}F-FDG-PET Data Management and Quality Control

The University of Michigan under the direction of Robert A. Koeppe, PhD assessed each PET scan for scan quality. Each acquired PET dataset was reviewed and pre-processed using standardized procedures to identify artifacts and minimize scanner-dependent differences in ^{18}F-Florbetapir or ^{18}F-FDG uptake. During the pre-processing, automated algorithms were used to register and average each participant's six (^{18}F-FDG) or four (^{18}F-Florbetapir) 5-min emission frames, transform each registered image into a 160×160×1.5 mm voxel matrix with sections parallel to a horizontal section through the anterior and posterior commissures (without any adjustment for size or shape), normalize the images for individual variations in absolute image intensity, and apply a filter function previously customized for each scanner using a Hoffmann

© 2019 van Dyck CH et al. *JAMA Neurology*.

brain phantom scanned during the site qualification process to ensure an isotropic spatial resolution of 8 mm full-width-at-half-maximum (FWHM).

MRI Protocol

MRI scans were performed using a 1.5 or 3.0 Tesla magnet and took approximately 30 minutes to complete. The protocol for image acquisition included a brief localizer scan, followed by a high-resolution 3D T1 structural series (MPRAGE or IR-SPGR), a T2-weighted series (FLAIR), a diffusion weighted scan and a gradient recalled echo scan. The volumetric analysis procedure included corrections for gradient nonlinearities[5] and intensity non-uniformity[6,7] using methods developed within the Morphometry Biomedical Informatics Research Network (mBIRN; http://www.birncommunity.org/).

Apolipoprotein E genotyping

Apolipoprotein E genotypes were obtained for all participants using real-time polymerase chain reaction restriction fragment length polymorphism analysis. Genomic DNA from blood was extracted using QIAamp DNA blood maxi kit (Qiagen) and apolipoprotein E genotyping performed using Applied Biosystems. The Taq-Man SNP Genotyping assay was run on a Bio-Rad CFX96.

Statistical Methods

This section supplements the main Statistical Analyses in the manuscript. The full Statistical Analysis Plan is available online as AZD0530_SAP_v1.0.

© 2019 van Dyck CH et al. *JAMA Neurology*.

Analysis Populations. The primary efficacy modified intention-to-treat (mITT) population was defined as all randomized participants with CMRgl observed at baseline and follow-up. The secondary efficacy mITT population was defined as all randomized participants with secondary outcome of interest observed at baseline and at least one follow-up. The safety intention-to-treat (ITT) population was defined as all randomized participants. Compliers were defined as subjects with compliance between 80 and 120%, as determined by the ratio of actual to expected pill counts.

Multiple-testing Strategy. We employed a multiple-testing strategy using a basic serial gatekeeping procedure to maintain overall experiment-wise Type I error at α=.05 (two-sided). The six hypotheses regarding (i) ^{18}F-FDG PET, (ii) ADAS-Cog11, (iii) ADCS-ADL, (iv) CDR-SB, (v) MMSE, and (vi) NPI were to be tested in order using a basic serial testing strategy. If, for example, $P_{\mathrm{FDG}} \geq .05$, no subsequent hypotheses would be declared statistically significant. Otherwise if $P_{\mathrm{FDG}} < .05$, the ADAS-Cog11 hypothesis would be tested by comparing $P_{\mathrm{ADAS\text{-}Cog11}}$ to .05. This strategy would continue until a *P*-value is .05, or the NPI hypothesis is declared statistically significant with $P_{\mathrm{NPI}} < .05$. Regardless of the outcome of the multiple testing strategy, *P*-values and 95% confidence intervals would be reported for all planned analyses.

Incomplete Follow-up / Missing Data. If any of the individual items for the clinical outcomes are missing, every effort was made to obtain the score for the missing item or items. Total scores for assessments with missing item scores were imputed using a proration strategy. If the maximum score with the non-missing items represents 70% of maximum possible total score, the total score was imputed. The score observed with the non-missing items was prorated to the score with all items, as follows: Imputed score = (Maximum score with all items)*(Score observed with non-missing items)/(Maximum score with non-missing items). The imputed score

© 2019 van Dyck CH et al. *JAMA Neurology*.

was rounded to the nearest integer. If the maximum score with the non-missing items represented <70% of the maximum score with all items, the total score for the assessment at that visit was imputed and was considered missing.

Patient characteristics and baseline variables. Group comparisons were performed using Student *t* tests for continuous variables and Fisher's Exact test for categorical data.

Hypothesis 1: Individuals in the AZD0530 arm will demonstrate smaller rate of CMRgl decline relative to the placebo arm. The rate of CMRgl change was compared between the two treatment groups using a linear mixed-effect model with CMRgl at each time point (including baseline) as the outcome variable. The model included fixed effects for time (as a continuous variable), time-by-treatment interaction, age, and *APOE*; and participant-specific random intercepts. The model constrained the two treatment groups to have the same mean at baseline. The hypothesis was tested using the *P*-value corresponding to the mean group difference in rate of CMRgl change.

Hypothesis 2: To assess the safety and tolerability of treatment with AZD0530 over a 52-week period in subjects with mild AD, adverse events, including symptoms and abnormal findings on physical examinations, neurological examinations, standard laboratory tests, and PK data of AZD0530 will be analyzed. Summary statistics, including frequencies and percentage, were tabulated for the adverse events (AEs), serious adverse events (SAEs) and clinical laboratory studies. Fisher's Exact Test was used to compare the frequencies of adverse events between the participants who receive AZD0530 and those receiving placebo. Further, AEs/SAEs were recorded and categorized by MedDRA System Organ Class (SOC) and Preferred Term (PT), severity, and relationship to imaging, lumbar puncture, investigational product, study procedure(s), ^{18}F-Florbetapir-PET, or concomitant therapy. Population PK analysis of

© 2019 van Dyck CH et al. *JAMA Neurology*.

concentration-time data of AZD0530 was performed using a Mixed Model of Repeated Measures (MMRM). The outcome measure was change from baseline in AZD0530 at each follow-up visit. The model treated time as a categorical variable and include fixed effects for the treatment-by-time interactions, baseline AZD0530, age, and *APOE*. The model assumed an unstructured correlation and heterogeneous variance with respect to time. If the unstructured correlation model failed to converge, simpler structures (autoregressive of order one and compound symmetric) would be assumed in turn. The hypothesis was tested using the *P*-value for the mean group difference in AZD0530 at week 52.

Hypothesis 3: Individuals in the AZD0530 arm will demonstrate less decline at week 52 relative to placebo in clinical and cognitive outcomes including, ADAS-Cog11, ADCS-ADL, CDR-SB, MMSE and NPI. A similar categorical time MMRM as above was used to estimate the mean group difference in each secondary outcome at 52 weeks. The outcome measure was change from baseline in the outcome at each follow-up visit. The model treated time as a categorical variable and included fixed effects for the treatment-by-time interactions and baseline outcome. In addition, age and *APOE4* genotype were included in the model as fixed effect covariates. The model assumed an unstructured correlation and heterogeneous variance with respect to time. If the unstructured correlation model failed to converge, simpler structures (autoregressive of order one and compound symmetric) would be assumed in turn. The hypothesis was tested using the *P*-value for the mean group difference at week 52.

Hypothesis 4: Individuals in the AZD0530 arm will demonstrate improved rate of change in volumetric MRI in comparison to the placebo arm. The volumetric MRI outcomes included (i) hippocampal volume, (ii) ventricular volume, (iii) total brain volume, and (iv) entorhinal thickness. The regional volume changes were determined by nonlinear registration between

© 2019 van Dyck CH et al. *JAMA Neurology*.

baseline and follow-up images, and expressed as % deformation per years from baseline. The rate of volumetric MRI change was compared between the two treatment groups using an ANCOVA model with % deformation per year as the outcome variable and covariates for baseline regional volume (or entorhinal thickness), treatment, age, and *APOE*. The hypothesis was tested using the *P*-value corresponding to the mean group difference in rate of volume change.

Hypothesis 5: Individuals in the AZD0530 arm will demonstrate improved rate of CSF total Tau and pTau change relative to the placebo arm. Similar to the analysis for Hypothesis 1, the rate of CSF total Tau and pTau change were compared between the two treatment groups using a linear mixed-effect model with observations at each time point as the outcome variable. The model included fixed effects for time (as a continuous variable), time-by-treatment interaction, age, and *APOE*; and participant-specific random intercepts. The model constrained the two treatment groups to have the same mean at baseline. The hypothesis was tested using the *P*-value corresponding to the mean group difference in rate of change.

Hypothesis 6: *APOE* genotype will influence the effect of treatment with AZD0530. This analysis focused on the potential influence of *APOE* genotype on the effect of treatment with AZD0530. Analyses of subgroups defined by the presence or absence of the *APOE*-ε4 allele (ε4 carriers versus non-carriers) was performed using the similar analysis techniques describe for Hypotheses 1-5 to obtain separate assessment of treatment efficacy for both ε4 carriers and non-carriers. These models included fixed effect covariates for time, time-by-treatment interaction, and age.

© 2019 van Dyck CH et al. *JAMA Neurology*.

Exploratory Subgroup Analyses. All analyses described above were repeated on the following subgroups: 1. Compliers, 2. Quartiles of amyloid SUVR at baseline, 3. Median split of MMSE at screen.

Quartiles of Drug Exposure. Drug exposure among subjects in active group was determined by multiplying the median of post-two-week plasma AZD0530 levels times the duration of drug exposure. The above analyses was then repeated using the quartiles of drug exposure (four groups) instead of randomized treatment group (two groups). In addition to quartile group profiles and contrasts, the main effect of exposure was tested using a likelihood ratio test comparing the model with versus without the parameters for drug exposure quartiles.

Changes to Statistical Analysis Plan:

Four versions of the study protocol are available online as AZD0530_Protocol_v1.0, v2.0, v3.0, and v4.0. Protocol_v4.0 (p.60) provides a summary of all changes from Protocol_v1.0 to Protocol_v4.0. The only important changes in study design were in the Statistical Analysis Plan (uploaded as AZD0530_SAP_v1.0) that accompanied Protocol_v4.0. However, these changes were made prior to database lock and did not alter the final results.

Protocol_v4.0 added a serial gatekeeping testing strategy, which had no impact on the results since the first hypothesis regarding ^{18}F-FDG-PET was not rejected. The primary analysis approach also changed from Analysis of Covariance (ANCOVA) to a linear mixed effect model treating time as continuous and assuming a common mean for the two randomized groups at baseline. Protocol_v1.0 also specified that "any covariates including stratification factors that are simultaneously unbalanced at baseline (univariate P<0.10) and associated with the outcome (univariate p<0.15) will be included in the model as observed confounders." Under the plan

© 2019 van Dyck CH et al. *JAMA Neurology*.

specified in Protocol_v1.0, the resulting ANCOVA with a covariate for baseline NPI results in a mean randomized group difference in rCMR-Gl change of -.0033 (*SE*=.0058; *P*=.57), consistent with the linear mixed effect analysis of Protocol_v4.0, that is presented in the final results of the manuscript. Finally, Protocol_v4.0 stipulated a change in one of the secondary clinical outcome measures from the ADAS-Cog12 to the ADAS-Cog11. The MMRM analysis for ADAS-Cog12 finds that the treatment group increased by 7.81 (95% CI, 5.85 to 9.77), vs placebo 6.71 (95% CI, 4.86 to 8.57; *P*=.43), consistent with the results for ADAS-Cog11.

© 2019 van Dyck CH et al. *JAMA Neurology*.

eFigure 1. Proportion of Participants Reporting At Least One Adverse Event

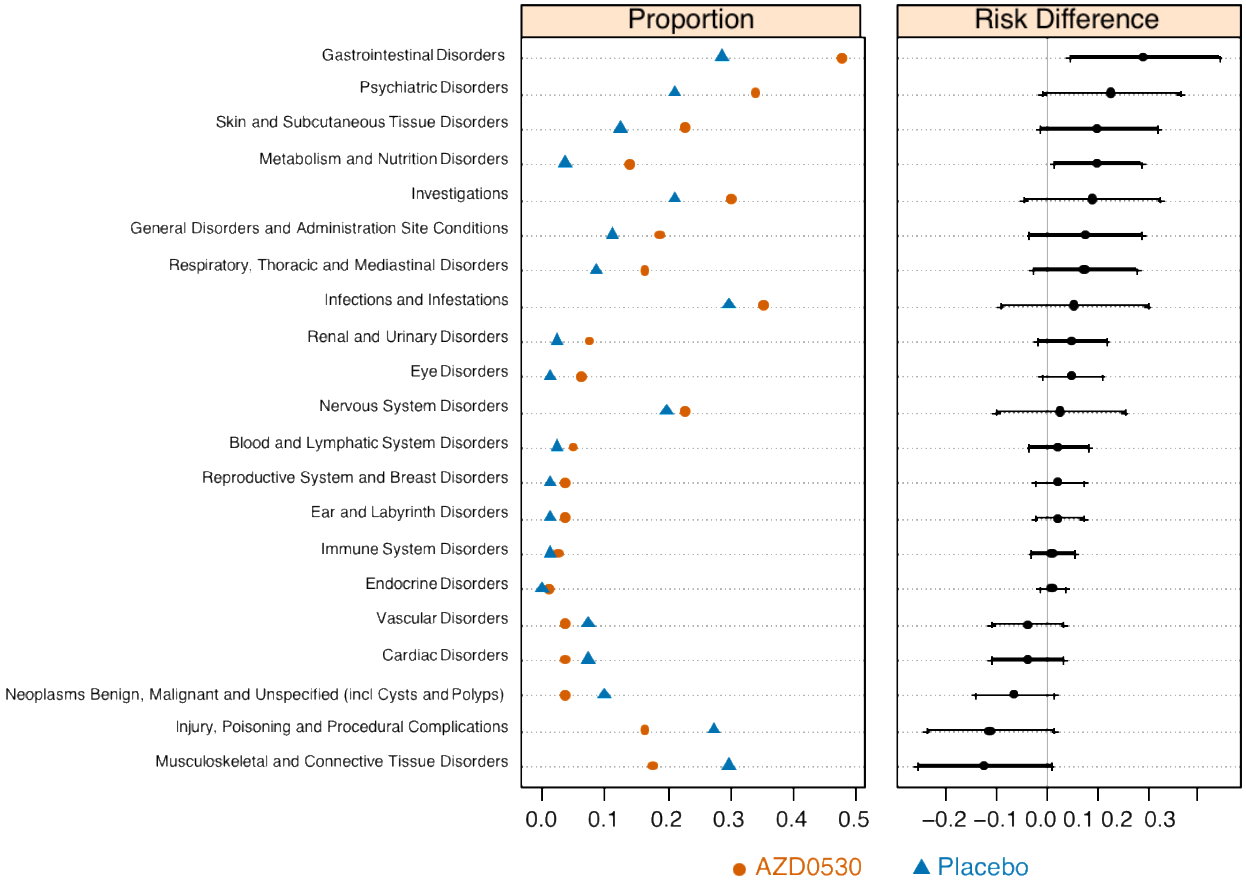

Events are summarized by MedDRA system organ class and sorted in descending order of risk difference. The risk difference is the percentage of people have an adverse event with AZD0530 minus the percentage of people have AE with placebo. Gastrointestinal disorders were significantly more common in the AZD0530 arm (*P*=.015, Fisher Exact Test).

© 2019 van Dyck CH et al. *JAMA Neurology*.

eFigure 2. Correlation Between ^{18}F-FDG-PET and Clinical Assessments

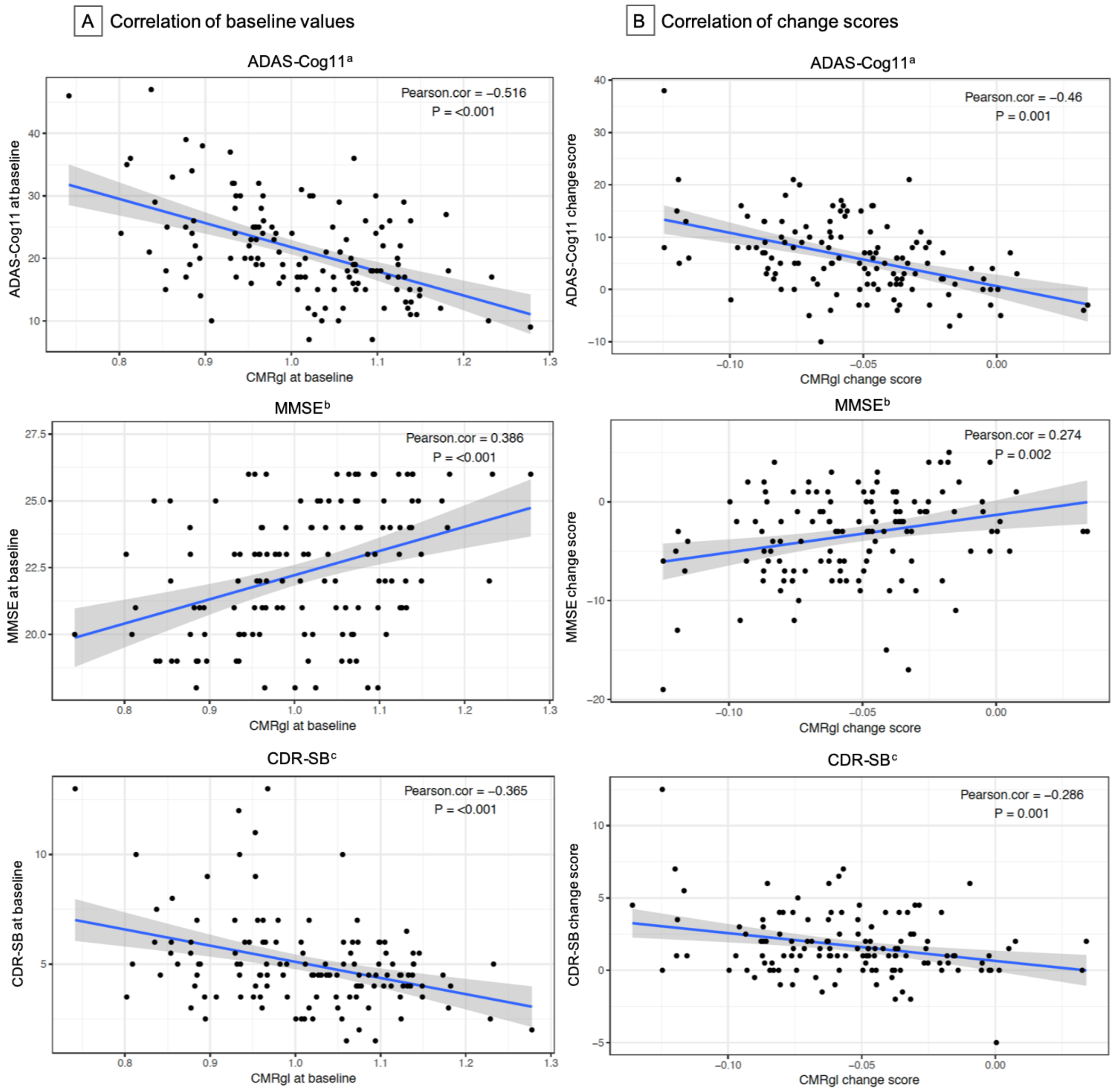

A. Correlation of baseline values. The horizontal axis represents relative CMRgl derived from ^{18}F-Fluorodeoxyglucose (^{18}F-FDG) PET in an AD-related statistical region of interest (sROI) normalized to a spared sROI. B. Correlation of change scores. The horizontal axis represents the change from Baseline to Week 52 in relative CMRgl.

a Alzheimer's Disease Assessment Scale – Cognitive Subscale (ADAS-Cog11) score range, 0 [best] to 70 [worst]).

b Mini Mental State Examination (MMSE) score range, 0 [worst] to 30 [best]).

c Clinical Dementia Rating–sum of boxes (CDR-SB). Score range, 0 [best] to 18 [worst]).

© 2019 van Dyck CH et al. *JAMA Neurology*.

eFigure 3. Forest Plot of Effects for Primary and Secondary Outcomes

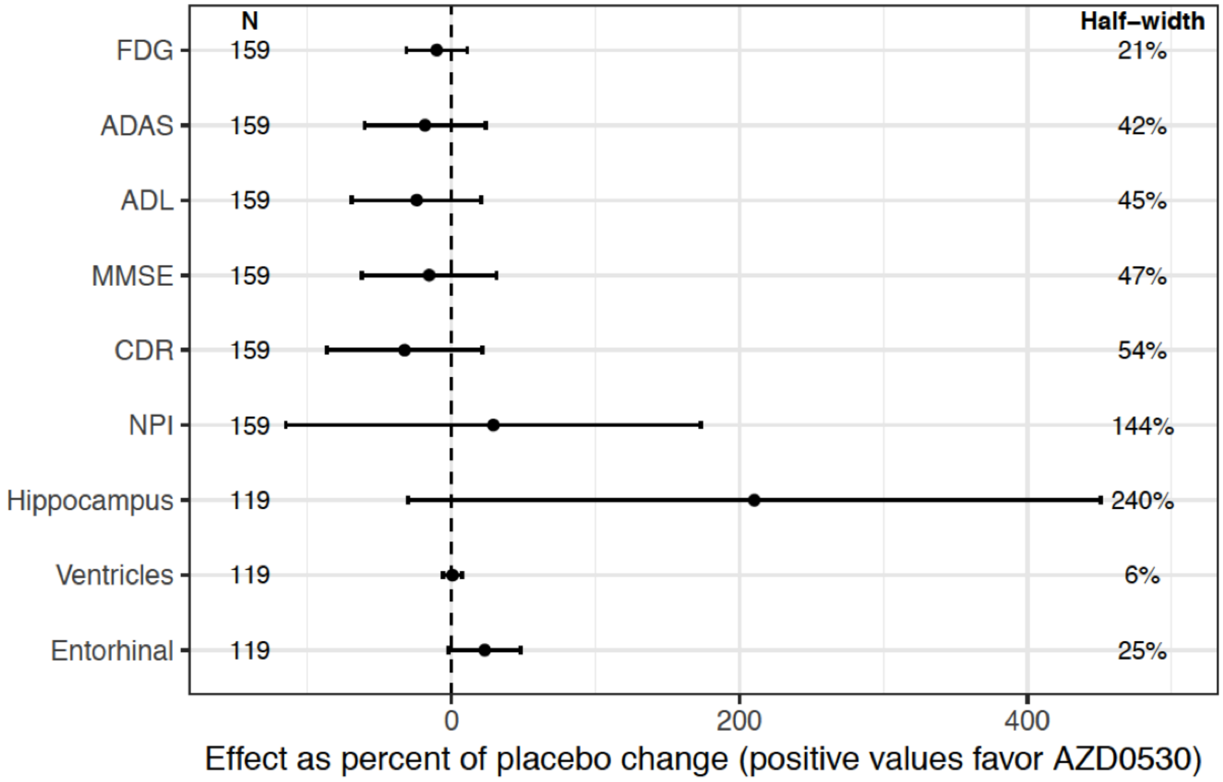

Estimates are derived by dividing the primary model contrast, and its 95% CI, by the estimated change in the placebo group. Resulting confidence intervals on the percentage scale assume the placebo rate of change is known, i.e. the uncertainty in its estimation is ignored.

© 2019 van Dyck CH et al. *JAMA Neurology*.

eFigure 4. Secondary CSF Outcomes

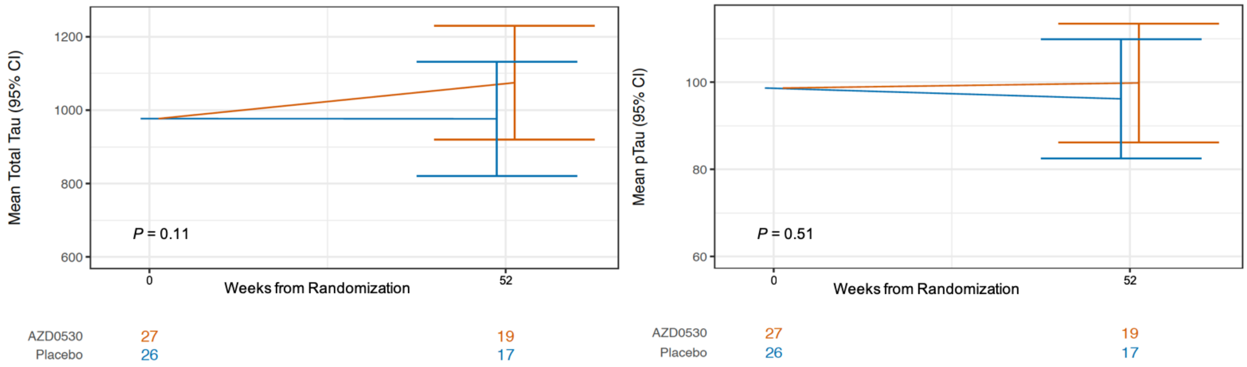

Total Tau and pTau. Analyses of CSF variables used an analysis of covariance (ANCOVA) model, adjusted for mean baseline value, age, and *APOE*-ε4.

© 2019 van Dyck CH et al. *JAMA Neurology*.

REFERENCES:

1. Nygaard HB, Wagner AF, Bowen GS, et al. A phase Ib multiple ascending dose study of the safety, tolerability, and central nervous system availability of AZD0530 (saracatinib) in Alzheimer's disease. *Alzheimers Res Ther.* 2015;7(1):35.
2. Kaufman AC, Salazar SV, Haas LT, et al. Fyn inhibition rescues established memory and synapse loss in Alzheimer mice. *Ann Neurol.* 2015;77(6):953-971.
3. Mintun M, et al. Evaluation of a binary read methodology for florbetapir--PET images. Paper presented at: Human Amyloid Imaging 2012; Miami, FL.
4. Eli Lilly and Company. Amyvid [package insert]. Indianapolis, IN: Lilly USA, LLC; 2012.
5. Jovicich J, Czanner S, Greve D, et al. Reliability in multi-site structural MRI studies: effects of gradient non-linearity correction on phantom and human data. *Neuroimage.* 2006;30(2):436-443.
6. Arnold JB, Liow JS, Schaper KA, et al. Qualitative and quantitative evaluation of six algorithms for correcting intensity nonuniformity effects. *Neuroimage.* 2001;13(5):931-943.
7. Sled JG, Zijdenbos AP, Evans AC. A nonparametric method for automatic correction of intensity nonuniformity in MRI data. *IEEE Trans Med Imaging.* 1998;17(1):87-97.

© 2019 van Dyck CH et al. *JAMA Neurology*.
