# Supplementary material for: Effect of AZD0530 on Cerebral Metabolic Decline in Alzheimer Disease: A Randomized Clinical Trial
Source: JAMA Neurol. 2019 Jul 22;76(10):1219–29. doi: 10.1001/jamaneurol.2019.2050 (PMC6646979; doi:10.1001/jamaneurol.2019.2050)
Supplement: Supplement 3. — Data Sharing Statement [file jamaneurol-76-1219-s003.pdf]

Data Sharing Statement

van Dyck. Effect of AZD0530 on Cerebral Metabolic Decline in Alzheimer Disease. *JAMA Neurol*. Published July 22, 2019.
10.1001/jamaneurol.2019.2050

Data
Data available: Yes
Data types: Deidentified participant data, Data dictionary
How to access data: biostat_request@atrihub.io
When available: With publication

Supporting Documents
Document types: None

Additional Information
Who can access the data: researchers whose proposed use of the data has been approved
Types of analyses: for a specified purpose
Mechanisms of data availability: after approval of a proposal, with a signed data access agreement
